# Chemical Recycling of Commercial Poly(l-lactic acid) to l-Lactide Using a High-Performance Sn(II)/Alcohol Catalyst System

**DOI:** 10.1021/jacs.3c05863

**Published:** 2023-09-01

**Authors:** Thomas M. McGuire, Antoine Buchard, Charlotte Williams

**Affiliations:** † Department of Chemistry, Chemistry Research Laboratory, University of Oxford, 12 Mansfield Road, Oxford, OX1 3TA, U.K.; ‡ Department of Chemistry, Institute for Sustainability, University of Bath, Claverton Down, Bath BA2 7AY, U.K.

## Abstract

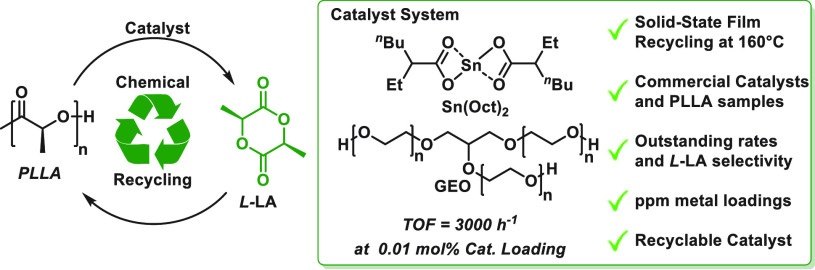

Poly(l-lactic acid) (PLLA) is a leading commercial polymer produced from biomass, showing useful properties for plastics and fiber applications; after use, it is compostable. One area for improvement is postconsumer waste PLLA chemical recycling to monomer (CRM), i.e., the formation of l-lactide (l-LA) from waste plastic. This process is currently feasible at high reaction temperatures and shows low catalytic activity accompanied, in some cases, by side reactions, including epimerization. Here, a commercial Sn(II) catalyst, applied with nonvolatile commercial alcohol, enables highly efficient CRM of PLLA to yield l-LA in excellent yield and purity (92% yield, >99% l-LA from theoretical max.). The depolymerization is performed using neat polymer films at low temperatures (160 °C) under a nitrogen flow or vacuum. The chemical recycling operates with outstanding activity, achieving turnover frequencies which are up to 3000× higher than previously excellent catalysts and applied at loadings up to 6000× lower than previously leading catalysts. The catalyst system achieves a TOF = 3000 h^–1^ at 0.01 mol % or 1:10,000 catalyst:PLLA loading. The depolymerization of waste PLLA plastic packaging (coffee cup lids) produces pure l-LA in excellent yield and selectivity. The new catalyst system (Sn + alcohol) can itself be recycled four times in different PLLA “batch degradations” and maintains its high catalytic productivity, activity, and selectivity.

## Introduction

Chemical recycling to monomer (CRM) is a powerful tool in efforts to circularize the plastic economy.^1,2^ Indeed, recent systemic analyses of the high greenhouse gas emissions for future plastics systems all highlight the imperative for major increases in recycling to preserve both the waste plastic embedded energy and material value.^3−5^ So far, chemical recycling to monomer is most often demonstrated using newly invented polymers, with great potential as future circular materials, but not currently found in existing waste streams.^6−9^ It is equivalently important to develop chemical recycling to true monomer using currently used commercial plastics. Poly(l-lactic acid) (PLLA) is one of the largest commercial, sustainable polymers, produced at 200,000–300,000 tonne/annum, and sourced from crops, via fermentation of starches to l-lactic acid.^10,11^ The lactic acid undergoes polycondensation to form oligoesters, which are thermally decomposed to form the cyclic dimer l-lactide (l-LA).^12^ The ring-opening polymerization (ROP) of l-LA forms PLLA and is catalyzed by Sn(II) alkoxide initiators, formed *in situ* by the reaction of Sn(2-ethyl hexanoate)_2_ (Sn(Oct)_2_) and alcohols.^13−17^l-LA ROP is applied because it is well controlled and yields higher molar mass plastics that are hard to access by condensation routes. High molar mass PLLA is a useful plastic and fiber, and, under controlled conditions, it can be composted; these features make it attractive for use in biodegradable packaging.^18,19^ One detraction of composting as an end-life scenario is that it “wastes” the embedded properties and energy of the polymer. Consequently, various PLLA recycling strategies have also been explored: mechanical recycling is feasible, but PLLA has a narrow processing temperature range, and so this type of recycling accelerates chain degradation.^20^ Thus, mechanical recycling strategies often require the addition of a chain extender to regain material performance postrecycling, but such extenders may interfere with future mechanical recycles.^21^ PLLA hydrolysis or alcoholysis is also sometimes referred to as chemical recycling, but it forms lactic acid or alkyl lactates rather than lactide, which then need to be further processed to access the true monomer. Life cycle analyses of PLLA productions reveal that ∼80% of the process energy input occurs from the plant to production of l-LA, with ∼30% being required for the transformation of lactic acid into l-LA.^22,23^ Therefore, the chemical recycling of PLLA directly to l-LA, i.e., CRM, is important to minimize waste and energy input.

**Figure 1:**
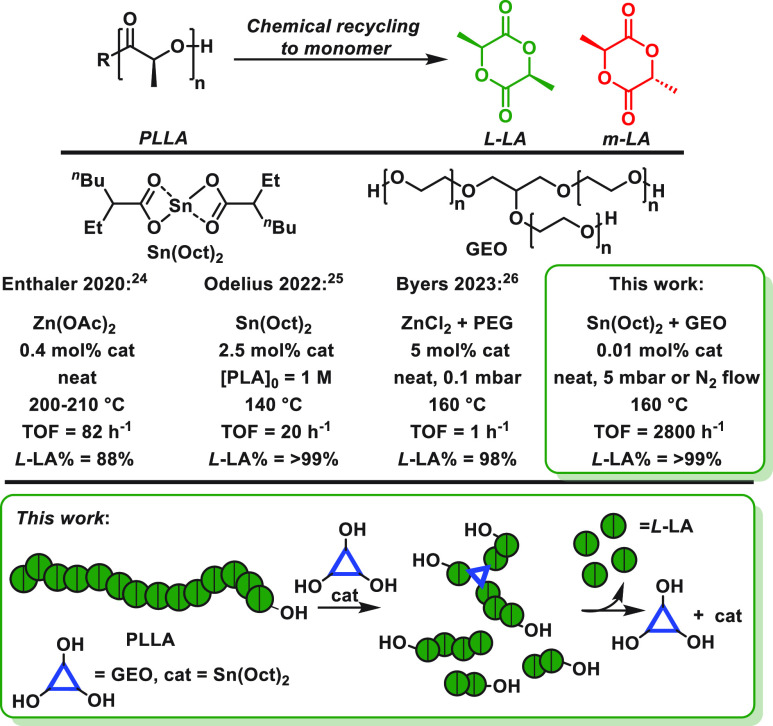
CRM of PLLA to l-LA and the catalyst system used in this work compared with other known catalysts (activities, [PLLA]_0_, and catalyst loadings are calculated per lactic acid and only for reactions where conversions of PLLA > 90%). Bottom: The triol reacts with the PLLA by both transesterification and depolymerization catalysis.

Nevertheless, there are surprisingly few reports of PLLA postconsumer waste recycling to l-LA.^24−26^ Reactions tend to be hindered by side processes, including the epimerization of l-LA to meso-lactide, and operate at elevated temperatures.^27−29^ In 2020, Enthaler and co-workers reported a Zn(OAc)_2_ catalyst for PLLA depolymerization to l-lactide (Figure 1).^24^ The process required relatively high catalyst loading (0.4 mol % or 1:250, Zn(OAc)_2_:PLLA) and temperatures of 200–210 °C. The l-LA was isolated in 98% yield with ∼12% meso-lactide contamination. Nonetheless, the catalytic activity reached an impressive turnover frequency (TOF) of ∼100 h^–1^. In the same study, Sn(Oct)_2_ was also reported to be active for PLLA depolymerization in bulk, although at relatively high loadings and temperatures (0.4 mol %, 200–210 °C). Recently, Odelius and co-workers reported a solution-state PLLA depolymerization to l-LA, catalyzed by Sn(Oct)_2_.^25,30^ By optimizing the reaction solvent, the PLLA ceiling temperature was reduced and the depolymerization equilibrium was driven to l-LA monomer. Accordingly, depolymerizations using 0.5 M solutions of PLLA, in dimethylformamide or γ-valerolactone, formed l-LA at 140 °C. The reaction required very high catalyst loadings of 2.5–10 mol % (1:10–40, Sn(II):PLA), and the separation of l-LA from the solvent significantly reduced the isolated monomer yield. In this work, the aim is to develop PLLA chemical recycling processes that can operate in the melt (*T*_m_ = 130–180 °C) while minimizing the catalyst loading and activation barriers (i.e., reaction temperature). Understanding the depolymerization kinetics will be essential to operate such neat PLLA chemical recycling.

Recently, our team and others have reported on other polymer CRM catalysts operating in neat polymer films and demonstrated the potential to apply thermogravimetric analyses (TGA) to investigate polymerization kinetics.^27,28,31−35^ Pioneering earlier work from Endo and co-workers also applied TGA to investigate catalyzed PLLA depolymerizations and showed that rates were strongly dependent both upon metal type and loading.^36−38^ Cam and co-workers showed that depolymerization rates depend upon PLLA molar mass or degree of polymerization, with the slowest rates occurring for the most useful high molar mass plastics.^39^ This finding hinders practical implementation of chemical recycling of PLLA since higher molar masses are essential to deliver mechanical properties. As such, while oligomeric PLLA is known to be efficiently depolymerized to l-LA, the depolymerization of high molar mass PLLA presents a much greater challenge. We reasoned that chemical recycling might be best achieved by a two-step and one-pot process in which a single catalyst is applied first to PLLA transesterification with added alcohols, to form shorter-chain oligomers, followed by catalyzed CRM using the oligomers to form l-LA. The first process, PLLA transesterification, is very well-known and has been used to upcycle polymer wastes. For example Wang, Xu, and co-workers recently reported an efficient Zn-catalyzed “polymer to polymer” recycling method,^40^ exploiting PLLA transesterification with alcohols to form oligomers (which were subsequently used to make other polymers). While finalizing this article, Byers and co-workers reported PLLA chemical recycling to l-LA, using 10–20 wt % of a ZnCl_2_ catalyst combined with poly(ethylene glycol), PEG.^26^ The catalyst system showed excellent selectivity for l-LA (98%) but very low overall rates with a TOF = 1 h^–1^. Our objective was to discover highly active catalysts and develop applicable processes for chemical recycling of waste PLLA to l-LA. We were also motivated to apply methods reported for the efficient chemical recycling of polycarbonates, i.e., depolymerizations conducted neat using catalysts dispersed in polymer films, under nitrogen flow, since these resulted in high yields and selectivity for monomer.^34^ We rationalized that PLLA recycling to l-LA should also be feasible under such conditions and that the low temperatures and lack of solvent and nitrogen flow might benefit future larger-scale chemical recycling to monomer processes.

## Results and Discussion

**Figure 2:**
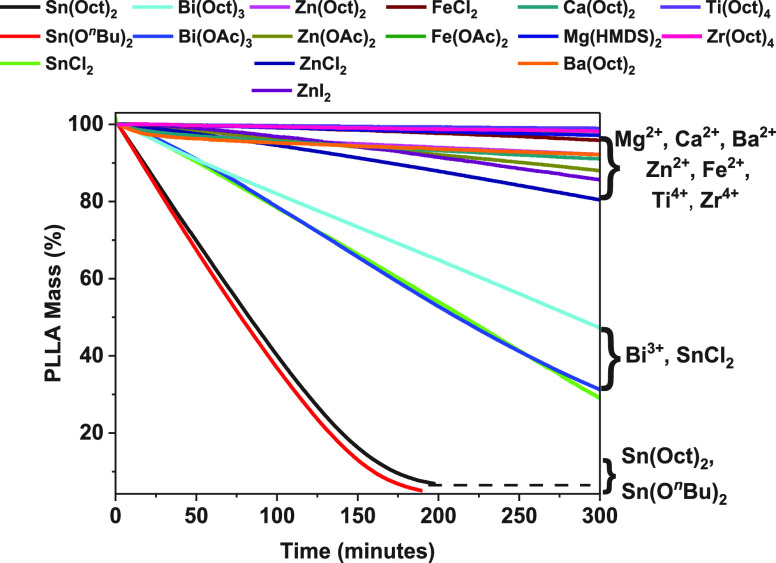
Solid-state PLLA chemical recycling to monomer. Reactions were conducted using different metal salts (at 1:1000, salt: PLLA, at 160 °C). Plots of PLLA mass loss vs time (min) have *k*_obs_ as the gradients of linear fits.

First, a systematic series of Lewis acidic metal salts was investigated, under comparable conditions, to identify the fastest and most selective catalysts. To ensure applicability of the methods, high molar mass, commercial PLLA was applied with the sample showing *M*_n,SEC_ = 60,000 g mol^–1^ and substantial crystallinity (ca. 34%, *T*_m_ = 152 °C, *T*_c_ = 124 °C, Figures S1–S5). The sample contained 95% l- and 5% meso-lactide, as is common in commercial PLLA grades,^18,19^ and was determined to be monohydroxy terminated (see Figure S6 and the SI for further discussion). In the absence of any catalyst, this PLLA showed a temperature of 5% mass loss (*T*_d5_) of 322 °C. Films comprising catalyst:PLLA at fixed loadings of 1:1000 (where 1000 = number of lactic acid repeat units) were prepared by solvent casting (THF) and were carefully dried to remove any solvent residues. The PLLA chemical recycling was evaluated in the TGA instrument, using nitrogen flow rates of 25 mL/min, at 160 °C. Most of the catalysts were effective for the PLLA depolymerizations, and typical experiments showed a linear evolution of polymer mass loss over time (Figure 2). These findings suggest the depolymerization rate is zero-order in polymer mass. The recycling rate constants (*k*_obs_) were used to compare the catalysts; these were determined as the gradients of linear fits to plots of PLLA mass vs time. Among the series of metal salts investigated, Sn(Oct)_2_ showed significantly faster rates than any other metal. It showed TOFs from 342 to 410 h^–1^ and *k*_obs_ = 38.2 ± 2.7 h^–1^ (Table 1, entries 1–3, Table S2, Figures S7–S21). All catalysts, and particularly Sn(Oct)_2_, were active at significantly lower temperature than those for PLLA thermolysis (*T*_d5_ = 322 °C for PLLA), a clear signal for depolymerization catalysis. TGA-FTIR analysis during the depolymerization of PLLA, catalyzed by Sn(Oct)_2_, identified lactide as the sole product (Figure S22). To confirm the formation of l-LA as the product, depolymerizations were also conducted at larger scale using polymer films on glass flasks and using a sublimation apparatus to collect the l-LA. As such, a film comprising PLLA and 0.1 mol % Sn(Oct)_2_ was heated to 160 °C, in a Schlenk tube, and the l-LA was collected onto the coldfinger (Figure S23). The l-LA was isolated in 92% yield and showed the same 5% meso-lactide as was present in the starting polymer; that is, there was no evidence for any epimerization reactions (Figures S24 and S25, as determined by ^1^H NMR spectroscopy and GC-MS). The remarkable selectivity of Sn(Oct)_2_ catalyst was further evidenced through additional depolymerizations conducted using pure PLLA (i.e., 100% poly(l-lactic acid)), and these resulted in the formation of 100% l-lactide, which was isolated in 90% yield (Figures S26 and S27).

**Table 1:** Comparisons between Catalysts for PLLA Chemical Recycling to l-LA, Using Sn(Oct)_2_, Zn(Oct)_2_, and Ca(Oct)_2_^a^

entry	catalyst	loading^b^	temp (°C)	*k*_obs_ (h ^–1^)^c^	TOF (h ^–1^)^d^	mass loss rate (g g ^–1^ h^–1^)^e^	% l-LA^f^
1	Sn(Oct)_2_	1:1000	160	38.2 (±2.7)	380 (±28)	67 (±5)	>99
2	Zn(Oct)_2_	1:1000	160	3.2 (±0.2)	15 (±1)	3 (±1)	-
3	Ca(Oct)_2_	1:1000	160	1.4 (±0.1)	17 (±1)	4 (±1)	-
4	Sn(Oct)_2_	1:1000	170	80.9 (±5.1)	788 (±58)	139 (±11)	>99
5	Sn(Oct)_2_	1:1000	180	152.2 (±23.7)	1440 (±240)	269 (±42)	>99
6	Sn(Oct)_2_	0.5:1000	160	18.2 (±2.6)	370 (±50)	72 (±1)	>99
7	Sn(Oct)_2_	0.33:1000	160	12.3 (±1.9)	380 (±50)	73 (±6)	>99
8	Sn(Oct)_2_	0.25:1000	160	9.8 (±1.0)	400 (±40)	72 (±7)	>99
9	Sn(Oct)_2_	0.2:1000	160	7.8 (±1.7)	374 (±50)	66 (±10)	>99
10	Sn(O^*n*^Bu)_2_	0.1:1000	160	38.6 (±2.4)	381 (±28)	104 (±8)	>99

a Depolymerization experiments conducted using thin films, analyzed using TGA over 5 h or until >95% mass loss (see SI for details of experimental setup).

b Catalyst loadings calculated per *M*_r_ of PLLA repeat unit (*M*_r_ = 72.06 g mol^–1^).

c Rate constant is the gradient of the linear fits to plots of %PLLA mass loss vs time. Average errors are determined from repeat runs see SI for details.

d Activity as TOF defined as moles of lactic acid repeat unit consumed from 0 to 80% mass loss/mol of catalyst/time taken for 0–80% mass loss. In a few cases, 80% mass loss was not reached, and in these cases, TOF is reported for the appropriate conversion at 5 h. Average errors were taken from repeat runs.

e Mass loss rate = TOF × *M*_r_ of lactic acid repeat unit (72.06)/*M*_r_ of catalyst.

f Selectivity for monomer, % L-LA, was determined by ^1^H NMR spectroscopy and GC-MS of monomer isolated from sublimation depolymerization experiments (see SI for details). 5% meso-LA formed from 5% d-lactic acid repeat units in the PLLA sample.

**Figure 3:**
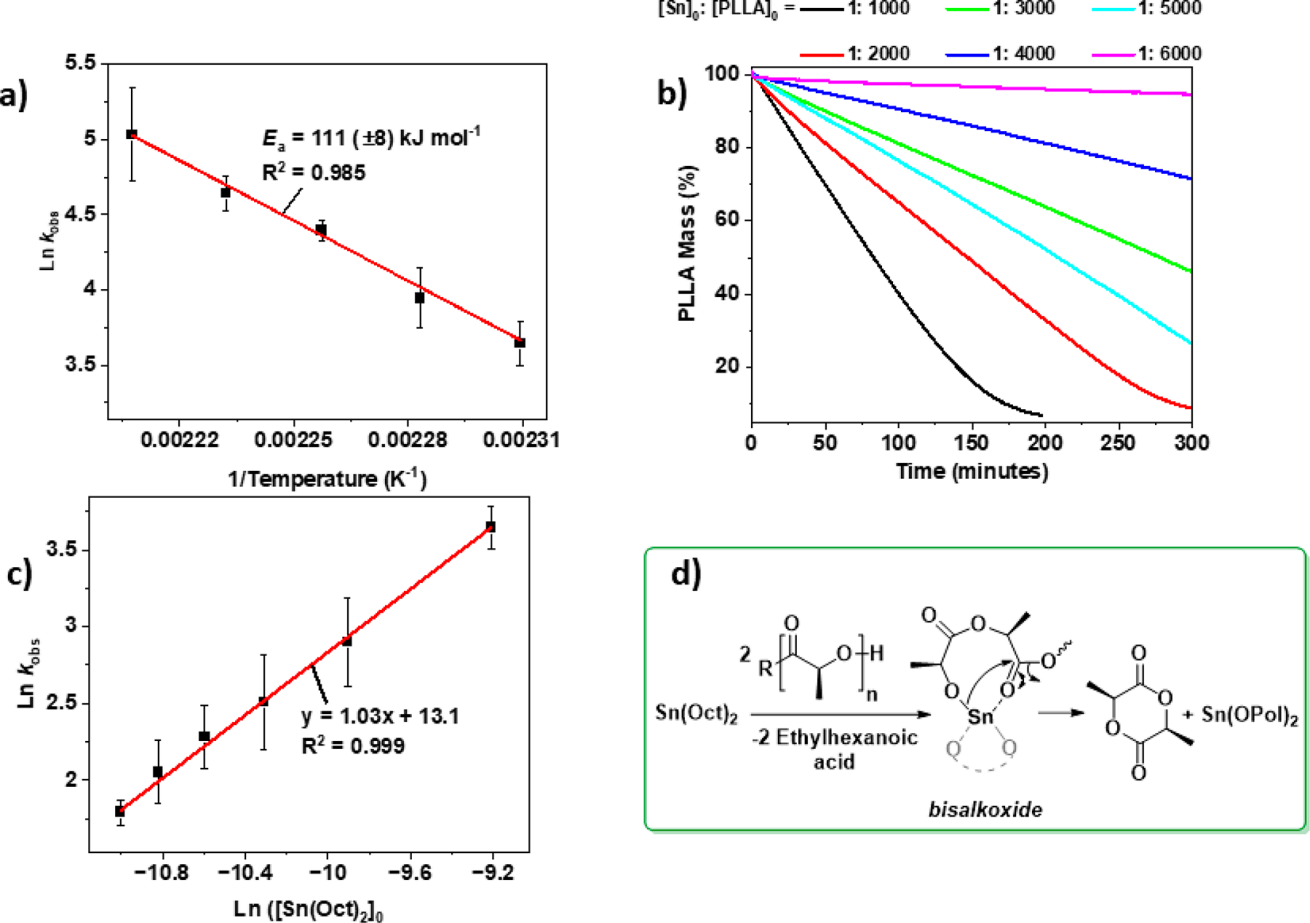
Data for Sn(Oct)_2_-catalyzed chemical recycling of PLLA to l-LA. (a) Arrhenius plot for PLLA depolymerizations allowing determination of the activation energy (*E*_a_). Plot of ln(*k*_obs_) vs 1/*T* applies data collected at 160–180 °C; reactions repeated in triplicate; the errors are determined as the standard deviations of the mean. (b) Plots of PLLA mass loss vs time using various Sn(Oct)_2_ loadings. Experiments were conducted using 1:1000, 1:2000, 1:3000, 1:4000, 1:5000, and 1:6000 Sn:PLLA loadings, at 160 °C; reactions were repeated in triplicate. (c) Determination of the dependence of rate on catalyst concentration. Plots of ln(*k*_obs_) vs ln([Sn(Oct)_2_]_0_); the errors are determined from triplicate runs as the standard deviations of the mean. (d) Potential mechanism for Sn(Oct)_2_-catalyzed PLLA depolymerization.

With Sn(Oct)_2_ identified as the most active and selective PLLA depolymerization catalyst, the investigation focused on testing the catalysis limits and elucidating the rate law. Conducting the depolymerizations at higher temperatures accelerated rates; for example, at 180 °C the TOF was 1650 h^–1^ and the l-LA formed without any epimerization (Table 1, entries 4, 5, Table S3, and Figures S28–S33). The depolymerization activation barrier was determined by an Arrhenius analysis (Figure 3a). The plot of ln(*k*_obs_) vs reciprocal temperature (1/*T*) revealed a transition-state barrier of 111 kJ mol^–1^ (26.5 kcal mol^–1^). The value is identical, within error, to values determined by the teams of Endo and Leiper, who applied dynamic thermal analyses.^33,36^

Reducing the catalyst loading, from 1:1000 to 0.2:1000, allowed for efficient chemical recycling with equivalently high LA selectivity but slightly lower rates (Table 1, entries 1 and 5–9, Figure 3b, Table S4, Figures S34–S42). The plot of ln(*k*_obs_) against ln([Catalyst]) was linear with a gradient of ∼1 (Figure 3c). These data suggest that the recycling rate is first-order in catalyst concentration. The PLLA CRM was also successful when using Sn(II)(O^*n*^Bu)_2_ as the catalyst and showed a near identical rate to CRM using Sn(Oct)_2_ (Table 1, entry 10, Figures S43–S45). The data suggest the two catalysts operate with the same active site, and it is proposed that the true catalyst is a Sn(II)(OR)_2_ complex (R = PLLA). The chemical recycling is a form of depolymerization and may occur either by a chain-end backbiting mechanism, whereby for each lactide unit generated the chain is shortened by one repeat unit, or by a random chain scission in which chains release lactide from any ester group along the backbone (or by a combination of both processes). To investigate the recycling mechanism, a PLLA sample in which the chains were end-capped with an acetate group (PLLA-OAc) was prepared. The chemical recycling of PLLA-OAc was conducted under identical conditions to PLLA CRM but showed significantly lower rates, ∼4 times slower (Figure S46). This finding suggests that polyester chain-end backbiting is the dominant mechanism for CRM. It is proposed that the PLLA hydroxyl end group reacts with the Sn(II) catalyst, likely via an equilibrium process, to form the true Sn(II) alkoxide catalyst (Figure 3d). The flow of nitrogen in the recycling experiments likely helps to drive the equilibrium and may remove some of the liberated 2-ethylhexanoic acid. It is proposed that the Sn(II) alkoxide attacks the PLLA chain by an intramolecular transesterification process to extrude an l-LA molecule and form a new Sn(II) alkoxide intermediate with a chain-shortened PLLA. The presence of equilibria forming Sn(II) alkoxides from Sn(Oct)_2_ and alcohols will be familiar to many researchers since it is the same equilibrium investigated by Penzec,^13,14,41^ Kricheldorf,^42,43^ and others^44−47^ as applicable to the catalysts for l-LA ROP.

Despite the strong performance of the Sn(Oct)_2_ catalyst system, when using commercial PLLA at lower catalyst loadings, the rates were significantly reduced. For instance, at 0.1:1000 [Sn(Oct)_2_]_0_:[PLA]_0_ only ∼5% of l-LA was observed after 5 h (Table S4). It was clear that we needed to improve rates for high molar mass PLLA CRM.

**Figure 4:**
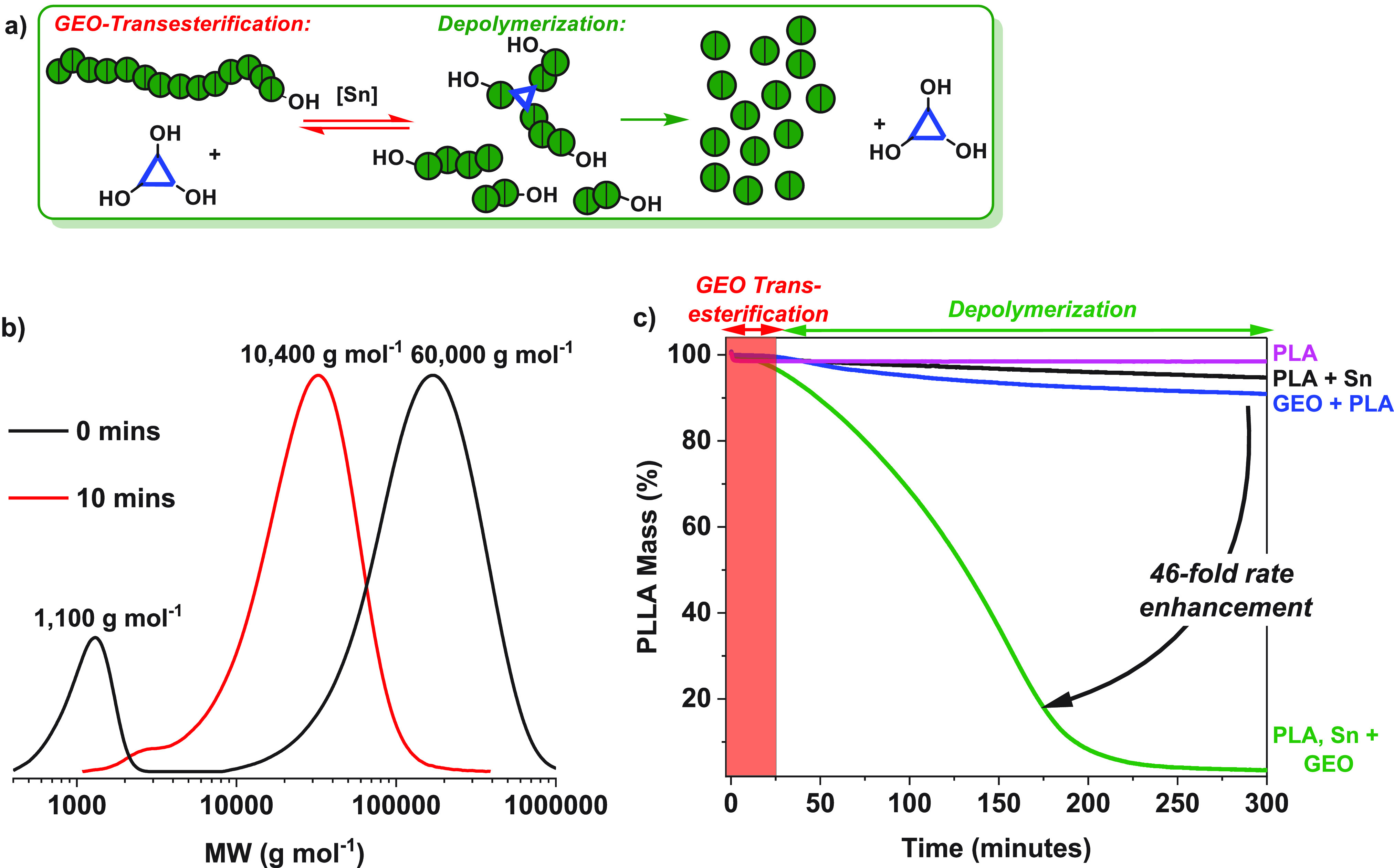
Depolymerization catalysis using Sn(Oct)_2_ and glycerol ethoxylate (GEO). (a) Strategy to accelerate rates in high molar mass PLLA chemical recycling by combining alcohol transesterification reactions to produce lower molar mass PLLA, followed by chemical recycling to l-LA, by chain-end backbiting mechanisms (l-LA molecules represented by green circles). (b) SEC chromatograms illustrating the changes in molar mass during PLLA intermolecular transesterifications with GEO. Experiments were performed in sealed vials at 160 °C, using [Sn(Oct)_2_]_0_:[OH_GEO_]_0_:[PLA]_0_ = 1:20:1000. In the traces at time = 0 (black data set) two peaks are observed (the higher peak is assigned to PLLA and shows *M*_n,SEC_ = 60,000 g mol^–1^, while the lower peak is assigned to GEO, *M*_n,SEC_ = 1100 g mol^–1^). After 10 min (red data set), a single peak is observed at *M*_n,SEC_ = 10,400 g mol^–1^, which is assigned to the transesterified GEO-PLLA chains. N.B. PLLA molar mass are reported using a correction factor of 0.58 to account for the polystyrene standards used to calibrate the SEC.^49^ (c) Plots of PLLA mass loss vs time for different catalyst systems. All experiments were conducted using PLLA films, at 160 °C and with TGA methodology. The isotherms represent PLLA (magenta), PLLA and Sn(Oct)_2_ at [Sn(Oct)_2_]_0_:[PLLA]_0_ = 0.125:1000 (black), PLLA and GEO at [OH_GEO_]_0_:[PLLA]_0_ = 20:1000 (blue), and PLLA, Sn(Oct)_2_, and GEO at [Sn(Oct)_2_]_0_:[OH_GEO_]_0_:[PLLA]_0_ = 0.125:20:1000 (green). The 46-fold rate enhancement occurs only for the catalyst system comprising all three components.

As mentioned in the Introduction, it is known that oligomeric and low molar mass polymers typically depolymerize faster than higher *M*_n_ samples.^39,48^ It is also well established that high molar mass PLLA can react by intermolecular transesterification with alcohols to form lower molar mass/oligomeric chains. Thus, we reasoned that the Sn(II) catalysts enable both the PLLA transesterification with alcohols to form lower molar mass chains and subsequent accelerated chemical recycling to lactide. The alcohol applied must be soluble in the typical solvents used to cast the PLLA films, e.g., dichloromethane (DCM) or tetrahydrofuran (THF). It must also be nonvolatile and efficient in PLLA transesterification. A range of common alcohols have been used in forward polymerization, e.g., 4-methyl benzyl alcohol, 1,4-benzene dimethanol, and 1,1,1-trishydroxymethylpropane, but these were all too volatile for use in depolymerization (Figures S47 and S48). The low solubility of common multifunctional alcohols used to produce “star” PLLA, e.g., pentaerythritol, is undesirable in forming homogeneous catalyst/PLLA films. Thus, our attention turned to a commercial triol, glycerolethoxylate (GEO), which shows an *M*_n_ ∼ 1000 g mol^–1^ and high THF solubility and which is nonvolatile. To evaluate its potential, first, the Sn(Oct)_2_ catalyst was investigated in GEO transesterification with the commercial PLLA sample (Figure 4a). Films comprising 1:6.7:1000 loadings of Sn(Oct)_2_:GEO:PLLA were cast into glass vials, with the solvent used for casting being removed *in vacuo* (N.B. each GEO features three hydroxyl groups so the loading per hydroxyl is [Sn(Oct)_2_]_0_:[OH_GEO_]_0_:[PLLA]_0_ = 1:20:1000). As hydroxyl groups are the key functionality in any transesterification and also in the chemical recycling mechanism, all loadings are subsequently reported per OH_GEO_ group. The vials were sealed, which ensured that only the transesterification reactions occurred without significant l-LA formation, even at higher temperatures. At the start of the reaction, i.e., time = 0, the films were analyzed by SEC, which revealed two different peaks, PLLA at 60,000 g mol^–1^ and GEO at 1100 g mol^–1^ (Figure 4b). Heating the reaction at 160 °C for only 10 min caused a significant change: a single peak was observed consistent with GEO-PLA transesterification, with *M*_n,SEC_ = 10,400 g mol^–1^. The average PLLA chain length decreased as expected, and the majority of the GEO reacted with the PLLA. Furthermore, analysis of the transesterification product by ^1^H, ^13^C, ^1^H-^13^C HSQC, and HMBC NMR spectroscopy revealed the presence of junction units between the GEO and PLLA segments (Figures S49–S51). The data indicate that Sn(Oct)_2_ catalyzes the rapid transesterification of PLLA with GEO at 160 °C. Encouraged by these results, the Sn(Oct)_2_-catalyzed chemical recycling of PLLA was conducted with additional GEO. Films comprising Sn(Oct)_2_:OH_GEO_:PLLA = 1:20:1000 were cast in the TGA crucibles. The chemical recycling to l-LA was found to occur significantly faster than reactions conducted without the alcohol, and the recycling showed a TOF^GEO^ = 715 h^–1^ and *k*_obs_^GEO^ = 74.8 h^–1^ (Table 2, entry 1, Figure S52). These rates are 2 times higher than the equivalent processes conducted without alcohol, *k*_obs_^GEO^/*k*_obs_ = 2.0. Moreover, at fixed [OH_GEO_]_0_:[PLLA]_0_ loadings of 1:50, the activity of the catalyst increased as the Sn(Oct)_2_ loading was decreased (Figures S53–S57). As such, chemical recycling conducted using Sn(Oct)_2_:PLLA of 0.5:1000 or 0.25:1000 showed a 3–4× higher rate than equivalent CRM conducted without alcohol (Table 2, entries 2 and 3). At Sn(Oct)_2_:PLA = 0.125:1000 there was a 46-fold rate enhancement compared to the equivalent reaction without any alcohol (Figure 4c, Table 2, entry 5). The chemical recycling even proceeded effectively at very low catalyst loadings, including Sn(Oct)_2_:PLLA = 0.1:1000 or 1:10,000, i.e., 0.01 mol %, maintaining excellent rates, catalytic activity, and high selectivity for l-LA (Table 2, entry 6). The important feature to note in these experiments is that by holding the OH_GEO_:PLLA ratio constant at 1:50, the extent of transesterification, and hence the PLLA chain length, is also constant. Given the dramatic increases in rates observed as the catalyst concentration is reduced, even when using chains of equivalent length, the data suggest the alcohol functionalities in GEO reduce the overall depolymerization catalysis barrier (*vide infra*). Next, the influences upon rates of the concentration of the OH groups were investigated by systematically varying the OH_GEO_ loading when fixing the Sn(Oct)_2_:PLA at 0.25:1000 (Figures S58–S62). Increasing the OH concentration, from Sn(Oct)_2_:OH = 0.25:10 to 0.25:30, increased the recycling rates (Table 2, entries 3, 7, and 8).

**Table 2:** Data for PLLA Chemical Recycling to l-LA Using Sn(Oct)_2_ and GEO Catalyst Systems Compared to Literature Chemical Recycling Catalysts at 160 °C^a^

entry	[cat]_0_:[OH_ROH_]_0_:[PLA]_0_^b^	rate constant *k*_obs_^GEO^ (h ^–1^)^c^	activity, TOF (h ^–1^)^d^	mass loss rate (g g^–1^ h^–1^)^e^	selectivity % l-LA^f^	rate enhancement *k*_obs_^GEO^/*k*_obs_^g^
1	1:20:1000	74.8(±7.5)	715(±75)	129(±13)	>99	2.0(±0.1)
2	0.5:20:1000	67.5(±6.9)	1130(±84)	200(±15)	>99	3.7(±0.2)
3	0.25:20:1000	42.1(±2.4)	1590(±117)	285(±21)	>99	4.3(±0.1)
4	0.2:20:1000	44.2(±2.5)	1640(±121)	202(±15)	>99	5.7(±0.2)
5	0.125:20:1000	36.4(±2.1)	2200(±162)	396(±30)	>99	45.5(±0.1)
6	0.1:20:1000	31.5 (±2.5)	2700(±130)	474(±23)	>99	28.6(±0.2)
7	0.25:10:1000	35.1(±3.2)	1350(±100)	220(±17)	>99	3.0(±0.1)
8	0.25:30:1000	90.1(±5.1)	3000(±220)	534(±40)	>99	9.2(±0.1)
9	0.25:80:1000	86.5(±10.0)	2800(±200)	491(±38)	>99	8.7(±0.2)
10^h^Sn(Oct)_2_	50:–:1000	-	17	-	93	-
11^i^ Sn(Oct)_2_	25:–:1000	-	20	-	99	-
12^j^Zn(OAc)_2_	4.0:–:1000	-	-	-	-	-
13^k^Zn(OAc)_2_	4.0:–:1000	-	82	-	88	-
14^l^ZnCl_2_/PEG	50:62:1000	-	1	-	98	-

a Chemical recycling experiments were conducted using thin films of PLLA and analyzed using TGA, over 5 h or until >95% mass loss (see SI for experimental details).

b Catalyst loadings determined per lactic acid repeat unit (*M*_r_ = 72.06 g mol^–1^) and [OH_ROH_]_0_ = 3[GEO]_0_ and 2[PEG]_0_.

c Rate constant determined as the gradient of linear fits to plots of PLLA mass loss vs time. Error ranges are determined from multiple repeat experiments.

d TOF = activity and defined as moles of lactic acid repeat unit consumed from 0 to 80% mass loss/mol of catalyst/time. Errors were determined from multiple repeat experiments.

e Mass loss rate = TOF × *M*_r_ of lactic acid repeat unit (72.06)/*M*_r_ of catalyst.

f Selectivity for monomer, % l-LA, was determined by ^1^H NMR spectroscopy and GC-MS using l-LA isolated from larger scale chemical recycling experiments (collected by sublimation, see SI for more experimental details). 5% meso-LA formed from 5% d-lactic acid repeat units in PLLA sample.

g Rate enhancement for chemical recycling conducted with additional alcohol reported as *k*_obs_^GEO^/*k*_obs_. Rate constants are reported under equivalent experimental conditions and at constant catalyst loading.

h Data reported in ref (25). Recycling conditions: [PLLA]_0_ = 1.0 M in DMSO, 5 mol % Sn(Oct)_2_, 160 °C, 1 h.

i Data reported in ref (25). Recycling conditions: [PLLA]_0_ = 1.0 M in DMF, 2.5 mol % Sn(Oct)_2_, 140 °C, 2 h.

j Data reported in ref (24). Recycling conditions: neat PLLA, 0.4 mol % Zn(OAc)_2_, 160 °C.

k Data reported in ref (24). Recycling conditions: neat PLLA, 0.4 mol % Zn(OAc)_2_, 200–210 °C.

l Data reported in ref (26). Recycling conditions: neat PLLA, 5 mol % ZnCl_2_, 6 mol % PEG, 160 °C.

**Figure 5:**
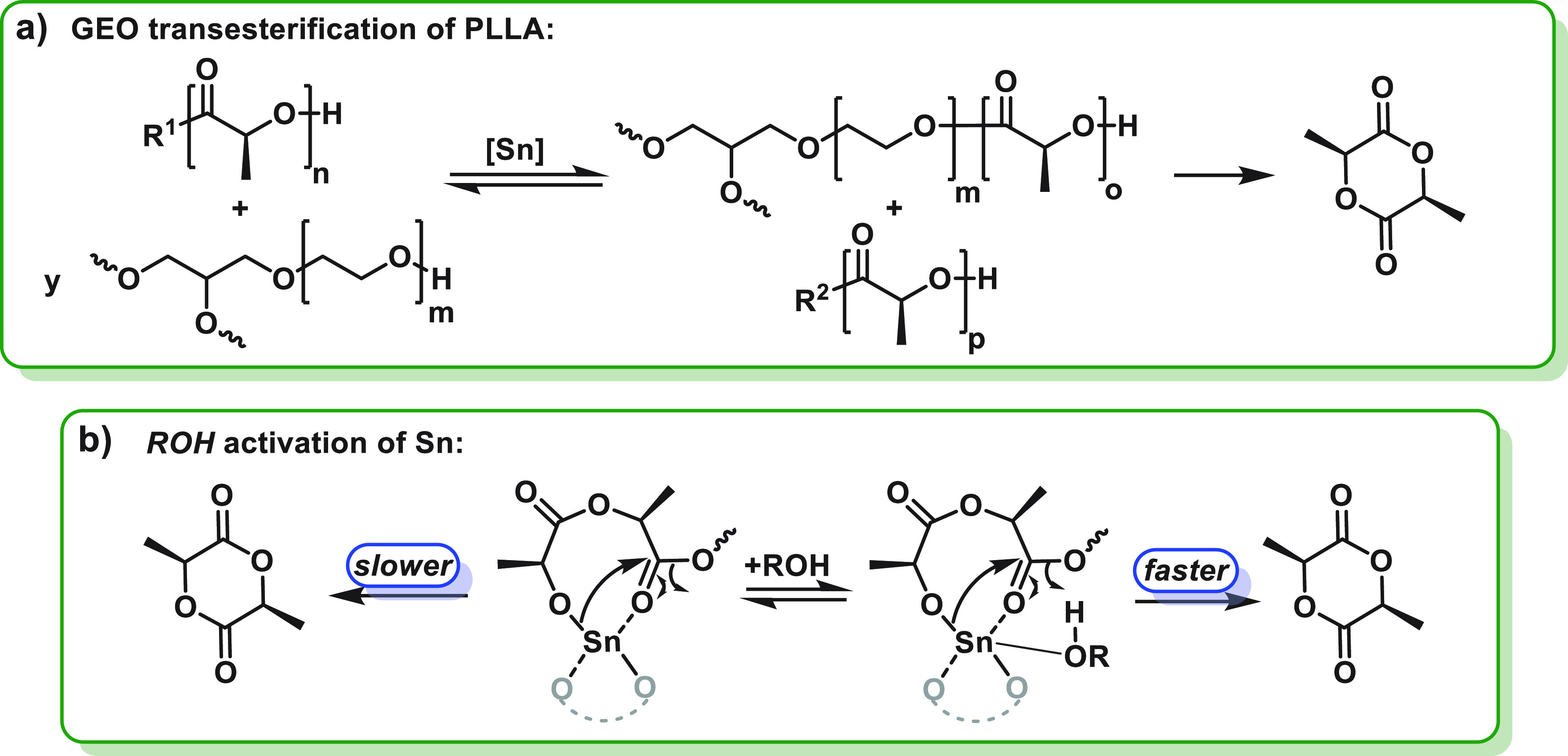
Proposed rate enhancement mechanisms for PLLA chemical recycling using Sn(Oct)_2_ and alcohol (GEO) catalyst systems. (a) The triol undergoes transesterification with the PLLA, which increases the overall PLLA-OH end-group concentration and simultaneously reduces its molar mass and viscosity. (b) The alcohol hydroxyl groups may accelerate rates by increasing the nucleophilicity of the Sn-OR chain end.

Continuing to increase the alcohol concentration resulted in the highest rates at Sn:OH = 0.25:30. At even higher hydroxyl loadings of Sn:OH = 0.25:80, there was a leveling off of the rates, possibly due to OH saturation of the catalyst (Table 2, entry 9, Table S5, Figure S63). The data are tentatively interpreted by the hydroxyl moieties, from the GEO, accelerating the depolymerization kinetics by two different mechanisms: (1) The alcohol groups reduce the overall PLLA chain length, by transesterification reactions, and increase the overall PLLA hydroxyl end-group concentration. This may help to drive equilibria toward the Sn-alkoxide catalyst, increasing its concentration and accelerating rates (Figure 5a). The transesterification simultaneously reduces the overall PLLA chain length and may reduce its viscosity, overcoming any mass-transfer limitations. (2) The alcohol groups coordinate to the Sn catalyst (Figure 5b). This increases the nucleophilicity of the chain-end Sn-alkoxide, lowering the barrier to backbiting and thereby increasing rates of l-LA formation. In the future, further investigations to delineate the multiple positive influences of alcohols on the depolymerization rates are recommended.

The chemical recycling using Sn(Oct)_2_/GEO catalyst systems resulted in equivalently high-purity l-LA without any evidence of epimerization (Figures S64–S67). In addition, GEO did not contaminate the l-LA, as determined by analyses of purity using NMR spectroscopy and GC-MS (Figure S68). This finding is significant, since equivalent chemical recycling experiments using 4-methylbenzyl alcohol, 1,4-benzenedimethanol, 1,1,1-trishydroxymethylpropane, or oligomeric alcohol end-capped PEG samples (*M*_n_ = 200 and 800 g/mol) all showed significant alcohol impurities in the isolated l-LA (Figures S69–S71). Therefore, using Sn(Oct)_2_ with GEO results in the best catalyst system since the alcohol has low volatility and high temperature stability and delivers the highest purity l-LA.

The Sn(Oct)_2_/GEO catalyst system also significantly outperforms all other PLLA depolymerization catalysts reported so far in the literature.^24−26^ While comparisons of catalysts can be complicated by the different conditions used to test them, the key targets in chemical recycling should be to deliver processes that operate at the lowest temperatures while achieving high rates and l-LA selectivity. At 160 °C, the current catalyst exhibits a 160× higher rate, even at 500× lower loading, than when it was applied in solution (Table 2, entry 10).^25^ When applied at 160× lower loading, the Sn(Oct)_2_/GEO catalyst system is much more active than the Zn(OAc)_2_ which is essentially inactive at 160 °C (Table 2, entry 12).^24^ Moreover, compared with the excellent recent report from Byers and co-workers, using a Zn(II)/PEG catalyst system, it shows 3000× higher activity at 500× lower loading (Table 2, entry 14).^26^ These results do not diminish the prior investigations, which had different objectives. This work does not seek to evaluate the optimum processes for PLLA recycling where considerations of solvent selection, recycling, overall process energy, and efficiency would need to be compared. Rather the solid-state recycling approach may be worth further exploration in terms of applying a simple, commercial catalyst system in polyester chemical recycling to monomer. In the future, this method and catalyst system should be prioritized for testing with other polymers, including for the chemical recycling of poly(ε-caprolactone),^50^ poly(hydroxy alkanoates),^6,51,52^ and other specialized related polymers.^7,35,53−57^ Some of these polymers were already investigated in catalyzed depolymerizations, but processes were conducted using very high (1–10 mol %) catalyst loadings and typically occurred rather slowly.

**Figure 6:**
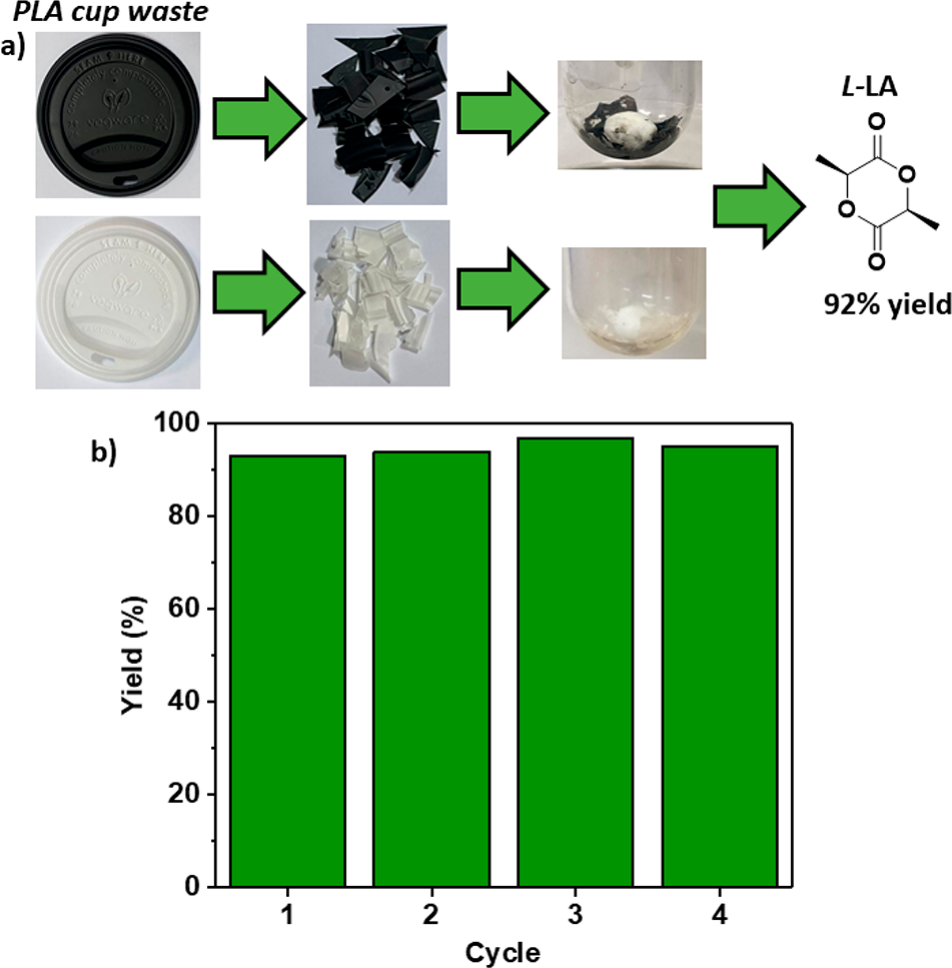
Data showing CRM of waste PLLA coffee cup lids and demonstrating catalyst system recycling over four “batches” of PLLA. (a) Chemical recycling of PLLA coffee cup lids to l-LA. (b) Plot of l-LA yields over four different CRM using Goodfellows PLLA and the same Sn(Oct)_2_/GEO catalyst ([Sn(Oct)_2_]_0_:[OH_GEO_]_0_:[PLLA]_0_ = 0.25:20:1000, 160 °C). Cycle 1 yield = 92%, cycle 2 yield = 94%, cycle 3 yield = 97%, and cycle 4 yield = 95%. Purity of l-LA > 99% across all cycles.

Since the catalyst system was highly effective using higher molar mass commercial PLLA samples, i.e., materials with chain lengths exceeding entanglement molar masses, the next task was to investigate its applicability for the chemical recycling of “real-world” PLLA-waste plastics (Figure 6a). Two different grades of PLLA coffee cup lids (Vegware, white and black) were removed from the waste bins in the cafeteria in the Department of Chemistry, University of Oxford. The lids were cleaned with water (to remove coffee residues) and dried before being cut into ca. 1–2 cm plastic chunks. The chunks were suspended in DCM at [Sn(Oct)_2_]_0_:[OH_GEO_]_0_:[PLA-cup]_0_ loadings of 0.25:40:4000. The lids were not completely soluble, since they contain inorganic fillers. Nonetheless, the suspensions were cast into films, dried, and subjected to the depolymerization conditions (160 °C and 0.05 mbar). In both cases, the chemical recycling was surprisingly successful, allowing isolation of pure l-LA in 92% yield (based on sample PLLA content of ca. 88%). Even using a black coffee cup lid, the l-LA isolated was high purity and white (Figure S72). One potential advantage of the solid-state PLLA chemical recycling process would be if the catalyst system could itself be recycled using different waste PLLA batches. As such, the Sn(Oct)_2_ catalyst/GEO catalyst system was applied in Goodfellows PLLA chemical recycling, allowing for isolation of pure l-LA in 93% yield (Figure 6b). After the reaction, a new batch of PLLA was added and the catalyst system “recycled” to depolymerize it to l-LA. In this second run, the catalyst delivered l-LA with equivalent 94% yield and purity. The catalyst system was recycled over four different batches of PLLA and showed no compromise to its productivity, constantly delivering the highest purity and high yields of l-LA (>90% yield). It was noted that after the fourth CRM cycle, the effective Sn(Oct)_2_ loading was just ∼0.006 mol %. To demonstrate the potential to apply it at such low loadings during a single depolymerization, a further larger-scale chemical recycling was performed using 10 g of PLLA and [Sn(Oct)_2_]_0_:[OH_GEO_]_0_:[PLLA]_0_ = 0.0625:20:1000. As expected the recycling rate was slower, with complete depolymerization occurring over 24 h, rather than <6 h, likely due to the unoptimized conditions for larger-scale reactions (Figure S73). Nonetheless, the CRM was successful at this low loading, and the l-LA was isolated in both excellent yield (9.15 g, 92% yield) and very high purity. Finally, the isolated l-LA may be (re)polymerized to form PLLA of high molar mass (*M*_n,SEC_ ∼ 250,000 g mol^–1^) with comparable thermal properties to the virgin material (Figures S74–S78).

## Conclusions

A commercial tin(II) and alcohol catalyst system showed very high activity and selectivity in the solid-state chemical recycling of poly(l-lactic acid), PLLA, to lactide, l-LA. Plastic films, cast with very low catalyst loadings, were efficiently depolymerized at low temperatures (160 °C) and under nitrogen flows. The chemical recycling allowed for high isolated monomer yields and occurred without any epimerization or other side reactions. The catalyst system was applied at loadings as low as 0.006 mol % Sn(II). It was effective using higher molar mass commercial PLLA samples and showed activities of ∼3000 h^–1^. These rates are orders of magnitude (∼1000×) higher than any previously reported chemical recycling catalysts, and the catalyst is applied at significantly (500×) lower loading. The successful chemical recycling of waste PLLA coffee cup lids delivered a 92% isolated yield of l-lactide, in high purity. The catalyst system is expected to show excellent performances in chemical recycling of other bioderived plastics and should be tested using poly(hydroxyalkanoates), polyesters, and polycarbonates.

## Supporting Information Available

Experimental methods, characterization, and polymer/monomer purity evaluation data (PDF)
