# Supplementary material for: Chemical Recycling of Commercial Poly(l-lactic acid) to l-Lactide Using a High-Performance Sn(II)/Alcohol Catalyst System
Source: J Am Chem Soc. 2023 Sep 1;145(36):19840–8. doi: 10.1021/jacs.3c05863 (PMC10510327; doi:10.1021/jacs.3c05863)
Supplement: Supplementary file 1 — ja3c05863_si_001.pdf [file ja3c05863_si_001.pdf]

Supporting information: Chemical Recycling of Commercial Poly(*L*-lactic acid) to *L*-Lactide using a High Performance Sn(II)/Alcohol Catalyst System

Thomas M. McGuire,[a] Antoine Buchard*[b] and Charlotte K. Williams*[a]

[a] Department of Chemistry, Chemistry Research Laboratory, University of Oxford, 12 Mansfield Rd, Oxford, OX1 3TA, U.K

[b] Department of Chemistry, Institute for Sustainability, University of Bath, , Claverton Down, Bath BA2 7AY, U.K.

Corresponding Authors

Charlotte K. Williams: Email: charlotte.williams@chem.ox.ac.uk

Antoine Buchard: Email: a.buchard@bath.ac.uk

Supporting information: Chemical Recycling of Commercial Poly(lactic acid) to *L*-Lactide using a High Performance Sn(II)/Alcohol Catalyst System 1

1. Materials 8

2. Methods 9

2.1 Solvent-Cast Method for Depolymerisation of PLLA in TGA 9

2.2 TGA Error analysis 10

2.2 Depolymerization of PLLA to determine Lactide Selectivity 10

2.3 Depolymerization of PLLA Vegware™ Coffee Cup Lid 11

2.4 Catalyst recycling study using Goodfellows™ PLLA 11

2.4 Depolymerization of PLLA at Larger-scale 11

2.5 Repolymerization of Recycled Lactide 12

3.0 Additional information 13

Figure S1. ^{1}H NMR spectrum (400 MHz, $CDCl_3$, 298 K) of commercial PLLA (5%-meso, see Fig. S2) used for study, δ 5.09 (q, *J* = 7.1 Hz, 2H, HCMe) and δ 1.51 (d, *J* = 7.2 Hz, 6H, HCMe) 13

Figure S2. Homonuclear decoupled ^{1}H NMR spectrum ($CDCl_3$, 298K) of commercial PLLA used for study (normalized integrals of deconvoluted resonances: iis/ssi = 3%, iii = 95%, isi = 2%, consistent with approx. 5% D-lactic acid repeat units in PLLA sample) 13

Figure S3. SEC ($CHCl_3$) of commercial PLLA used for study ($M_{n,SEC}$ of 60,000 g mol^{-1} correction factor of 0.58 applied [2]) 14

Figure S4. DSC thermogram of commercial PLLA used for study 14

Figure S5. TGA thermogram of commercial PLLA used for study 15

Figure S6. ^{31}P NMR (162 MHz, $CDCl_3$ spectroscopy) showing integral of internal BPA standard against PLA-OH end-groups. Integrals given in units of 1 x 10^7 mmols 15

Table S2. Data for PLLA depolymerization using various metal salts 17

Figure S7. TGA thermograms showing repeat, independent experiments monitoring PLLA mass loss (black lines) and linear-fit of the mass loss trace (red-dashed lines) against time in $Sn(Oct)_2$:PLLA mixtures (1:1000, Table 1, entry 1). The TGAs were run with an isotherm at 160 °C until >95% mass loss 17

Figure S8. TGA thermograms showing repeat, independent experiments monitoring PLLA mass loss (black lines) and linear-fit of the mass loss trace (red-dashed lines) against time in $Zn(Oct)_2$:PLLA mixtures (1:1000, Table 1, entry 2). The TGAs were run with an isotherm at 160 °C for 5 h 18

Figure S9. TGA thermograms showing repeat, independent experiments monitoring PLLA mass loss (black lines) and linear-fit of the mass loss trace (red-dashed lines) against time in $Ca(Oct)_2$:PLLA mixtures (1:1000, Table 1, entry 3). The TGAs were run with an isotherm at 160 °C for 5 h 18

Figure S10. TGA thermograms showing repeat, independent experiments monitoring PLLA mass loss (black lines) and linear-fit of the mass loss trace (red-dashed lines) against time in $SnCl_2$:PLLA mixtures (1:1000, Table S2, entry 1). The TGAs were run with an isotherm at 160 °C for 5 h ... 19

Figure S11. TGA thermograms showing repeat, independent experiments monitoring PLLA mass loss (black lines) and linear-fit of the mass loss trace (red-dashed lines) against time in $Zn(OAc)_2$:PLLA mixtures (1:1000, Table S2, entry 2). The TGAs were run with an isotherm at 160 °C for 5 h 19

Figure S12. TGA thermograms showing repeat, independent experiments monitoring PLLA mass loss (black lines) and linear-fit of the mass loss trace (red-dashed lines) against time in $ZnCl_2$:PLLA mixtures (1:1000, Table S2, entry 3). The TGAs were run with an isotherm at 160 °C for 5 h ... 20

Figure S13. TGA thermograms showing repeat, independent experiments monitoring PLLA mass loss (black lines) and linear-fit of the mass loss trace (red-dashed lines) against time in ZnI_2:PLLA mixtures (1:1000, Table S2, entry 4). The TGAs were run with an isotherm at 160 °C for 5 h ... 21

Figure S14. TGA thermograms showing repeat, independent experiments monitoring PLLA mass loss (black lines) and linear-fit of the mass loss trace (red-dashed lines) against time in $Ba(Oct)_2$:PLLA mixtures (1:1000, Table S2, entry 5). The TGAs were run with an isotherm at 160 °C for 5 h ... 21

Figure S15. TGA thermograms showing repeat, independent experiments monitoring PLLA mass loss (black lines) and linear-fit of the mass loss trace (red-dashed lines) against time in $Fe(OAc)_2$:PLLA mixtures (1:1000, Table S2, entry 6). The TGAs were run with an isotherm at 160 °C for 5 h ... 22

Figure S16. TGA thermograms showing repeat, independent experiments monitoring PLLA mass loss (black lines) and linear-fit of the mass loss trace (red-dashed lines) against time in $FeCl_2$:PLLA mixtures (1:1000, Table S2, entry 7). The TGAs were run with an isotherm at 160 °C for 5 h ... 22

Figure S17. TGA thermograms showing repeat, independent experiments monitoring PLLA mass loss (black lines) and linear-fit of the mass loss trace (red-dashed lines) against time in $Mg(HMDS)_2$:PLLA mixtures (1:1000, Table S2, entry 8). The TGAs were run with an isotherm at 160 °C for 5 h ... 23

Figure S18. TGA thermograms showing repeat, independent experiments monitoring PLLA mass loss (black lines) and linear-fit of the mass loss trace (red-dashed lines) against time in $Bi(Oct)_3$:PLLA mixtures (1:1000, Table S2, entry 9). The TGAs were run with an isotherm at 160 °C for 5 h ... 23

Figure S19. TGA thermograms showing repeat, independent experiments monitoring PLLA mass loss (black lines) and linear-fit of the mass loss trace (red-dashed lines) against time in $Bi(OAc)_3$:PLLA mixtures (1:1000, Table S2, entry 10). The TGAs were run with an isotherm at 160 °C for 5 h ... 24

Figure S20. TGA thermograms showing repeat, independent experiments monitoring PLLA mass loss (black lines) and linear-fit of the mass loss trace (red-dashed lines) against time in $Ti(Oct)_4$:PLLA mixtures (1:1000, Table S2, entry 11). The TGAs were run with an isotherm at 160 °C for 5 h ... 24

Figure S21. TGA thermograms showing repeat, independent experiments monitoring PLLA mass loss (black lines) and linear-fit of the mass loss trace (red-dashed lines) against time in $Zr(Oct)_4$:PLLA mixtures (1:1000, Table S2, entry 12). The TGAs were run with an isotherm at 160 °C for 5 h ... 25

Figure S22. TGA-FTIR gas-phase analysis of PLLA depolymerization mixture catalysed by $Sn(Oct)_2$ ($[Sn(Oct)_2]_0$:$[PLLA]_0$ = 1:1000, 160 °C, black) and an L-lactide standard (blue). ... 25

Figure S23. (left) Setup used for depolymerization-sublimation of PLLA to LA. (right) Lactide collected on a cold finger ... 26

Figure S24. ^{1}H NMR (400 MHz, $CDCl_3$, 298K) spectrum of LA isolated from depolymerization of PLLA ($[Sn(Oct)_2]_0$:$[PLLA]_0$ 1:1000, 160 °C, Table 1, entry 1) δ 5.05 (q, *J* = 6.7 Hz, 2H, L,L + D,L HCMe), 1.66 (d, *J* = 7.1 Hz, *trace (5% meso)*, D,L HCMe)δ 1.60 (d, *J* = 6.7 Hz, 6H, L,L HCMe). ... 27

Figure S25. GC of LA isolated from depolymerization of PLLA ($[Sn(Oct)_2]_0$:$[PLLA]_0$ 1:1000, 160 °C, Table 1, entry 1, *m*-LA = 5%, *L*-LA = 95%) ... 28

Figure S26. ^{1}H NMR (400 MHz, $CDCl_3$, 298K) spectrum of L-LA isolated from depolymerization of 100%-PLLA ($[Sn(Oct)_2]_0$:$[PLLA]_0$ 1:1000, 160 °C) δ 5.04 (q, *J* = 6.7 Hz, 2H, L,L-LA, HCMe), δ 1.67 (d, *J* = 6.7 Hz, 6H, L,L-LA HCMe). ... 29

Figure S27. GC of L-LA isolated from depolymerization of 100%-PLLA ($[Sn(Oct)_2]_0$:$[PLLA]_0$ 1:1000, 160 °C, *L*-LA = 100%) ... 30

Table S3. Depolymerization of PLLA at various reaction temperatures ... 30

Figure S28. TGA thermograms showing repeat, independent experiments monitoring PLLA mass loss (black lines) and linear-fit of the mass loss trace (red-dashed lines) against time in

$Sn(Oct)_2$:PLLA mixtures (1:1000, Table S3, entry 2). The TGAs were run with an isotherm at 165 °C until >95% mass loss 31

Figure S29. TGA thermograms showing repeat, independent experiments monitoring PLLA mass loss (black lines) and linear-fit of the mass loss trace (red-dashed lines) against time in $Sn(Oct)_2$:PLLA mixtures (1:1000, Table 1, entry 4 and Table S3, entry 3). The TGAs were run with an isotherm at 170 °C until >95% mass loss 32

Figure S30. TGA thermograms showing repeat, independent experiments monitoring PLLA mass loss (black lines) and linear-fit of the mass loss trace (red-dashed lines) against time in $Sn(Oct)_2$:PLLA mixtures (1:1000, Table S3, entry 4). The TGAs were run with an isotherm at 175 °C until >95% mass loss 32

Figure S31. TGA thermograms showing repeat, independent experiments monitoring PLLA mass loss (black lines) and linear-fit of the mass loss trace (red-dashed lines) against time in $Sn(Oct)_2$:PLLA mixtures (1:1000, Table 1, entry 5 and Table S3, entry 5). The TGAs were run with an isotherm at 180 °C until >95% mass loss 33

Figure S32. ^{1}H NMR (400 MHz, $CDCl_3$, 298K) spectrum of LA isolated from depolymerization of PLLA ($[Sn(Oct)_2]_0$:$[PLLA]_0$ 1:1000, 180 °C, Table 1, entry 5) δ 5.05 (q, *J* = 6.7 Hz, 2H, L,L + D,L HCMe), 1.66 (d, *J* = 7.1 Hz, *trace (6% meso)*, D,L HCMe)δ 1.60 (d, *J* = 6.7 Hz, 6H, L,L HCMe). 34

Figure S33. GC of LA isolated from depolymerization of PLLA ($[Sn(Oct)_2]_0$:$[PLLA]_0$ 1:1000, 180 °C, Table 1, entry 5, *m*-LA = 5%, *L*-LA = 95%) 35

Table S4. Depolymerization of PLLA at various $Sn(Oct)_2$ loadings 36

Figure S34. TGA thermograms showing repeat, independent experiments monitoring PLLA mass loss (black lines) and linear-fit of the mass loss trace (red-dashed lines) against time in $Sn(Oct)_2$:PLLA mixtures (0.5: 1000, Table 1, entry 6 and Table S4, entry 2). The TGAs were run with an isotherm at 160 °C until >95% mass loss 37

Figure S35. TGA thermograms showing repeat, independent experiments monitoring PLLA mass loss (black lines) and linear-fit of the mass loss trace (red-dashed lines) against time in $Sn(Oct)_2$:PLLA mixtures (0.33: 1000, Table 1, entry 7 and Table S4, entry 3). The TGAs were run with an isotherm at 160 °C for 5 h 37

Figure S36. TGA thermograms showing repeat, independent experiments monitoring PLLA mass loss (black lines) and linear-fit of the mass loss trace (red-dashed lines) against time in $Sn(Oct)_2$:PLLA mixtures (0.25: 1000, Table 1, entry 8 and Table S4, entry 4). The TGAs were run with an isotherm at 160 °C for 5 h 38

Figure S37. TGA thermograms showing repeat, independent experiments monitoring PLLA mass loss (black lines) and linear-fit of the mass loss trace (red-dashed lines) against time in $Sn(Oct)_2$:PLLA mixtures (0.2: 1000, Table 1, entry 9 and Table S4, entry 5). The TGAs were run with an isotherm at 160 °C for 5 h 38

Figure S38. TGA thermograms showing repeat, independent experiments monitoring PLLA mass loss (black lines) and linear-fit of the mass loss trace (red-dashed lines) against time in $Sn(Oct)_2$:PLLA mixtures (0.167: 1000, Table S4, entry 6). The TGAs were run with an isotherm at 160 °C for 5 h 39

Figure S39. TGA thermograms showing repeat, independent experiments monitoring PLLA mass loss (black lines) and linear-fit of the mass loss trace (red-dashed lines) against time in $Sn(Oct)_2$:PLLA mixtures (0.125: 1000 Table S4, entry 7). The TGAs were run with an isotherm at 160 °C for 5 h 39

Figure S40. TGA thermograms showing repeat, independent experiments monitoring PLLA mass loss (black lines) and linear-fit of the mass loss trace (red-dashed lines) against time in $Sn(Oct)_2$:PLLA mixtures (0.1:1000, Table S4, entry 8). The TGAs were run with an isotherm at 160 °C for 5 h 40

Figure S41. ^{1}H NMR (400 MHz, $CDCl_3$, 298K) spectrum of LA isolated from depolymerization of PLLA ($[Sn(Oct)_2]_0$:$[PLLA]_0$ 0.25: 1000, 160 °C, Table 1, entry 8) δ 5.05 (q, *J* = 6.7 Hz, 2H, L,L + D,L HCMe), 1.66 (d, *J* = 7.1 Hz, *trace (5% meso)*, D,L HCMe)δ 1.60 (d, *J* = 6.7 Hz, 6H, L,L HCMe). 41

Figure S42. GC of LA isolated from depolymerization of PLLA ($[Sn(Oct)_2]_0$:$[PLLA]_0$ 0.25: 1000, 160 °C, Table 1, entry 8, *m*-LA peak 5%, *L*-LA peak 95%) 42

Figure S43. TGA thermograms showing repeat, independent experiments monitoring PLLA mass loss (black lines) and linear-fit of the mass loss trace (red-dashed lines) against time in $Sn(O^nBu)_2$:PLLA mixtures (1:1000, Table 1, entry 10). The TGAs were run with an isotherm at 160 °C until >95% mass loss 43

Figure S44. ^1H NMR (400 MHz, $CDCl_3$, 298K) spectrum of LA isolated from depolymerization of PLLA ($[Sn(O^nBu)_2]_0$:$[PLLA]_0$ 1:1000, 160 °C, Table 1, entry 10) δ 5.05 (q, *J* = 6.7 Hz, 2H, L,L + D,L HCMe), 1.66 (d, *J* = 7.1 Hz, *trace (4% meso)*, D,L HCMe) δ 1.60 (d, *J* = 6.7 Hz, 6H, L,L HCMe). 44

Figure S45. GC of LA isolated from depolymerization of PLLA ($[Sn(Oct)_2]_0$:$[PLLA]_0$ 1:1000, 160 °C, Table 1, entry 10, *m*-LA = 5%, *L*-LA = 95%) 45

Figure S46. TGA thermograms showing mass loss against time in $Sn(Oct)_2$:PLLA-OH (black) and $Sn(Oct)_2$:PLLA-OAc mixtures (blue) (1:1000) at 160 °C 45

Figure S47. Structures of CTAs used in this study. 46

Figure S48. TGA thermograms showing mass loss of MBA (purple), BDM(black), THMP (green), DPE (red), PEEO 300 (cyan), PEEO 800 (orange) and GEO 1000 (navy) at 160 °C under N_2 flow 46

Figure S49. ^1H NMR spectra of GEO-PLLA transesterification reactions ($[Sn(Oct)_2]_0$:$[OH_{GEO}]_0$:$[PLLA]_0$ = 1: 20: 1000, 160 °C, neat) at time = 0 and time = 10 mins. 47

Figure S50. ^1H-^{13}C HSQC NMR spectrum ($CDCl_3$) of GEO-PLLA transesterification reactions ($[Sn(Oct)_2]_0$:$[OH_{GEO}]_0$:$[PLLA]_0$ = 1: 20: 1000, 160 °C, neat) at time =10 mins. Insert highlights the ^1H-^{13}C single-bond correlation of the GEO-junction unit 48

Figure S51. ^1H-^{13}C HMBC NMR spectrum ($CDCl_3$) of GEO-PLLA transesterification reactions ($[Sn(Oct)_2]_0$:$[OH_{GEO}]_0$:$[PLLA]_0$ = 1: 20: 1000, 160 °C, neat) at time =10 mins. Insert highlights the ^1H-^{13}C three-bond correlation between of the GEO-junction unit and the ester carbonyl of PLLA. 49

Figure S52. TGA thermograms showing repeat, independent experiments monitoring PLLA mass loss (black lines) and linear-fit of the mass loss trace (red-dashed lines) against time in $Sn(Oct)_2$:GEO:PLLA mixtures ($[Sn(Oct)_2]_0$:$[OH_{GEO}]_0$:$[PLLA]_0$ = 1: 20: 1000, Table 2, entry 1). The TGAs were run with an isotherm at 160 °C until >95% mass loss 50

Figure S53. TGA thermograms showing repeat, independent experiments monitoring PLLA mass loss (black lines) and linear-fit of the mass loss trace (red-dashed lines) against time in $Sn(Oct)_2$:GEO:PLLA mixtures ($[Sn(Oct)_2]_0$:$[OH_{GEO}]_0$:$[PLLA]_0$ = 0.5 :20 :1000, Table 2, entry 2). The TGAs were run with an isotherm at 160 °C until >95% mass loss 50

Figure S54. TGA thermograms showing repeat, independent experiments monitoring PLLA mass loss (black lines) and linear-fit of the mass loss trace (red-dashed lines) against time in $Sn(Oct)_2$:GEO:PLLA mixtures ($[Sn(Oct)_2]_0$:$[OH_{GEO}]_0$:$[PLLA]_0$ = 0.25 :20 :1000, Table 2, entry 3). The TGAs were run with an isotherm at 160 °C until >95% mass loss 51

Figure S55. TGA thermograms showing repeat, independent experiments monitoring PLLA mass loss (black lines) and linear-fit of the mass loss trace (red-dashed lines) against time in $Sn(Oct)_2$:GEO:PLLA mixtures (($[Sn(Oct)_2]_0$:$[OH_{GEO}]_0$:$[PLLA]_0$ = 0.2 :20 :1000, Table 2, entry 4). The TGAs were run with an isotherm at 160 °C until >95% mass loss 51

Figure S56. TGA thermograms showing repeat, independent experiments monitoring PLLA mass loss (black lines) and linear-fit of the mass loss trace (red-dashed lines) against time in $Sn(Oct)_2$:GEO:PLLA mixtures ($[Sn(Oct)_2]_0$:$[OH_{GEO}]_0$:$[PLLA]_0$= 0.125 :20 :1000, Table 2, entry 5). The TGAs were run with an isotherm at 160 °C until >95% mass loss 52

Figure S57. TGA thermograms showing repeat, independent experiments monitoring PLLA mass loss (black lines) and linear-fit of the mass loss trace (red-dashed lines) against time in $Sn(Oct)_2$:GEO:PLLA mixtures ($[Sn(Oct)_2]_0$:$[OH_{GEO}]_0$:$[PLLA]_0$ = 0.1: 20: 1000, Table 2, entry 6). The TGAs were run with an isotherm at 160 °C until >95% mass loss 53

Table S5. Data for $Sn(Oct)_2$-catalyzed PLLA depolymerization in the presence of GEO at fixed $[Sn(Oct)_2]_0$ 54

Figure S58. TGA thermograms showing repeat, independent experiments monitoring PLLA mass loss (black lines) and linear-fit of the mass loss trace (red-dashed lines) against time in $Sn(Oct)_2$:GEO:PLLA mixtures (($[Sn(Oct)_2]_0$:$[OH_{GEO}]_0$:$[PLLA]_0$ = 0.25: 10: 1000, Table 2, entry 7 and Table S5, entry 1). The TGAs were run with an isotherm at 160 °C until >95% mass loss.. 55

Figure S59. TGA thermograms showing repeat, independent experiments monitoring PLLA mass loss (black lines) and linear-fit of the mass loss trace (red-dashed lines) against time in $Sn(Oct)_2$:GEO:PLLA mixtures ($[Sn(Oct)_2]_0$:$[OH_{GEO}]_0$:$[PLLA]_0$ = 0.25 :30 :1000, Table 2, entry 8 and Table S5, entry 3). The TGAs were run with an isotherm at 160 °C until >95% mass loss......... 55

Figure S60. TGA thermograms showing repeat, independent experiments monitoring PLLA mass loss (black lines) and linear-fit of the mass loss trace (red-dashed lines) against time in $Sn(Oct)_2$:GEO:PLLA mixtures ($[Sn(Oct)_2]_0$:$[OH_{GEO}]_0$:$[PLLA]_0$ = 0.25: 40: 1000, Table S5, entry 4). The TGAs were run with an isotherm at 160 °C until >95% mass loss 56

Figure S61. TGA thermograms showing repeat, independent experiments monitoring PLLA mass loss (black lines) and linear-fit of the mass loss trace (red-dashed lines) against time in $Sn(Oct)_2$:GEO:PLLA mixtures ($[Sn(Oct)_2]_0$:$[OH_{GEO}]_0$:$[PLLA]_0$ = 0.25 :50 :1000, Table S5, entry 5). The TGAs were run with an isotherm at 160 °C until >95% mass loss 56

Figure S62. TGA thermograms showing repeat, independent experiments monitoring PLLA mass loss (black lines) and linear-fit of the mass loss trace (red-dashed lines) against time in $Sn(Oct)_2$:GEO:PLLA mixtures ($[Sn(Oct)_2]_0$:$[OH_{GEO}]_0$:$[PLLA]_0$ = 0.25: 80:1000, Table 2, entry 9 and Table S5, entry 6). The TGAs were run with an isotherm at 160 °C until >95% mass loss......... 57

Figure S63. k_{obs}^{GEO} *vs* $[OH_{GEO}]_0/[PLLA]_0$. Depolymerizations conducted at 160 °C with varied $[OH_{GEO}]_0$ and $[Sn(Oct)_2]_0$:$[PLLA]_0$ = 0.25:1000 57

Figure S64. ^1H NMR ($CDCl_3$, 298K) spectrum of LA isolated from depolymerization of PLLA from mixtures of $Sn(Oct)_2$, GEO and PLLA ($[Sn(Oct)_2]_0$:$[OH_{GEO}]_0$:$[PLLA]_0$ = 0.1: 20:1000,160 °C, Table 2, entry 6) 58

Figure S65. GC of LA isolated from depolymerization of PLLA from mixtures of $Sn(Oct)_2$, GEO and PLLA ($[Sn(Oct)_2]_0$:$[OH_{GEO}]_0$:$[PLLA]_0$ = 0.1: 20:1000, Table 2, entry 6, 160 °C, *m*-LA = 5%, *L*-LA = 95%).. 59

Figure S66. ^1H NMR ($CDCl_3$, 298K) spectrum of LA isolated from depolymerization of PLLA from mixtures of $Sn(Oct)_2$, GEO and 100% PLLA ($[Sn(Oct)_2]_0$:$[OH_{GEO}]_0$:$[PLLA]_0$ = 0.1: 20:1000,160 °C) 60

Figure S67. GC of LA isolated from depolymerization of PLLA from mixtures of $Sn(Oct)_2$, GEO and 100%-PLLA ($[Sn(Oct)_2]_0$:$[OH_{GEO}]_0$:$[PLLA]_0$ = 0.1: 20:1000, 160 °C, *L*-LA = 100%) 61

Figure S68. ^1H NMR (400 MHz, $CDCl_3$, 298K) spectrum of LA isolated from depolymerization of PLLA from mixtures of $Sn(Oct)_2$, GEO and PLLA ($[Sn(Oct)_2]_0$:$[OH_{GEO}]_0$: $[PLLA]_0$, 0.1: 20: 1000, 160 °C). GEO or GEO-like resonance not detected... 62

Figure S69. ^1H NMR (400 MHz, $CDCl_3$, 298K) spectrum of LA isolated from depolymerization of PLLA from mixtures of $Sn(Oct)_2$, MBA and PLLA ($[Sn(Oct)_2]_0$:$[OH_{MBA}]_0$: $[PLLA]_0$ 1: 20: 1000, 160 °C). MBA-like contaminant highlighted in insert.. 63

Figure S70. ^1H NMR (400 MHz, $CDCl_3$, 298K) spectrum of LA isolated from depolymerization of PLLA from mixtures of $Sn(Oct)_2$, THMP and PLLA mixtures ($[Sn(Oct)_2]_0$:$[OH_{THMP}]_0$: $[PLLA]_0$ 1: 20: 1000, 160 °C). THMP-like contaminant highlighted in insert 64

Figure S71. ^1H NMR (400 MHz, $CDCl_3$, 298K) spectrum of LA isolated from depolymerization of PLLA from mixtures of $Sn(Oct)_2$, PEEO and PLLA ($[Sn(Oct)_2]_0$:$[OH_{PEEO}]_0$: $[PLLA]_0$ 1: 20: 1000, 160 °C) PEEO-like contaminant highlighted in insert.. 65

Figure S72. Representative *L*-LA isolated from depolymerization of PLLA using $Sn(Oct)_2$ and GEO catalyst 66

Figure S73. Sublimation kit for depolymerization of 10.0 g of PLLA using $Sn(Oct)_2$ and GEO ($[Sn(Oct)_2]_0$:$[OH_{GEO}]_0$: $[PLLA]_0$ 0.0625: 20: 10000, 160 °C)... 66

Figure S74. ^1H NMR spectrum (400 MHz, $CDCl_3$, 298 K) of commercial PLLA (5%-meso, see Fig. S2) used for study, δ 5.09 (q, *J* = 7.1 Hz, 2H, HCMe) and δ 1.51 (d, *J* = 7.2 Hz, 6H, HCMe)..... 67

Figure S75. SEC traces for virgin (blue) and recycled PLLA (black). Recycled PLLA molar mass 230,000 g mol^{-1} (correction factor of 0.58 applied) and $Đ_M$ =1.34. 67

Figure S76. DSC thermogram of virgin PLLA and recycled PLLA. 68
Figure S77. TGA thermogram of of virgin PLLA and recycled PLLA 68
Figure S78. Photograph of recycled PLLA 69
4.0 References 69

1. Materials

All experiments were carried out under N_2 using standard Schlenk/glovebox techniques unless otherwise stated. PLLA was purchased from GoodFellows and dried at room temperature under high vacuum (1 x10^{-2}, mbar) prior to use. Where highlighted, waste PLLA was sourced from the Univeristy of Oxford's Department of Chemistry cafeteria disposal units. The waste PLLA was manufactured by vegware. Tin(II) 2-ethylhexanoate ($Sn(Oct)_2$) was purchased from Sigma-Aldrich and used as received. Glycerol ethoxylate (GEO, M_n ~1000 g mol^{-1}) was purchased from Sigma-Aldrich, dried at 120 °C under high vacuum (1 x10^{-2}, mbar) and stored over 3 Å sieves for 2 days prior to use. Pentaerythritol ethoxylate (PE/EO 15/4, M_n ~800 g mol^{-1}) was purchased from Sigma-Aldrich, dried at 120 °C under high vacuum (1 x10^{-2}, mbar) and stored over 3 Å sieves for 2 days prior to use. 4-Methylbenzyl alcohol, 1,4-benzenedimethanol was purchased from Sigma-Aldrich and recrystallised from anhydrous diethyl ether prior to use. 1,1,1-Tris(hydoxymethyl)propane was purchased from Sigma-Aldrich and recrystallised from anhydrous THF prior to use. Anhydrous dichloromethane was purchased from Sigma-Aldrich and degassed by N_2 purge prior to use. Diethyl ether, THF and toluene were obtained from an SPS system, degassed by several freeze-pump-thaw cycles and stored over 3 Å molecular sieves under nitrogen. Tin(II) *n*-butoxide was synthesised according to literature reports.[1] Tin(II) chloride, bismuth(III) acetate, zinc(II) chloride (anhydrous), zinc(II) iodide (anhydrous), iron(II) chloride (anhydrous), iron(II) acetate (anhydrous), calcium(II) 2-ethylhexanoate, magnesium(II) bis(hexamethyldisilazido) and barium(II) 2-ethylhexanoate were purchased from Sigma-Aldrich and used as received. Titanium(IV) 2-ethylhexanoate, zirconium(IV) 2-ethylhexanoate were purchased from Alfa Aesar and used as received. Zinc(II) 2-ethylhexanoate was purchased from Fluorochem and used as received. Bismuth(III) 2-ethylhexanoate was purchased from Thermo Scientific Chemicals and used as received.

Size exclusion chromatography (SEC) was carried out on a Shimadzu LC-20AD instrument using two PSS SDV linear M columns in series, with a $CHCl_3$ eluent. Measurements were conducted at 30 °C with a flow rate of 1 mL/min. Samples were detected with a differential refractive index (RI) detector. Number-average molar mass ($M_{n,SEC}$) and dispersities ($Đ_M$ (M_w/M_n)) were calculated against a polystyrene calibration (molar mass range 500 – 1000000 g mol^{-1}) . The polymer samples were dissolved in HPLC-grade THF at a concentration of *ca* 10 mg/mL and filtered through a 0.2 µm microfilter prior to analysis

Differential scanning calorimetry (DSC) was performed using a TA discovery 25 auto. Experiments were performed under N_2 flow (50 mL/min) using aluminium TZERO pans. Samples (2–5 mg) were equilibrated at 20 °C then heated at a rate of 20 °C/min to 200 °C and held at 200 °C for 5 minutes. The sample was then cooled at a rate of 20 °C/min to –80 °C and held at –80 °C for 5 minutes. The sample was then heated at a rate of 10 °C/min to 200 °C and cooled at a rate 10 °C/min to –80 °C for 5 successive cycles. Thermal data is reported from the second heating cycle.

Thermal gravimetric analysis (TGA) were collected on a TGA5500 System (TA Instruments), equipped with the TRIOS software package. Detailed procedures are given in the protocol section.

NMR spectra were obtained using a Bruker AVIII HD nanobay NMR spectrometer. Coupling constants are given in Hertz. Selectivities were determined by ^{1}H NMR spectroscopy.

GC-MS spectra were recorded on an Agilent 7820A equipped with a HP5-MS ultra inert column (30 m length, 0.25 mm internal diameter, 0.25 µm film thickness), a 5977B single quad mass spectrometer, a liquid injection autosampler and He carrier gas. Data was processed using MassHunter software. Samples of 5 mg/mL were prepared in dichloromethane with 1µL injected. Samples were loaded on to the column in 1:100 sample:solvent splitter ratio and injection port temperature of 300 °C.The column was pressurised at 9.1 PSI, with a column flow of 1.2 mL/min and total flow of 22.12 ml/min. Following equilibration of the column oven at 40 °C for 1 minute, the temperature was ramped from 40 °C to 300 °C at a rate of 10 °C/min and held at 300 °C for 3 minutes. The MS source and quadrupole temperature was 230 °C and 150 °C, respectively.

Turnover Frequency (TOF) calculations were performed using mass loss against time plots from 0-80% mass loss of the polymer over time.

2. Methods

N.B. all PLLA concentrations are calculated as the concentration the lactic acid repeat unit. Molar mass of GEO assumed to be 1000 g mol^{-1}.

2.1 Solvent-Cast Method for Depolymerisation of PLLA in TGA

In the glovebox, stock solutions of PLLA (1.00 M, 72 mg of PLLA in 1.00 mL of DCM), Sn(Oct)$_2$ (1.00 x 10 $^{-2}$ M,12.2 mg of Sn(Oct)$_2$ in 3.00 mL of THF) and GEO (0.03 M, 33.3 mg of GEO in 1.00 mL of THF) were prepared. The PLLA stock solution (100 µL, 0.1 mmol, 1000.0 equiv.) was added to a vial containing Sn(Oct)$_2$ (10.0 µL, 1.00 x 10 $^{-4}$ mmol, 1.0 equiv.) and GEO (20.0 µL, 6.67 x 10 $^{-4}$ mmol, 6.7 equiv.). The Sn(Oct)$_2$-GEO-PLLA solution was thoroughly mixed before being dropcast (*ca* 3 drops) onto Platinum TGA crucibles. The solvent was allowed to evaporate before the crucible was loaded into the TGA for monitored solid-state depolymerization. The following TGA method template was used for the reaction:

N_2 flow of 25.0 mL min^{-1}
Jump from room temperature to 160 °C
Isotherm for 300 minutes
Ramp at 20 °C min^{-1} to 600 °C
Jump to 30 °C.

The catalyst loading, GEO loading, reaction temperature and length of the isotherm were varied as required.

To account for residual solvent loss from the polymer films, which will also be detected as a mass loss in TGA, data from the first 1.5 minutes of the run was removed from the analysis. The mass at 1.5 minutes was then taken as the polymer/catalyst initial mass and used to calculate the change in weight % of the sample.

For reactions where GEO was present, the residual mass of the GEO was accounted for as follows:

1) Take the mass at time = 1.5 minutes as the initial polymer, GEO and catalyst mass.
2) Subtract the theoretical %mass of GEO in the run:

e.g. for a loading of 0.1:6.67:1000 Sn(Oct)$_2$:GEO:PLLA:

% mass of GEO = 6.67(M_r of GEO)/[6.7(M_r of GEO) + 0.1(M_r of Sn(Oct)$_2$)+1000(M_r of PLLA repeat unit (72.06))]

= 6.67(1000)/[0.1(405.12)+6.7(1000)+1000(72.06)]

= 8.502%

Therefore mass of initial polymer/catalyst film = 91.498% of mass at 1.5 minutes.

3) Take the mass in step 2) as the initial mass for calculation of the change in mass% of the sample.

2.2 TGA Error analysis

Example error analysis:

A TGA experiment performed at 160 °C at 1:1000, $Sn(Oct)_2$:PLLA was repeated 3 times (Table S1). The TOF, k_{obs} and mass loss rate were determined for all runs and the mean TOF, k_{obs} and mass loss rate were taken.

The error in the TOF, k_{obs} and mass loss rate was taken as the standard deviation of the mean.

The %error was taken as: error/mean*100.

Table S1. Example error analysis from a depolymerization performed with $[Sn(Oct)_2]_0$:$[PLLA]_0$ 1:1000 at 160 °C

Run	k_{obs} (h^{-1})[a]	TOF (h^{-1})[b]	Mass loss rate (g g^{-1} mol^{-1})[c]
1	38.2	340	61
2	34.8	390	69
3	41.5	410	73
Average	38.4	380	67
Error	±2.2	±28	±5
% Error	±5.7%	±7.4%	±7.5%

Depolymerization experiments conducted using thin films, analyzed using TGA until >95% mass loss Catalyst loadings calculated per M_r of PLLA repeat unit (M_r = 72.06 g mol^{-1}). [a] Rate constant is the gradient of the linear fits to plots of %PLLA mass loss vs time. Average errors are determined from repeat runs, see ESI for details. [b] Activity as TOF defined as moles of lactic acid repeat unit consumed from 0-80% mass loss/moles of catalyst/time taken for 0-80% mass loss. Average errors were taken from repeat runs.[c] Mass loss rate = TOF × M_r of PLLA repeat unit (72.06)/M_r of catalyst

Experiments to determine the barrier height and order in catalyst were repeated in triplicate. The error in the TOF, k_{obs} and mass loss rate was taken as the standard deviation of the mean. The %error was taken as: error/mean*100.

All further experiments were duplicated, with TOF, k_{obs} and mass loss rate taken as the mean of the repeat runs. The error in the TOF, k_{obs} and mass loss rate was taken as the standard deviation of the mean. If the errors in these runs were below the values calculated in Table S1, the %errors from table S1 were applied to the values.

2.2 Depolymerization of PLLA to determine Lactide Selectivity

In the glovebox, stock solutions of $Sn(Oct)_2$ (1.00 x 10^{-2} M, 12.2 mg of $Sn(Oct)_2$ in 3.00 mL of THF) and GEO (0.03 M, 33.3 mg of GEO in 1.00 mL of THF) were prepared. The $Sn(Oct)_2$ (50.0 µL, 5.00 x 10^{-4} mmol, 1.0 equiv.) and GEO (400 µL, 1.33 x 10^{-2} mmol, 26.7 equiv.) stock solutions were mixed in a Schlenk before PLLA was added as a solid (144 mg, 2.00 mmol, 4000.0 equiv). The PLLA, $Sn(Oct)_2$ and GEO mixture was dissolved in a minimum amount of DCM (*approx.* 1 mL) before the solvent was removed *in-vacuuo*. A water-cooled cold-finger was connected to the Schlenk and the reaction mixture was heated to 160 °C with magnetic stirring under vacuum (0.1–1 mbar). After 5 h, a white crystalline powder was collected which was determined to be lactide (133 mg, 0.924 mmol, 92% yield, 95% *L*-LA as determined by ^{1}H NMR spectroscopy and GC-MS).

N.B. the depolymerization may also be performed without prior dissolution of the polymer/catalyst mixture

2.3 Depolymerization of PLLA Vegware^{TM} Coffee Cup Lid

Vegware PLLA (0.144 g, 2 mmol, 40000 equiv.) was cut into *ca* 1-2 cm chunks and dried under vaccum for 2 h. In the glovebox, stock solutions of $Sn(Oct)_2$ (1.00 x 10 $^{-2}$ M, 12.2 mg of $Sn(Oct)_2$ in 3.00 mL of THF) and GEO (0.03 M, 33.3 mg of GEO in 1.00 mL of THF) were prepared. The $Sn(Oct)_2$ (50.0 µL, 5.00 x 10 $^{-4}$ mmol, 1.0 equiv.) and GEO (400 µL, 1.33 x 10^{-2} mmol, 26.7 equiv.) stock solutions were mixed in a Schlenk before the vegware PLLA was added as a solid. The PLLA, $Sn(Oct)_2$ and GEO mixture was suspended in a minimum amount of DCM (*approx.* 1 mL) before the solvent was removed *in-vacuuo*. A water-cooled cold-finger was connected to the Schlenk and the reaction mixture was heated to 160 °C with magnetic stirring under vacuum (0.1–1 mbar). After 5 h, a white crystalline powder was collected which was determined to be lactide (117 mg, 0.810 mmol, 92% yield based on 12 wt% inorganic filler content, 98% *L*-LA as determined by ^{1}H NMR spectroscopy and GC-MS).

2.4 Catalyst recycling study using Goodfellows^{TM} PLLA

In the glovebox, stock solutions of $Sn(Oct)_2$ (1.00 x 10 $^{-2}$ M, 12.2 mg of $Sn(Oct)_2$ in 3.00 mL of THF) and GEO (0.03 M, 33.3 mg of GEO in 1.00 mL of THF) were prepared. The $Sn(Oct)_2$ (50.0 µL, 5.00 x 10 $^{-4}$ mmol, 1.0 equiv.) and GEO (400 µL, 1.33 x 10^{-2} mmol, 26.7 equiv.) stock solutions were mixed in a Schlenk before PLLA was added as a solid (144 mg, 2.00 mmol, 4000.0 equiv). The PLLA, $Sn(Oct)_2$ and GEO mixture was dissolved in a minimum amount of DCM (*approx.* 1 mL) before the solvent was removed *in-vacuuo*. A water-cooled cold-finger was connected to the Schlenk and the reaction mixture was heated to 160 °C with magnetic stirring under vacuum (0.1–1 mbar). After 5 h, a white crystalline powder was collected which was determined to be lactide (133 mg, 0.924 mmol, 92% yield, 95% *L*-LA as determined by ^{1}H NMR spectroscopy and GC-MS).

The sublimation apparatus was disassembled and a second batch of PLLA (144 mg, 2.00 mmol, 4000.00 equiv) was added as a solid to the ampoule containing the catalyst and GEO residue. The mixture was dissolved in a minium amount of DCM (*approx.* 1 mL) before the solvent was removed *in-vacuuo*. A water-cooled cold-finger was connected to the Schlenk and the reaction mixture was heated to 160 °C with magnetic stirring under vacuum (0.1–1 mbar). After 5 h, a white crystalline powder was collected which was determined to be lactide (135 mg, 0.940 mmol, 94% yield, 95% *L*-LA as determined by ^{1}H NMR spectroscopy and GC-MS). This process was repeated 2 more times (4 cylces in total). The mass recovered, yield and purity of the L-lactide in each cycle are given below:

Cycle 1: Mass of lactide recoverd = 133 mg; Yield = 92%; purity = 95% *L*-LA

Cycle 2: Mass of lactide recoverd = 135 mg; Yield = 94%; purity = 95% *L*-LA

Cycle 3: Mass of lactide recoverd = 140 mg; Yield = 97%; purity = 95% *L*-LA

Cycle 4: Mass of lactide recoverd = 137 mg; Yield = 95%; purity = 95% *L*-LA

2.4 Depolymerization of PLLA at Larger-scale

Under an N_2 atmosphere, PLLA (10.0 g, 138.8 mmol, 16063.0 equiv.) was added to an ampoule containing $Sn(Oct)_2$ (3.5 mg, 8.649 x 10^{-3} mmol, 1.0 equiv.) and GEO (925 mg, 0.9250 mmol, 106.9 equiv.). The PLLA, $Sn(Oct)_2$ and GEO mixture was dissolved in a minimum amount of DCM (*approx.* 10.0 mL) before the solvent was removed *in-vacuuo*. An ice cold-finger was connected to the Schlenk and the reaction mixture was heated to 160 °C under vacuum (0.1–1 mbar). After 24 h, a white crystalline powder was collected which was determined to be lactide (9.16 g, 63.59 mmol, 92% yield, 95% *L*-LA as determined by ^{1}H NMR spectroscopy and GC-MS).

2.5 Repolymerization of Recycled Lactide

In the glovebox, *L*-LA (144.1 mg, 1.0 mmol, 2000.0 equiv.) was added to a vial containing $Sn(Oct)_2$ (25 µL of a 2 x 10^{-2} M THF stock solution, 2.5 x 10^{-4} mmol, 1.0 equiv.), and 1,4-benzenedimethanol (25 µL of a 2 x 10^{-2} M THF stock solution, 2.5 x 10^{-4} mmol, 1.0 equiv.). The mixture was heated to 130 °C for 1 h before being quenched by addition of benzoic acid. The crude polymer was isolated by precipitation in TFA/MeOH (1:99) mixture (x 5) to yield PLLA as a white solid (100 mg, 69% yield)

3.0 Additional information

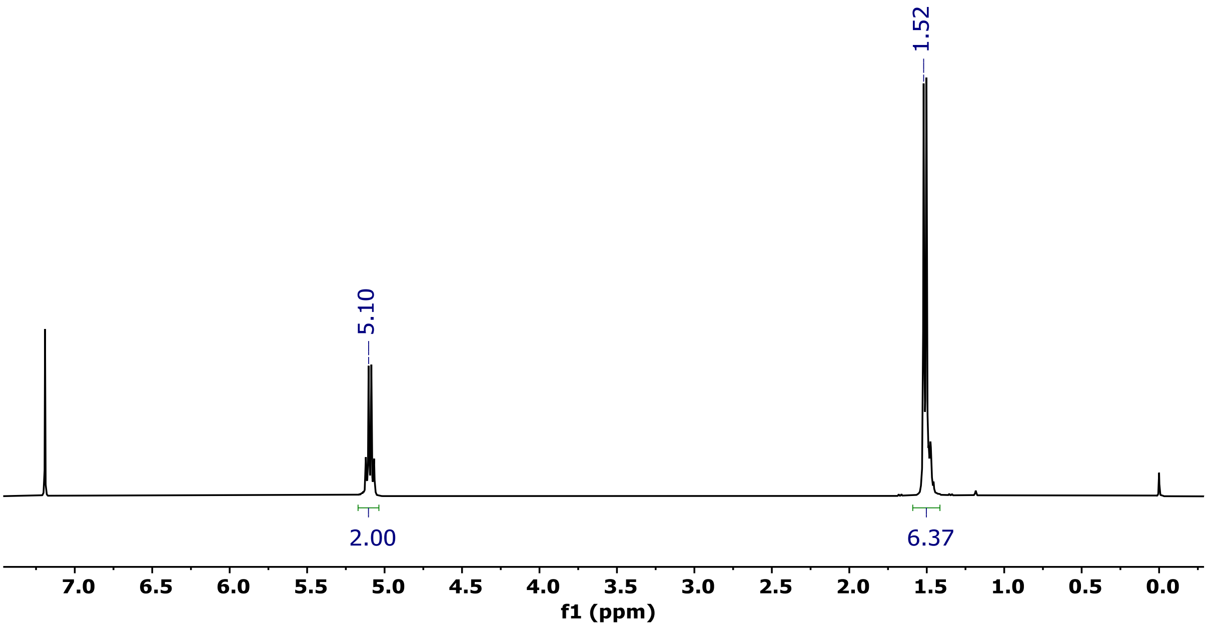

Figure S1. ^{1}H NMR spectrum (400 MHz, $CDCl_3$, 298 K) of commercial PLLA (5%-meso, see Fig. S2) used for study, δ 5.09 (q, J = 7.1 Hz, 2H, <u>**H**</u>CMe) and δ 1.51 (d, J = 7.2 Hz, 6H, HC<u>**Me**</u>).

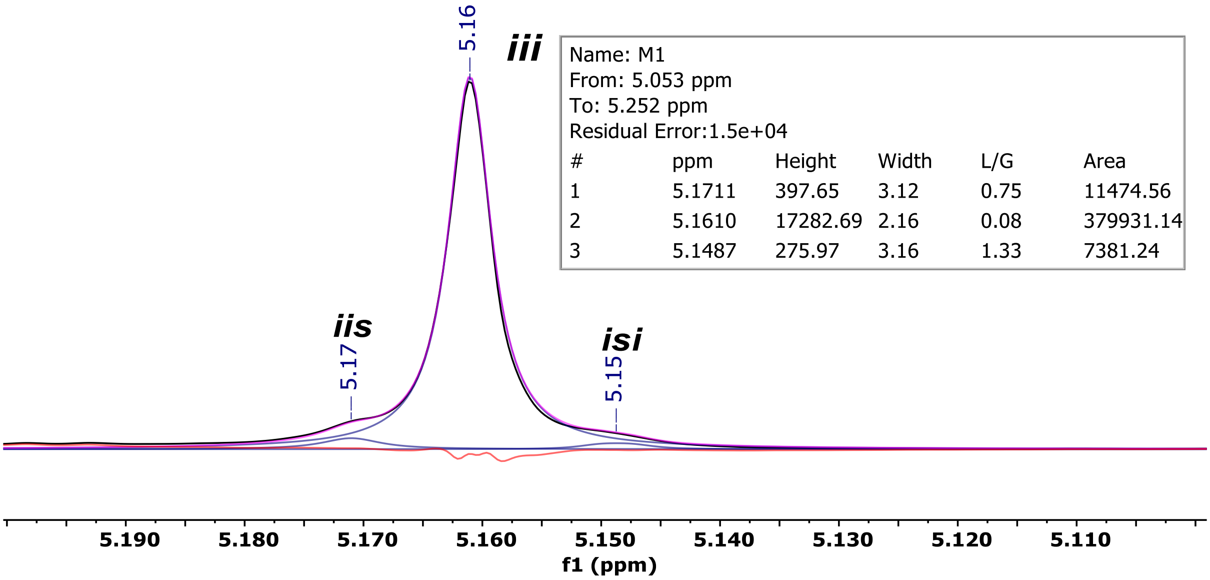

Figure S2. Homonuclear decoupled ^{1}H NMR spectrum ($CDCl_3$, 298K) of commercial PLLA used for study (normalized integrals of deconvoluted resonances: iis/ssi = 3%, iii = 95%, isi = 2%, consistent with approx. 5% D-lactic acid repeat units in PLLA sample)

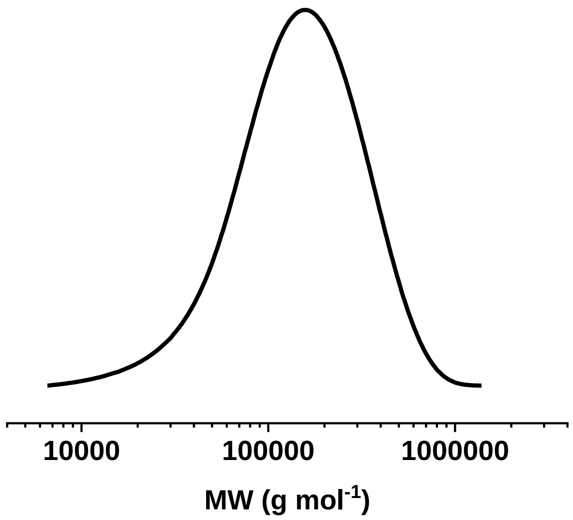

Figure S3. SEC ($CHCl_3$) of commercial PLLA used for study ($M_{n,SEC}$ of 60,000 g mol^{-1} correction factor of 0.58 applied [2])

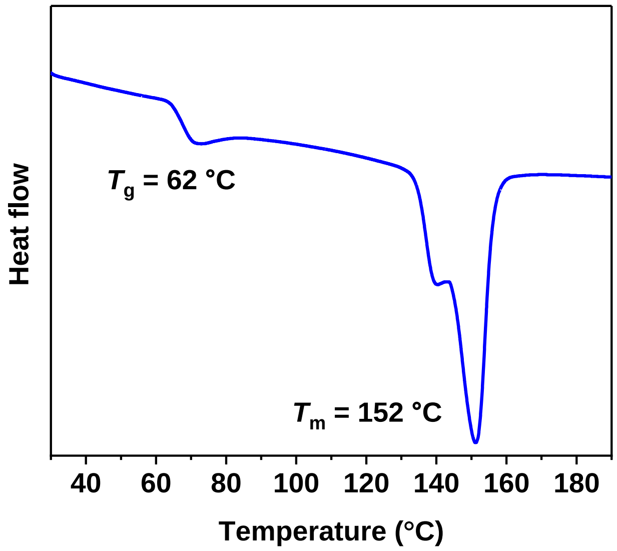

Figure S4. DSC thermogram of commercial PLLA used for study

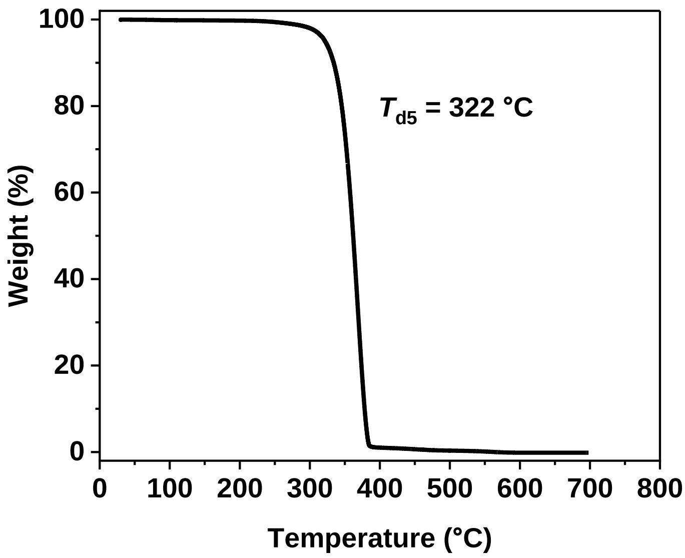

Figure S5. TGA thermogram of commercial PLLA used for study

End group analaysis by quanititative 31P NMR spectroscopy:

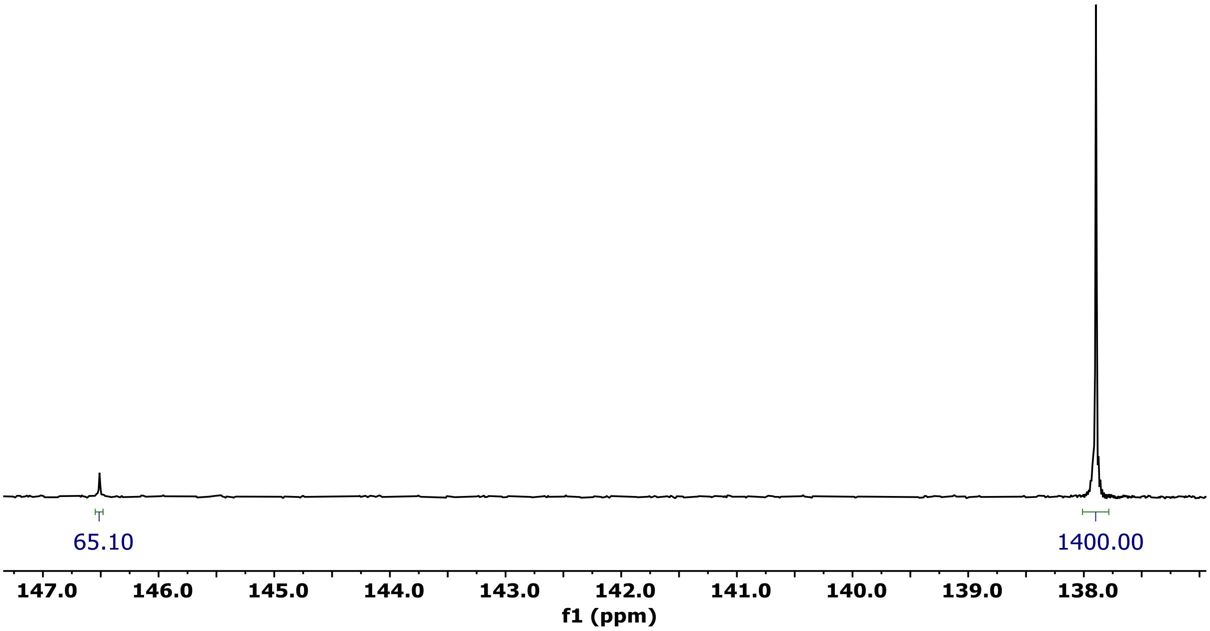

Figure S6. ^{31}P NMR (162 MHz, $CDCl_3$ spectroscopy) showing integral of internal BPA standard against PLA-OH end-groups. Integrals given in units of 1 x 10^7 mmols

Following an adapted literature method:[3]

Calculation for determination of end-groups with 41.3 mg of polymer present:

From SEC analysis, PLLA molar mass = 60,000 g mol^{-1}
Therefore, the *DP* of the PLLA is estimated to be 60,000/M_r of the lactic acid repeat unit
60,000/72.06 ≈ 833
When using 41.3 mg of PLLA, the moles of lactic acid repeat units = 41.3/72.06 = 0.573 mmols

For a monohydroxy terminated polymer, the moles of end group = moles of repeat units/ *DP*

$0.573/833 = 6.88 \times 10^{-4}$ mmols

For a dihydroxy α-ω telechelic polymer, the moles of end group = 2 x moles of repeat units/ *DP*
$2 \times 0.573/833 = 1.38 \times 10^{-3}$ mmols

By ^{31}P NMR spectroscopic end group titration (Fig S6), with 0.014 mmols of internal standard, for 41.3 mg of PLLA there are approximately 6.50×10^{-4} mmols of end group.

This suggests that the commercial goodfellows PLLA is monohydroxy terminated.

Table S2. Data for PLLA depolymerization using various metal salts

Entry	Catalyst	k_{obs} (h^{-1})[a]	TOF (h^{-1})[b]	Mass loss rate (g g^{-1} h^{-1})[d]	Residual mass (%)[e]
1	$SnCl_2$	14.6(±0.8)	144(±11)	54 (±4)	28%
2	$Zn(OAc)_2$	0.8(±0.1)	8(±1)	3.0(±0.2)	96%
3	$ZnCl_2$	1.4(±0.1)	13(±1)	7.0(±0.5)	93%
4	ZnI_2	1.4 (±0.1)	12(±1)	3.0(±0.2)	96%
5	$Ba(Oct)_2$	1.1(±0.1)	16(±1)	3.0(±0.2)	92%
6	$Fe(OAc)_2$	0.2(±0.1)	3(±0.2)	1.0(±0.1)	97%
7	$FeCl_2$	0.3(±0.1)	2(±0.2)	1.9(±0.1)	99%
8	$Mg(HMDS)_2$	0.5(±0.1)	5(±0.4)	1.1(±0.1)	97%
9	$Bi(Oct)_3$	10.8(±0.6)	107(±8)	12(±1)	46%
10	$Bi(OAc)_3$	15.2(±1.0)	138(±10)	25(±2)	31%
11	$Ti(Oct)_4$	0.2(±0.1)	3(±0.2)	0.30(±0.1)	99%
12	$Zr(Oct)_4$	0.4(±0.1)	3(±0.2)	0.4(±0.1)-	98%

TGA experiments run at 160 °C for 5 h or until >95% mass loss at $[cat]_0$:$[PLLA]_0$ loadings of 1:1000. Loadings calculated per M_r of PLLA repeat unit (M_r = 72.06 g mol^{-1}). [a] Linear fit of weight vs time profile. Average error taken from repeat runs. [c] TOF = moles of lactic acid repeat unit consumed (0-80% mass loss)/moles of catalyst/time(0-80% mass loss). if 80% mass loss not reached, TOF taken at 5 h. Average error taken from repeat runs. [d] Mass loss rate = TOF × M_r of PLLA repeat unit (72.06)/M_r of catalyst.[e] Residual mass at end of 5 h experiments.

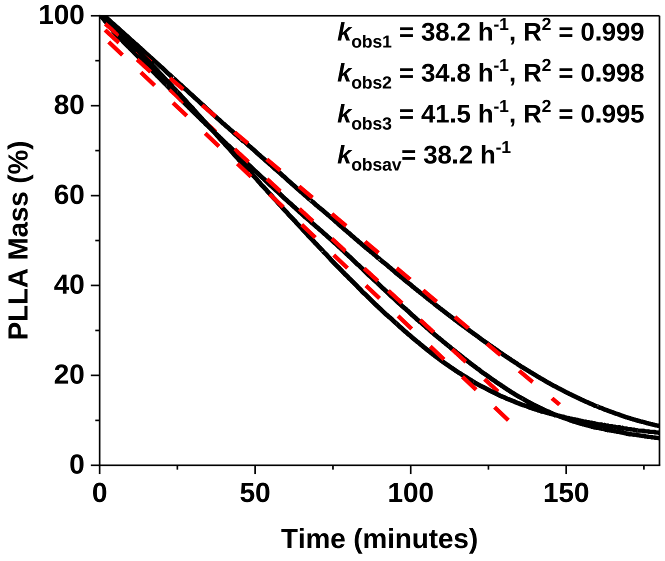

Figure S7. TGA thermograms showing repeat, independent experiments monitoring PLLA mass loss (black lines) and linear-fit of the mass loss trace (red-dashed lines) against time in $Sn(Oct)_2$:PLLA mixtures (1:1000, Table 1, entry 1). The TGAs were run with an isotherm at 160 °C until >95% mass loss

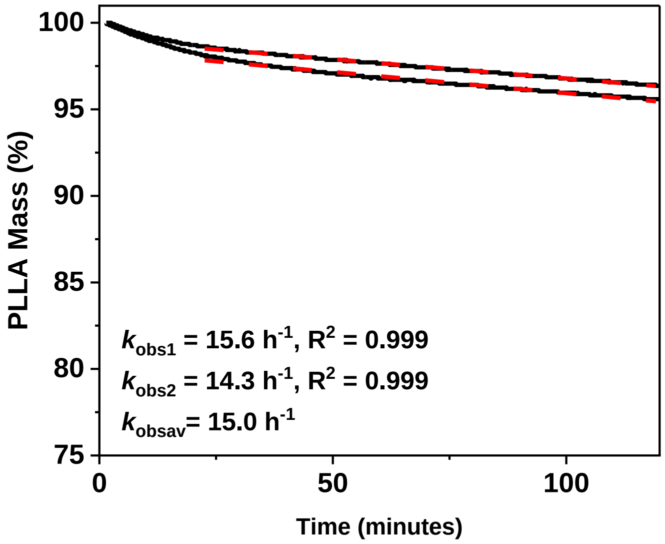

Figure S8. TGA thermograms showing repeat, independent experiments monitoring PLLA mass loss (black lines) and linear-fit of the mass loss trace (red-dashed lines) against time in $Zn(Oct)_2$:PLLA mixtures (1:1000, Table 1, entry 2). The TGAs were run with an isotherm at 160 °C for 5 h

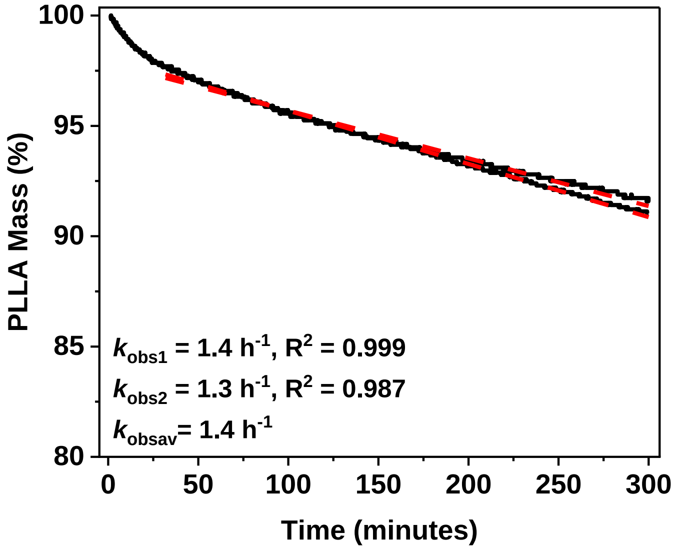

Figure S9. TGA thermograms showing repeat, independent experiments monitoring PLLA mass loss (black lines) and linear-fit of the mass loss trace (red-dashed lines) against time in $Ca(Oct)_2$:PLLA mixtures (1:1000, Table 1, entry 3). The TGAs were run with an isotherm at 160 °C for 5 h

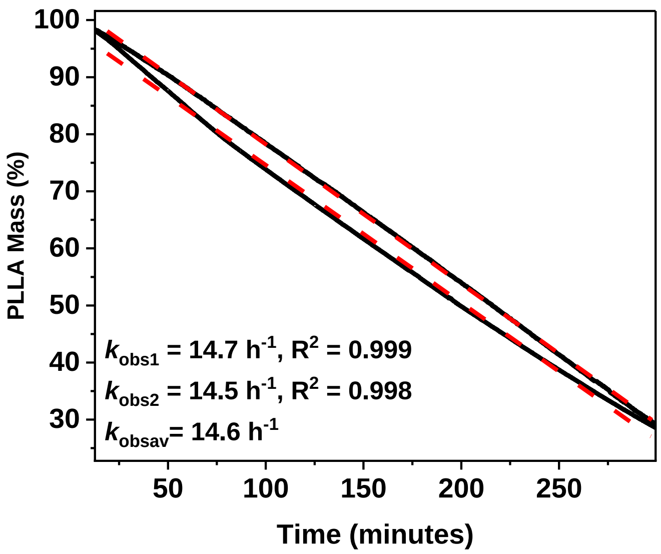

Figure S10. TGA thermograms showing repeat, independent experiments monitoring PLLA mass loss (black lines) and linear-fit of the mass loss trace (red-dashed lines) against time in $SnCl_2$:PLLA mixtures (1:1000, Table S2, entry 1). The TGAs were run with an isotherm at 160 °C for 5 h

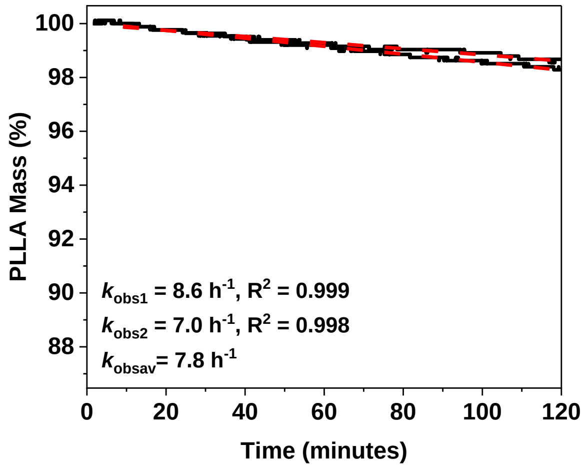

Figure S11. TGA thermograms showing repeat, independent experiments monitoring PLLA mass loss (black lines) and linear-fit of the mass loss trace (red-dashed lines) against time in $Zn(OAc)_2$:PLLA mixtures (1:1000, Table S2, entry 2). The TGAs were run with an isotherm at 160 °C for 5 h

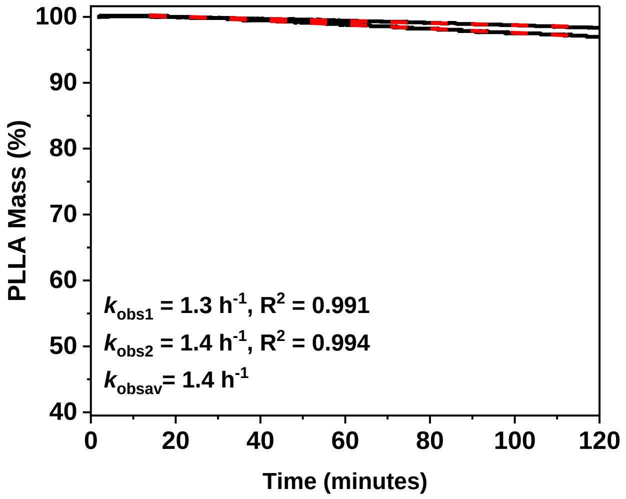

Figure S12. TGA thermograms showing repeat, independent experiments monitoring PLLA mass loss (black lines) and linear-fit of the mass loss trace (red-dashed lines) against time in $ZnCl_2$:PLLA mixtures (1:1000, Table S2, entry 3). The TGAs were run with an isotherm at 160 °C for 5 h

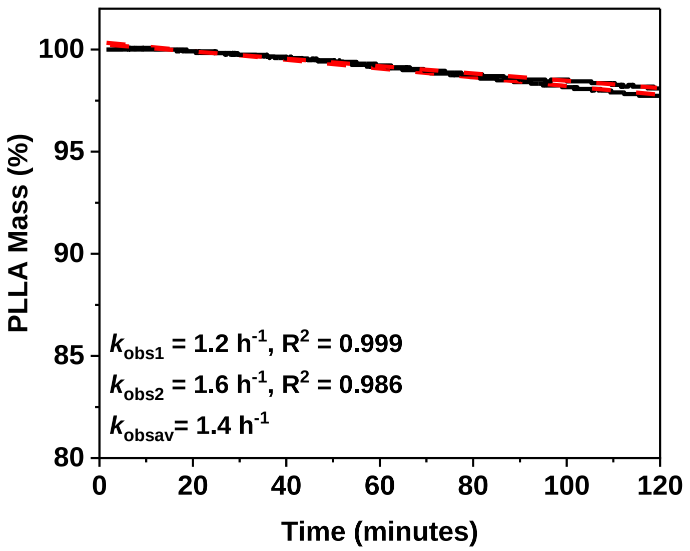

Figure S13. TGA thermograms showing repeat, independent experiments monitoring PLLA mass loss (black lines) and linear-fit of the mass loss trace (red-dashed lines) against time in ZnI_2:PLLA mixtures (1:1000, Table S2, entry 4). The TGAs were run with an isotherm at 160 °C for 5 h

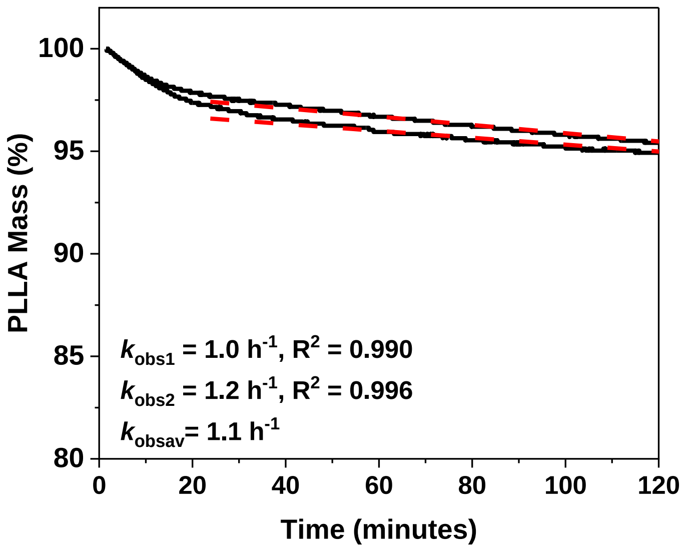

Figure S14. TGA thermograms showing repeat, independent experiments monitoring PLLA mass loss (black lines) and linear-fit of the mass loss trace (red-dashed lines) against time in $Ba(Oct)_2$:PLLA mixtures (1:1000, Table S2, entry 5). The TGAs were run with an isotherm at 160 °C for 5 h

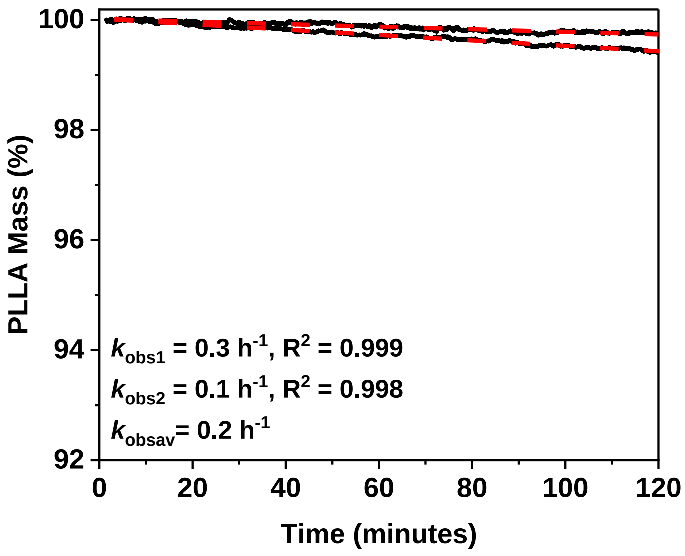

Figure S15. TGA thermograms showing repeat, independent experiments monitoring PLLA mass loss (black lines) and linear-fit of the mass loss trace (red-dashed lines) against time in $Fe(OAc)_2$:PLLA mixtures (1:1000, Table S2, entry 6). The TGAs were run with an isotherm at 160 °C for 5 h

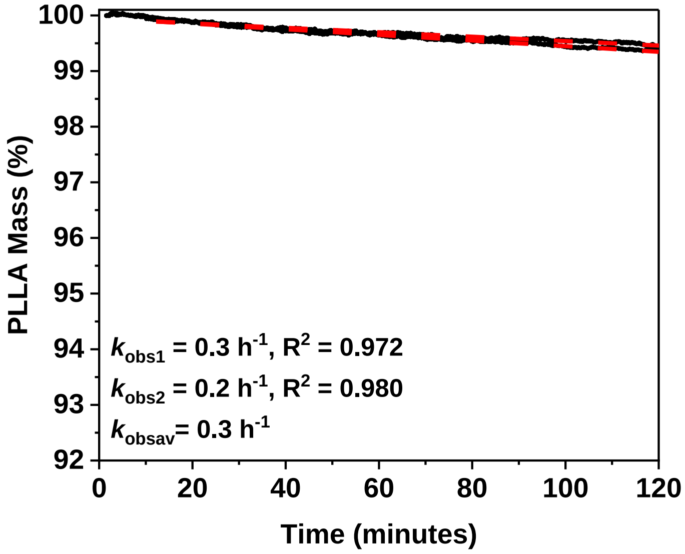

Figure S16. TGA thermograms showing repeat, independent experiments monitoring PLLA mass loss (black lines) and linear-fit of the mass loss trace (red-dashed lines) against time in $FeCl_2$:PLLA mixtures (1:1000, Table S2, entry 7). The TGAs were run with an isotherm at 160 °C for 5 h

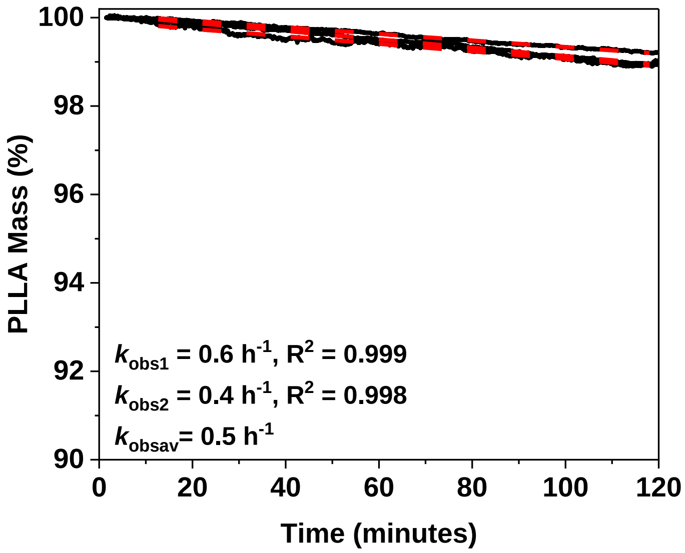

Figure S17. TGA thermograms showing repeat, independent experiments monitoring PLLA mass loss (black lines) and linear-fit of the mass loss trace (red-dashed lines) against time in $Mg(HMDS)_2$:PLLA mixtures (1:1000, Table S2, entry 8). The TGAs were run with an isotherm at 160 °C for 5 h

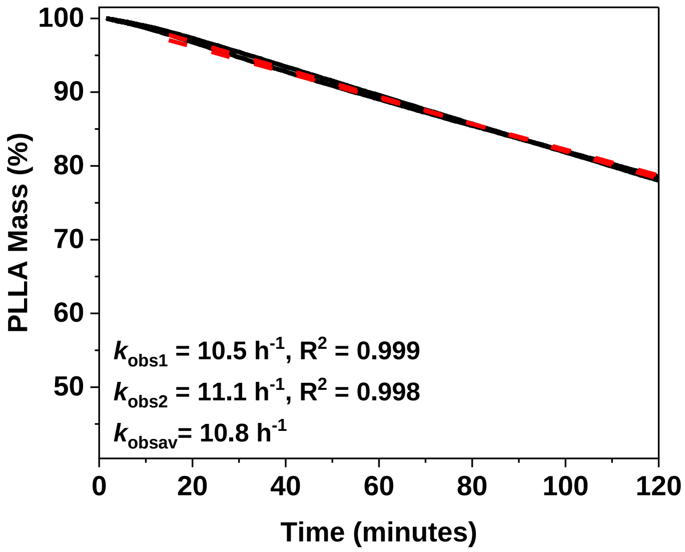

Figure S18. TGA thermograms showing repeat, independent experiments monitoring PLLA mass loss (black lines) and linear-fit of the mass loss trace (red-dashed lines) against time in $Bi(Oct)_3$:PLLA mixtures (1:1000, Table S2, entry 9). The TGAs were run with an isotherm at 160 °C for 5 h

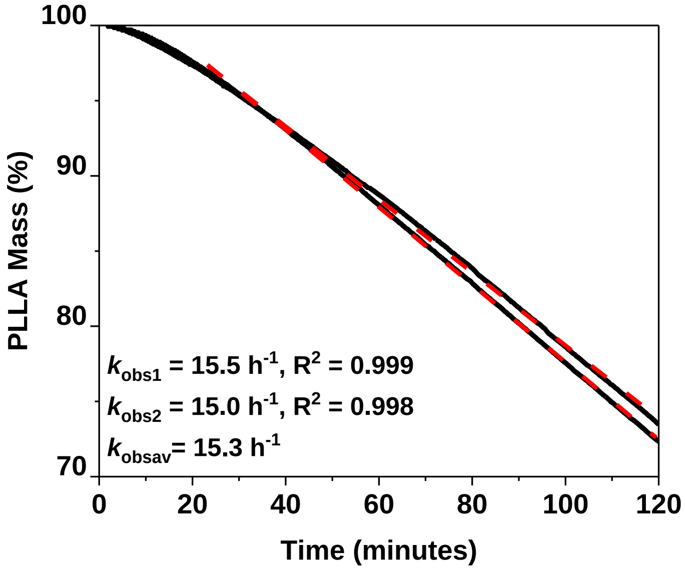

Figure S19. TGA thermograms showing repeat, independent experiments monitoring PLLA mass loss (black lines) and linear-fit of the mass loss trace (red-dashed lines) against time in $Bi(OAc)_3$:PLLA mixtures (1:1000, Table S2, entry 10). The TGAs were run with an isotherm at 160 °C for 5 h

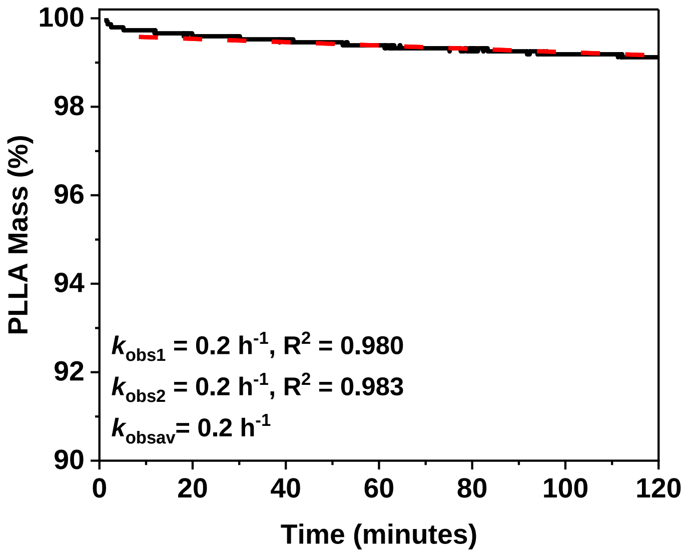

Figure S20. TGA thermograms showing repeat, independent experiments monitoring PLLA mass loss (black lines) and linear-fit of the mass loss trace (red-dashed lines) against time in $Ti(Oct)_4$:PLLA mixtures (1:1000, Table S2, entry 11). The TGAs were run with an isotherm at 160 °C for 5 h

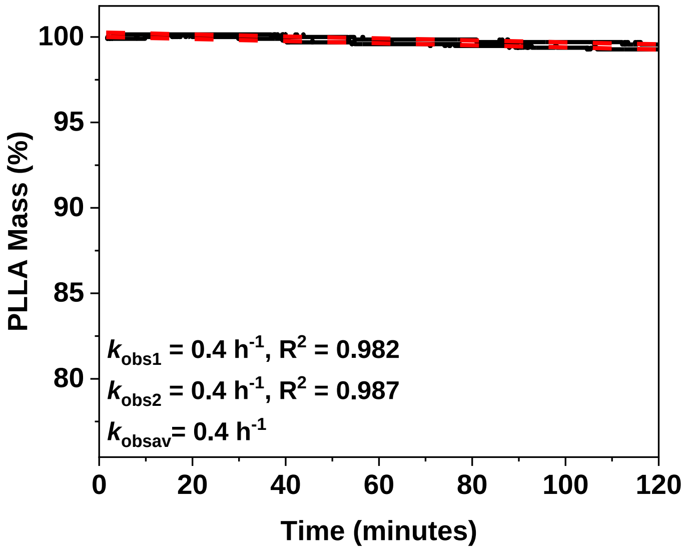

Figure S21. TGA thermograms showing repeat, independent experiments monitoring PLLA mass loss (black lines) and linear-fit of the mass loss trace (red-dashed lines) against time in $Zr(Oct)_4$:PLLA mixtures (1:1000, Table S2, entry 12). The TGAs were run with an isotherm at 160 °C for 5 h

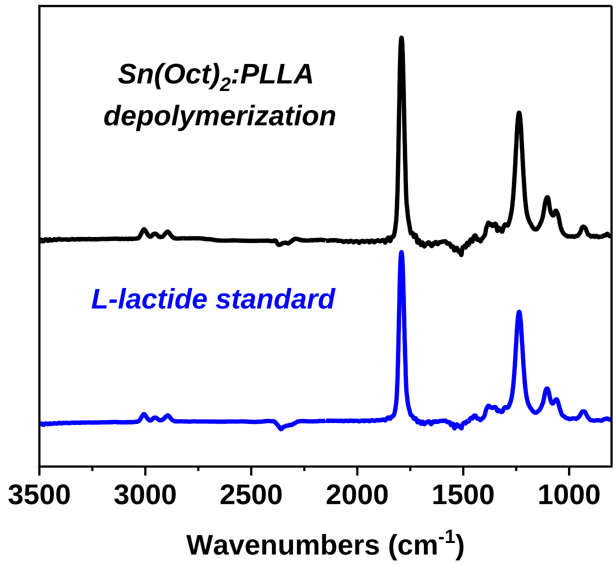

Figure S22. TGA-FTIR gas-phase analysis of PLLA depolymerization mixture catalysed by $Sn(Oct)_2$ ($[Sn(Oct)_2]_0$:$[PLLA]_0$ = 1:1000, 160 °C, black) and an L-lactide standard (blue).

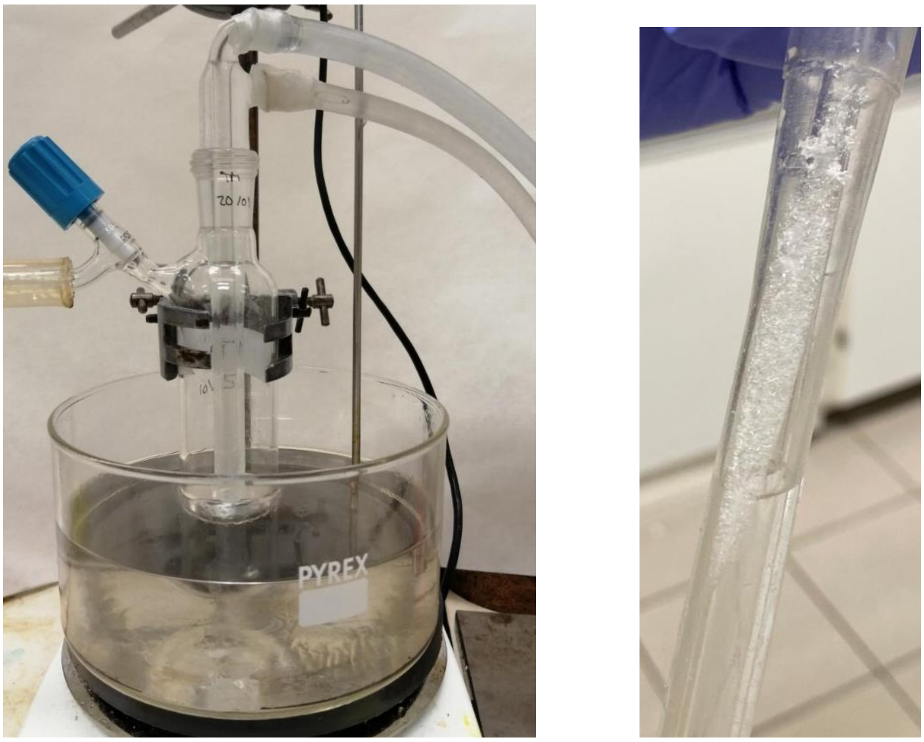

Figure S23. (left) Setup used for depolymerization-sublimation of PLLA to LA. (right) Lactide collected on a cold finger

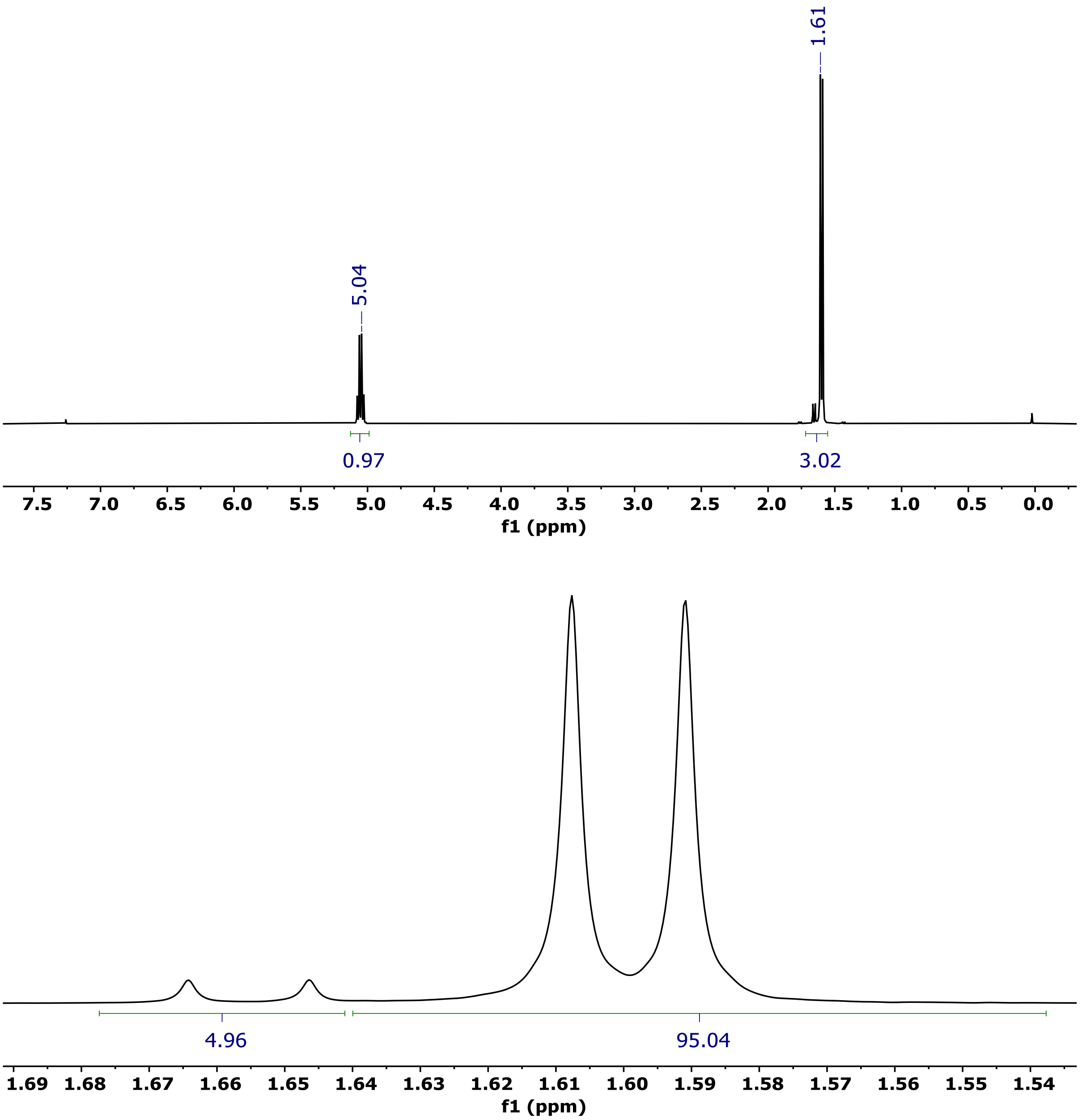

Figure S24. ^{1}H NMR (400 MHz, $CDCl_3$, 298K) spectrum of LA isolated from depolymerization of PLLA ($[Sn(Oct)_2]_0$:$[PLLA]_0$ 1:1000, 160 °C, Table 1, entry 1) δ 5.05 (q, *J* = 6.7 Hz, 2H, L,L + D,L **H**CMe), 1.66 (d, *J* = 7.1 Hz, *trace (5% meso)*, D,L HC**Me**)δ 1.60 (d, *J* = 6.7 Hz, 6H, L,L HC**Me**).

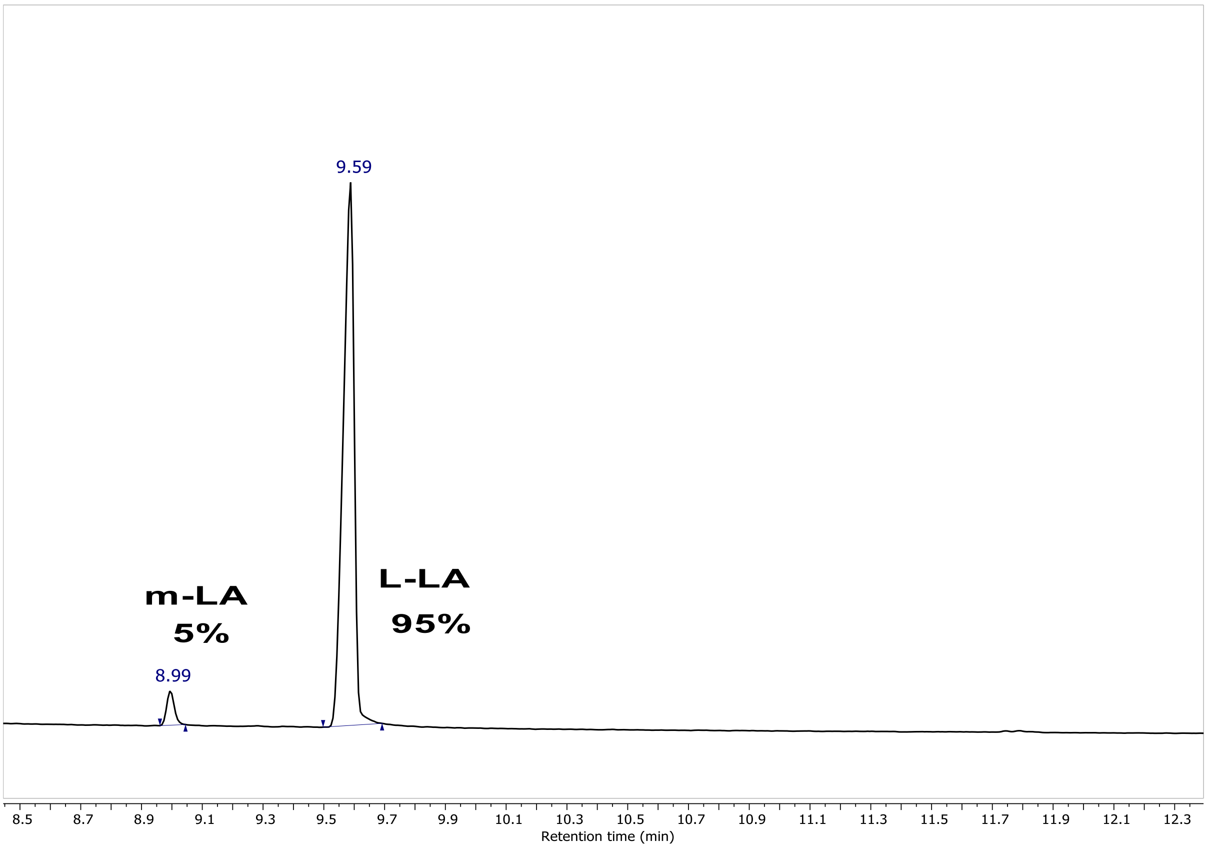

Figure S25. GC of LA isolated from depolymerization of PLLA ($[Sn(Oct)_2]_0$:$[PLLA]_0$ 1:1000, 160 °C, Table 1, entry 1, *m*-LA = 5%, *L*-LA = 95%)

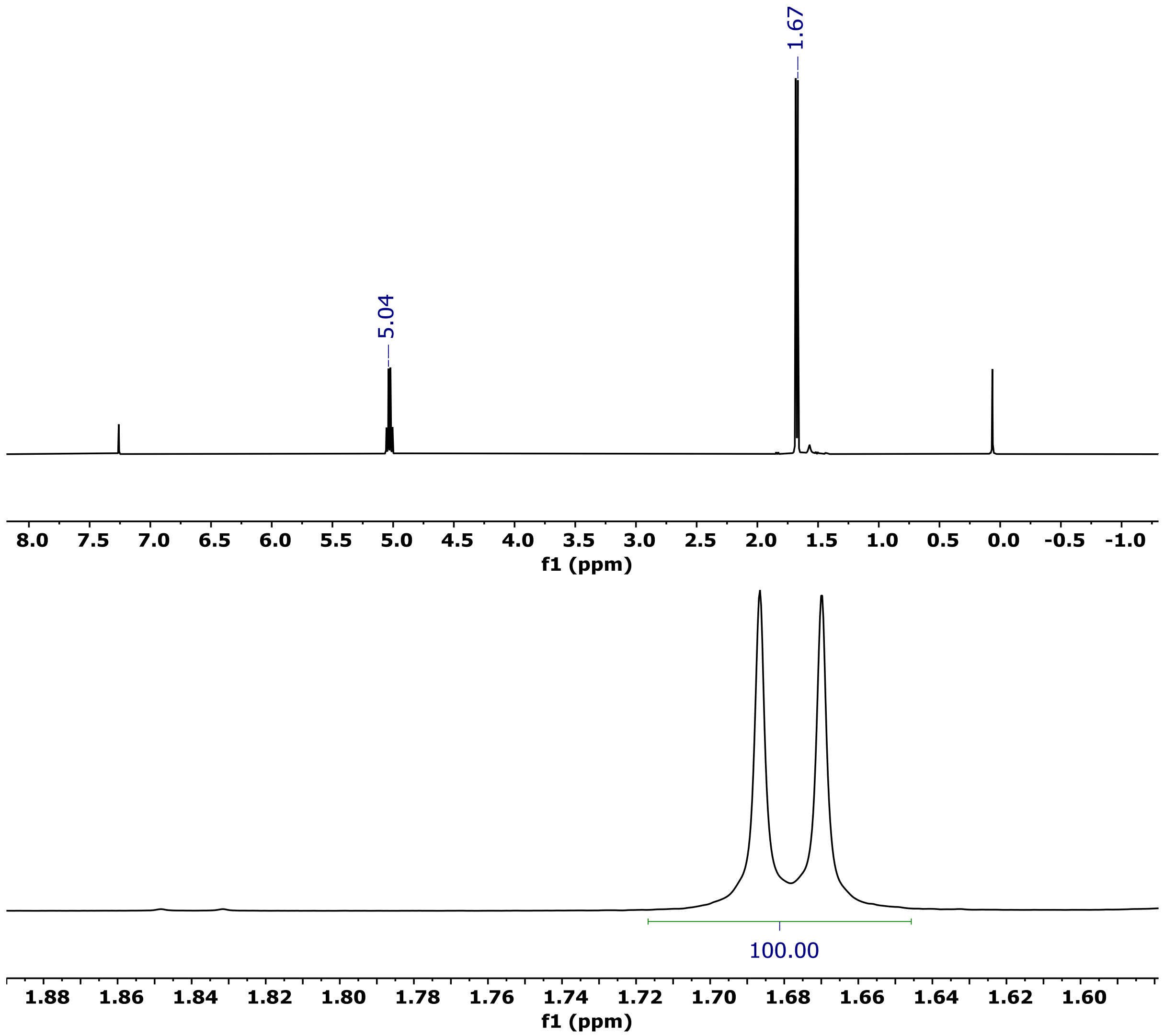

Figure S26. ^{1}H NMR (400 MHz, $CDCl_3$, 298K) spectrum of L-LA isolated from depolymerization of 100%-PLLA ($[Sn(Oct)_2]_0$:$[PLLA]_0$ 1:1000, 160 °C) δ 5.04 (q, *J* = 6.7 Hz, 2H, L,L-LA, <u>H</u>CMe), δ 1.67 (d, *J* = 6.7 Hz, 6H, L,L-LA HC<u>Me</u>).

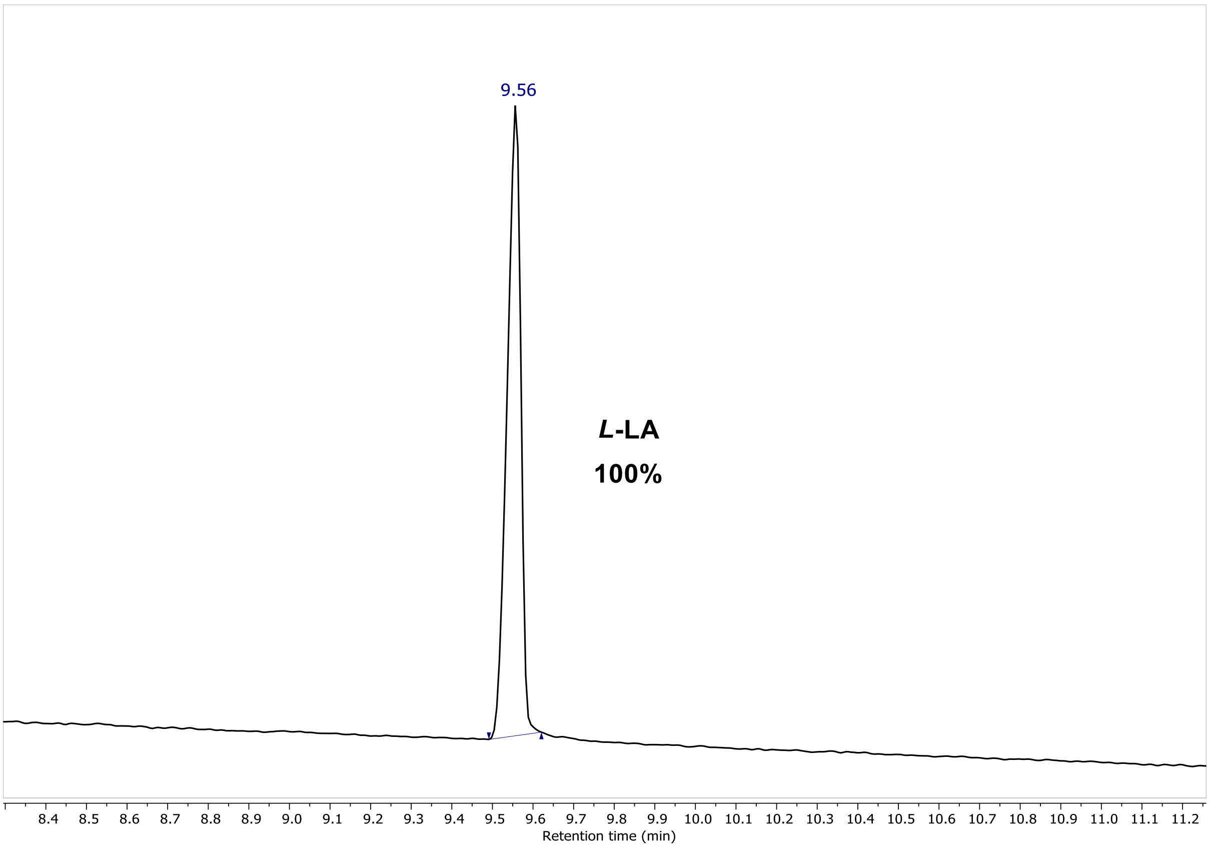

Figure S27. GC of L-LA isolated from depolymerization of 100%-PLLA ($[Sn(Oct)_2]_0$:$[PLLA]_0$ 1:1000, 160 °C, *L*-LA = 100%)

Table S3. Depolymerization of PLLA at various reaction temperatures

Entry	Temp.(°C)	k_{obs} (h^{-1})[a]	TOF (h^{-1})[b]	Mass loss rate ($g\ g^{-1}\ h^{-1}$)[d]	Residual mass[e]
1	160	38.2 (±2.2)	380 (±28)	67 (±5)	<5%
2	165	51.7 (±8.1)	536 (±90)	95 (±15)	<5%
3	170	79.8 (±2.7)	790 (±40)	139 (±9)	<5%
4	175	103.8 (±6.1)	1100 (±230)	215 (±11)	<5%
5	180	152.9 (±23.6)	1440 (±240)	269 (±43)	<5%

TGA experiments run for 5 h at given temperature or until >95% mass loss at $[cat]_0$:$[PLLA]_0$ loadings of 1:1000. Loadings calculated per M_r of PLLA repeat unit (M_r = 72.06 g mol^{-1}). [a] Linear fit of weight vs time profile. Average error taken from repeat runs. [c] TOF = moles of lactic acid repeat unit consumed (0-80% mass loss)/moles of catalyst/time(0-80% mass loss); if 80% mass loss not reached, TOF taken at 5 h. Average error taken from repeat runs. [d] Mass loss rate = TOF × M_r of PLLA repeat unit (72.06)/M_r of catalyst. [e] Residual mass at end of 5 h experiments.

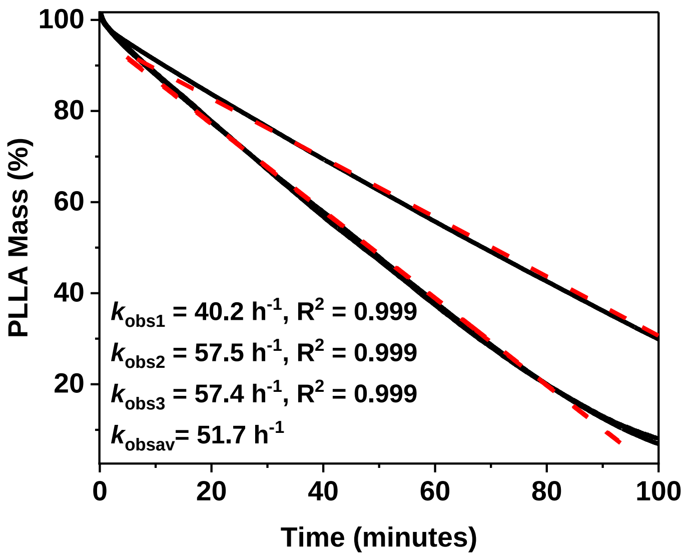

Figure S28. TGA thermograms showing repeat, independent experiments monitoring PLLA mass loss (black lines) and linear-fit of the mass loss trace (red-dashed lines) against time in $Sn(Oct)_2$:PLLA mixtures (1:1000, Table S3, entry 2). The TGAs were run with an isotherm at 165 °C until >95% mass loss

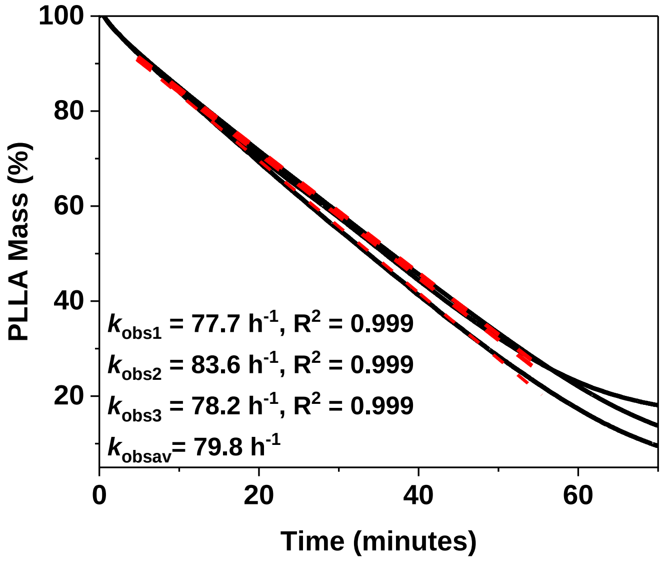

Figure S29. TGA thermograms showing repeat, independent experiments monitoring PLLA mass loss (black lines) and linear-fit of the mass loss trace (red-dashed lines) against time in $Sn(Oct)_2$:PLLA mixtures (1:1000, Table 1, entry 4 and Table S3, entry 3). The TGAs were run with an isotherm at 170 °C until >95% mass loss

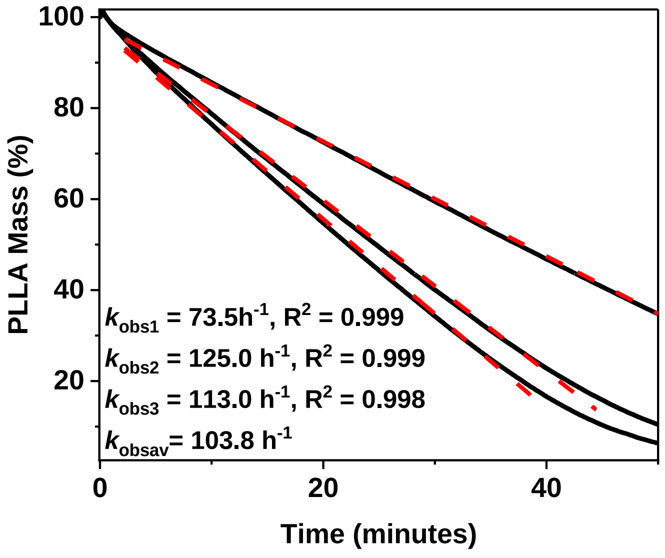

Figure S30. TGA thermograms showing repeat, independent experiments monitoring PLLA mass loss (black lines) and linear-fit of the mass loss trace (red-dashed lines) against time in $Sn(Oct)_2$:PLLA mixtures (1:1000, Table S3, entry 4). The TGAs were run with an isotherm at 175 °C until >95% mass loss

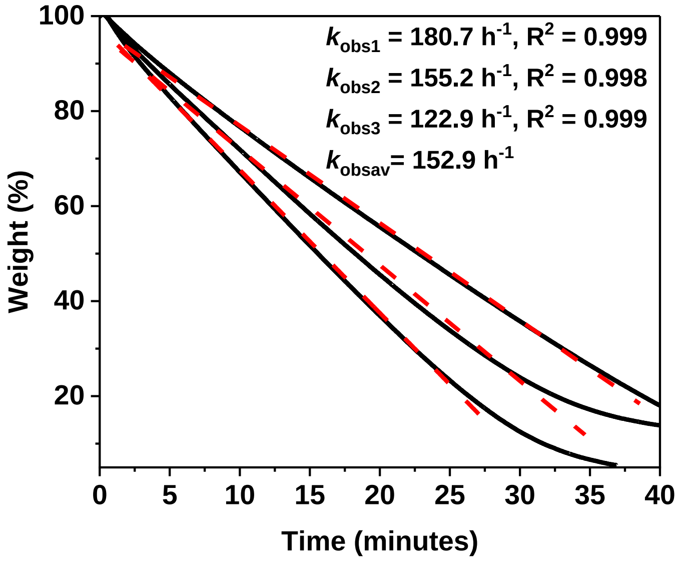

Figure S31. TGA thermograms showing repeat, independent experiments monitoring PLLA mass loss (black lines) and linear-fit of the mass loss trace (red-dashed lines) against time in $Sn(Oct)_2$:PLLA mixtures (1:1000, Table 1, entry 5 and Table S3, entry 5). The TGAs were run with an isotherm at 180 °C until >95% mass loss

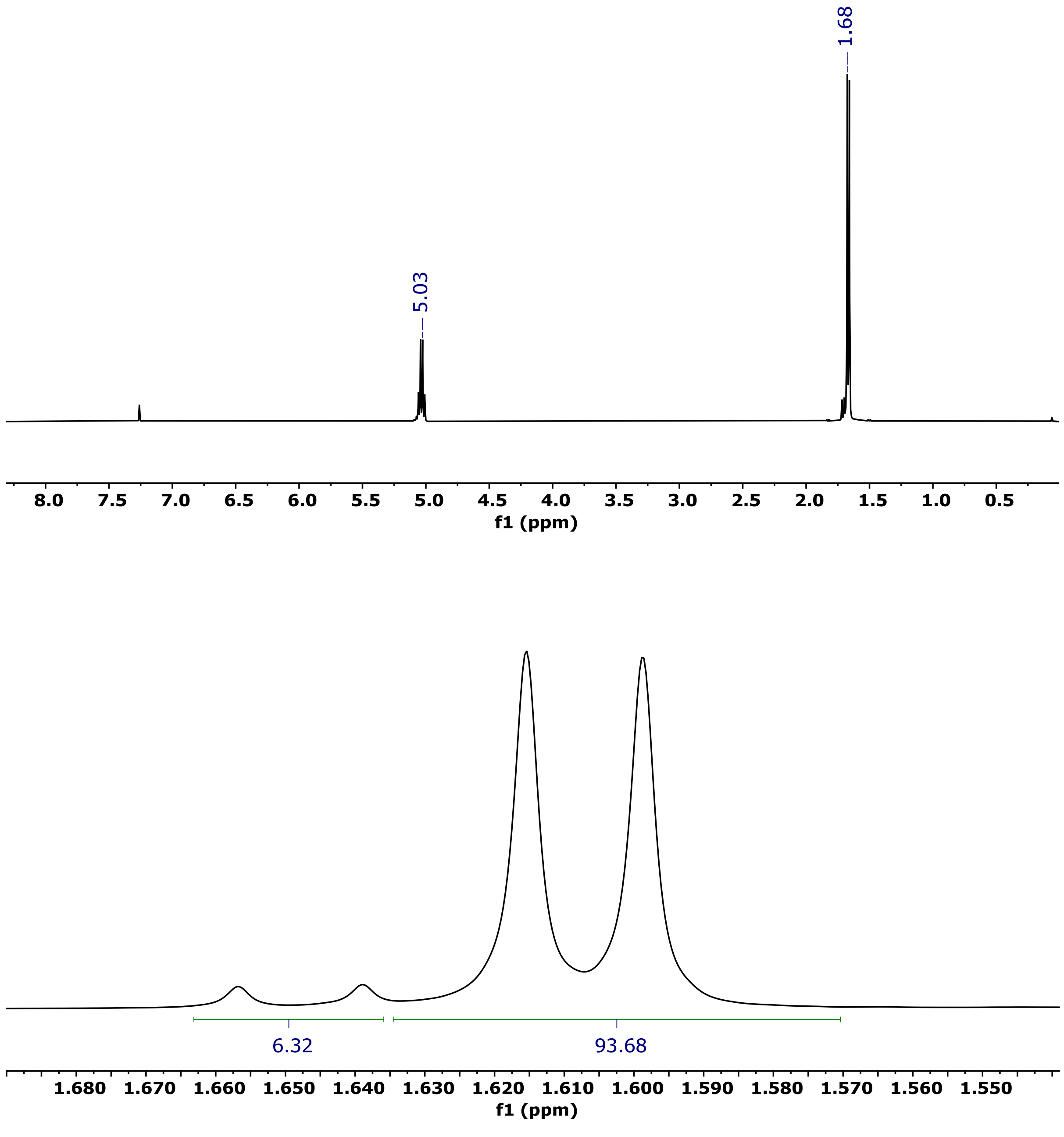

Figure S32. ^{1}H NMR (400 MHz, $CDCl_3$, 298K) spectrum of LA isolated from depolymerization of PLLA ($[Sn(Oct)_2]_0$:$[PLLA]_0$ 1:1000, 180 °C, Table 1, entry 5) δ 5.05 (q, *J* = 6.7 Hz, 2H, L,L + D,L <u>**H**</u>CMe), 1.66 (d, *J* = 7.1 Hz, *trace (6% meso)*, D,L HC<u>**Me**</u>)δ 1.60 (d, *J* = 6.7 Hz, 6H, L,L HC<u>**Me**</u>).

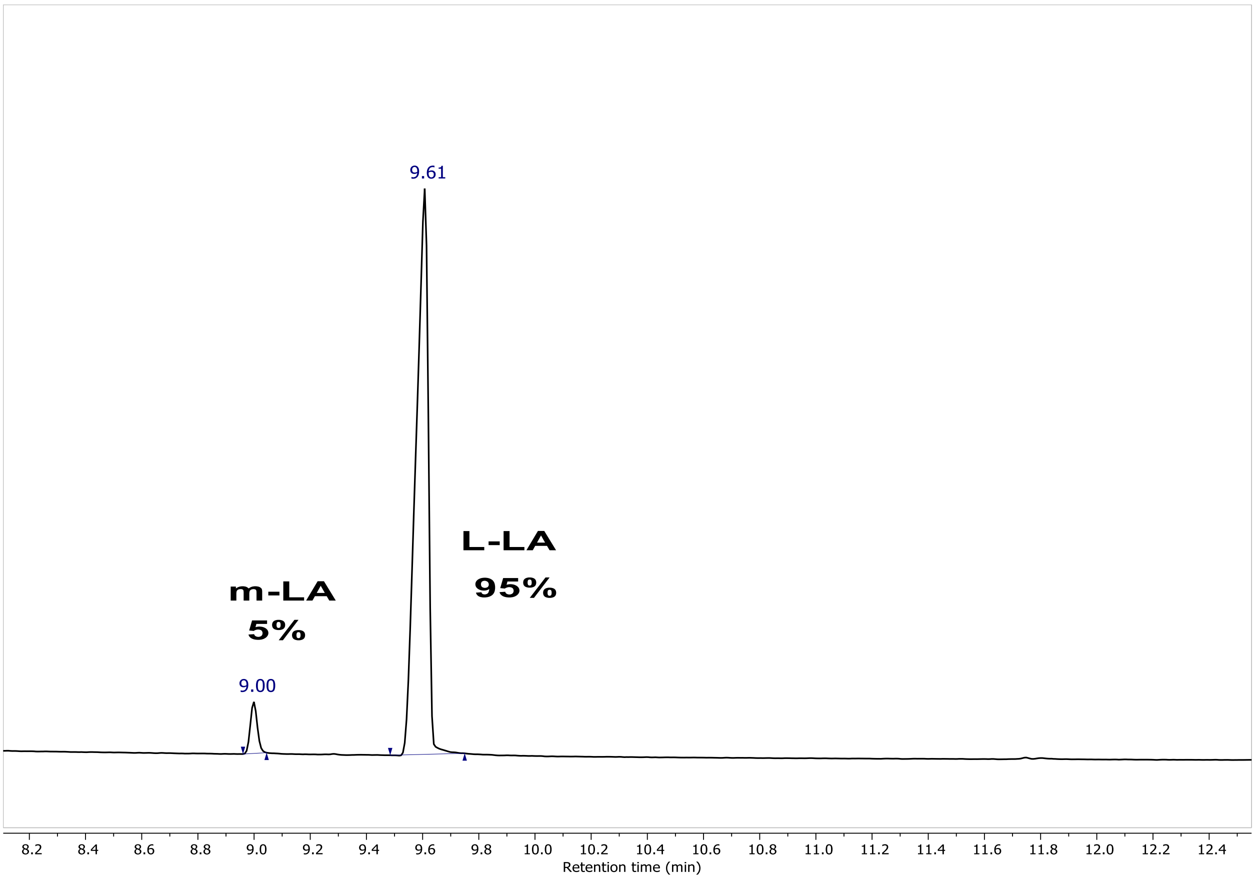

Figure S33. GC of LA isolated from depolymerization of PLLA ($[Sn(Oct)_2]_0$:$[PLLA]_0$ 1:1000, 180 °C, Table 1, entry 5, *m*-LA = 5%, *L*-LA = 95%)

Table S4. Depolymerization of PLLA at various $Sn(Oct)_2$ loadings

Entry	Loading	k_{obs} (h^{-1})[a]	TOF (h^{-1})[b]	Mass loss rate ($g\ g^{-1}\ h^{-1}$)	Residual mass[c]
1	1: 1000	38.2(±2.7)	386(±28)	65(±5)	<5
2	0.5: 1000	18.2 (±2.6)	370 (±50)	72 (±1)	<5
3	0.33: 1000	12.3 (±1.9)	380 (±50)	73 (±6)	25%
4	0.25: 1000	9.8 (±1.0)	400 (±40)	72 (±7)	47%
5	0.2: 1000	7.8 (±1.7)	374 (±50)	66 (±10)	65%
6	0.167: 1000	5.0(±0.3)	370 (±50)	72 (±1)	74%
7	0.125: 1000	0.8(±0.1)	75(±5)	15(±1)	90%
8	0.1:1000	1.1(±0.1)	123(±9)	21(±1)	95%

Reactions prepared by solvent casting polymer:catalyst solutions in TGA crucibles and PLLAcing under high vacuum for 1 h, with $[PLLA]_0$ = 1.0 M solution in DCM. Reactions performed with N_2 flow at 25 mL/min on TGA using the following method: 1) jump from 30 to 160 °C 2) isotherm at 160 °C for 5 h or until 95% mass loss. [a] k_{obs} = linear fit of mass loss *vs* time. [b] TOF = moles of lactic acid repeat consumed between 0-80% mass loss/ moles of catalyst/time. [c] Residual mass at end of 5 h experiments

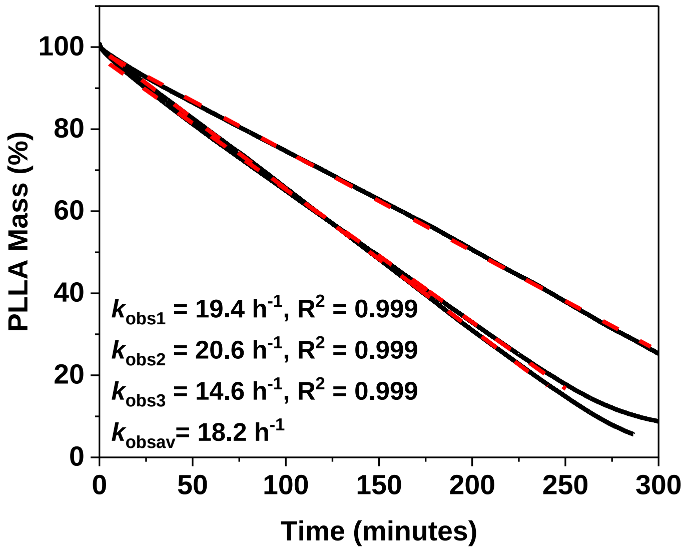

Figure S34. TGA thermograms showing repeat, independent experiments monitoring PLLA mass loss (black lines) and linear-fit of the mass loss trace (red-dashed lines) against time in $Sn(Oct)_2$:PLLA mixtures (0.5: 1000, Table 1, entry 6 and Table S4, entry 2). The TGAs were run with an isotherm at 160 °C until >95% mass loss

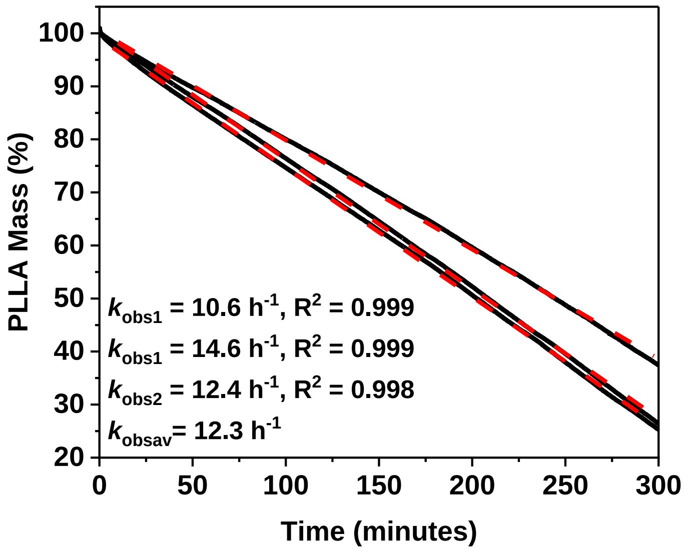

Figure S35. TGA thermograms showing repeat, independent experiments monitoring PLLA mass loss (black lines) and linear-fit of the mass loss trace (red-dashed lines) against time in $Sn(Oct)_2$:PLLA mixtures (0.33: 1000, Table 1, entry 7 and Table S4, entry 3). The TGAs were run with an isotherm at 160 °C for 5 h

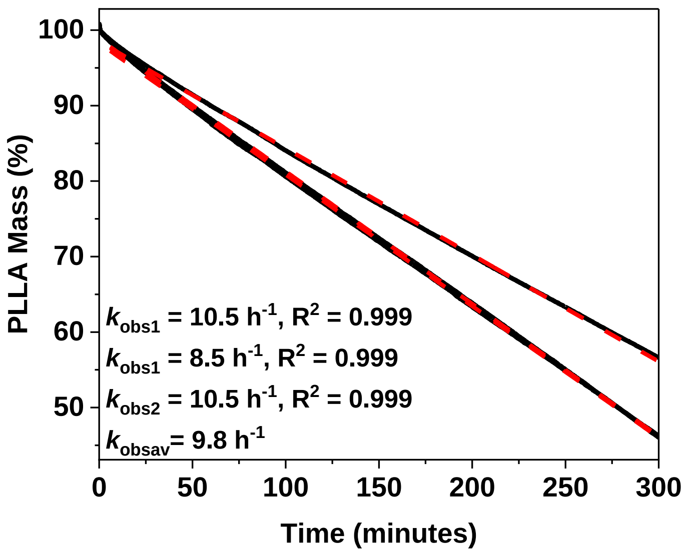

Figure S36. TGA thermograms showing repeat, independent experiments monitoring PLLA mass loss (black lines) and linear-fit of the mass loss trace (red-dashed lines) against time in $Sn(Oct)_2$:PLLA mixtures (0.25: 1000, Table 1, entry 8 and Table S4, entry 4). The TGAs were run with an isotherm at 160 °C for 5 h

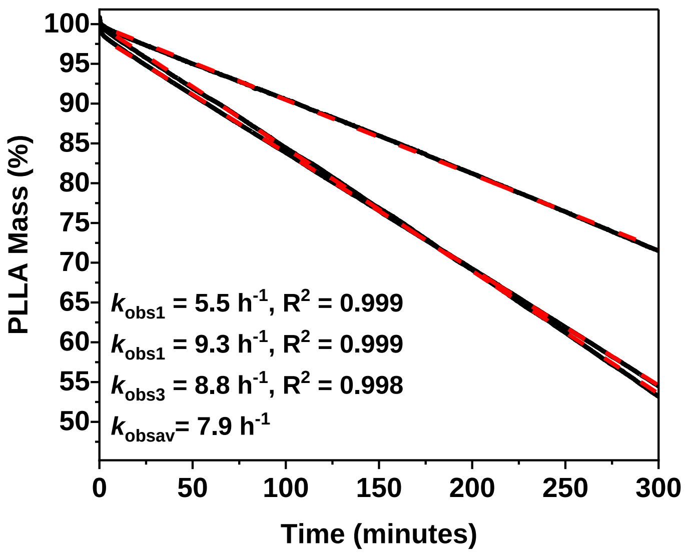

Figure S37. TGA thermograms showing repeat, independent experiments monitoring PLLA mass loss (black lines) and linear-fit of the mass loss trace (red-dashed lines) against time in $Sn(Oct)_2$:PLLA mixtures (0.2: 1000, Table 1, entry 9 and Table S4, entry 5). The TGAs were run with an isotherm at 160 °C for 5 h

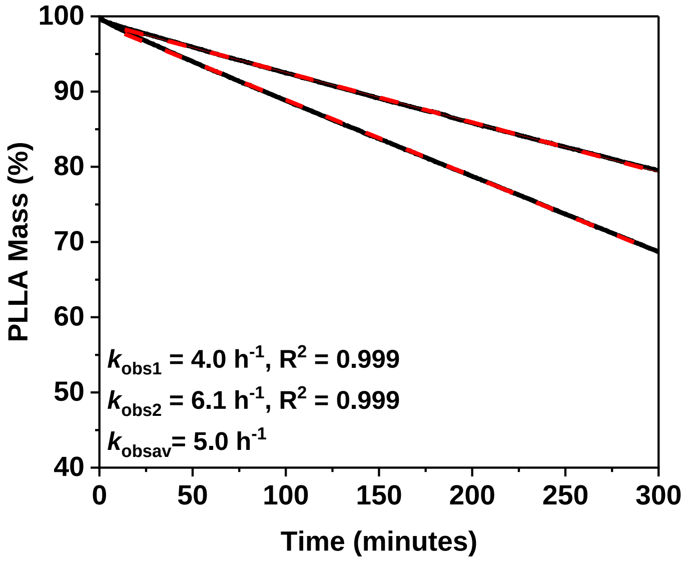

Figure S38. TGA thermograms showing repeat, independent experiments monitoring PLLA mass loss (black lines) and linear-fit of the mass loss trace (red-dashed lines) against time in $Sn(Oct)_2$:PLLA mixtures (0.167: 1000, Table S4, entry 6). The TGAs were run with an isotherm at 160 °C for 5 h

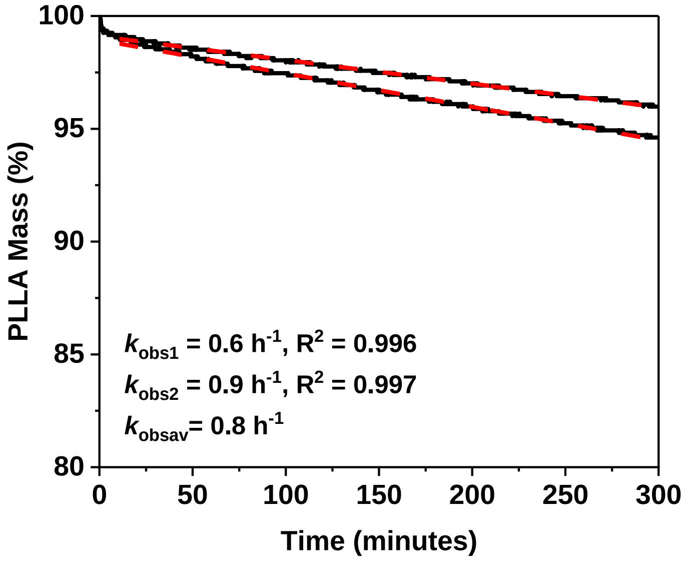

Figure S39. TGA thermograms showing repeat, independent experiments monitoring PLLA mass loss (black lines) and linear-fit of the mass loss trace (red-dashed lines) against time in $Sn(Oct)_2$:PLLA mixtures (0.125: 1000 Table S4, entry 7). The TGAs were run with an isotherm at 160 °C for 5 h

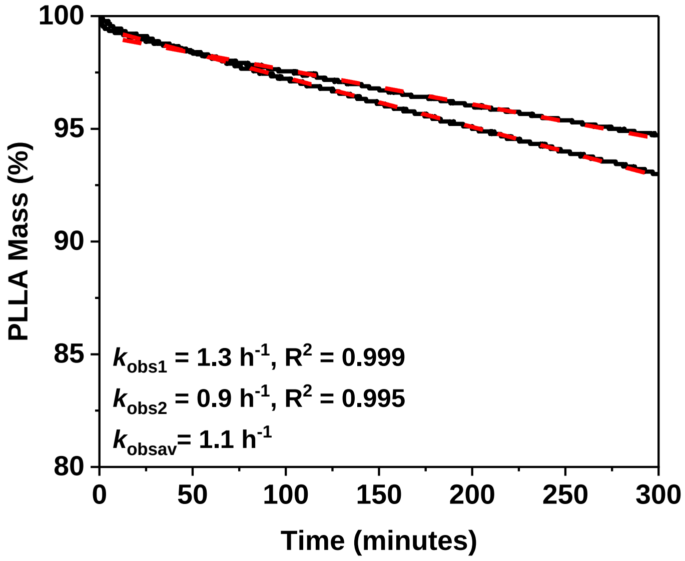

Figure S40. TGA thermograms showing repeat, independent experiments monitoring PLLA mass loss (black lines) and linear-fit of the mass loss trace (red-dashed lines) against time in $Sn(Oct)_2$:PLLA mixtures (0.1:1000, Table S4, entry 8). The TGAs were run with an isotherm at 160 °C for 5 h

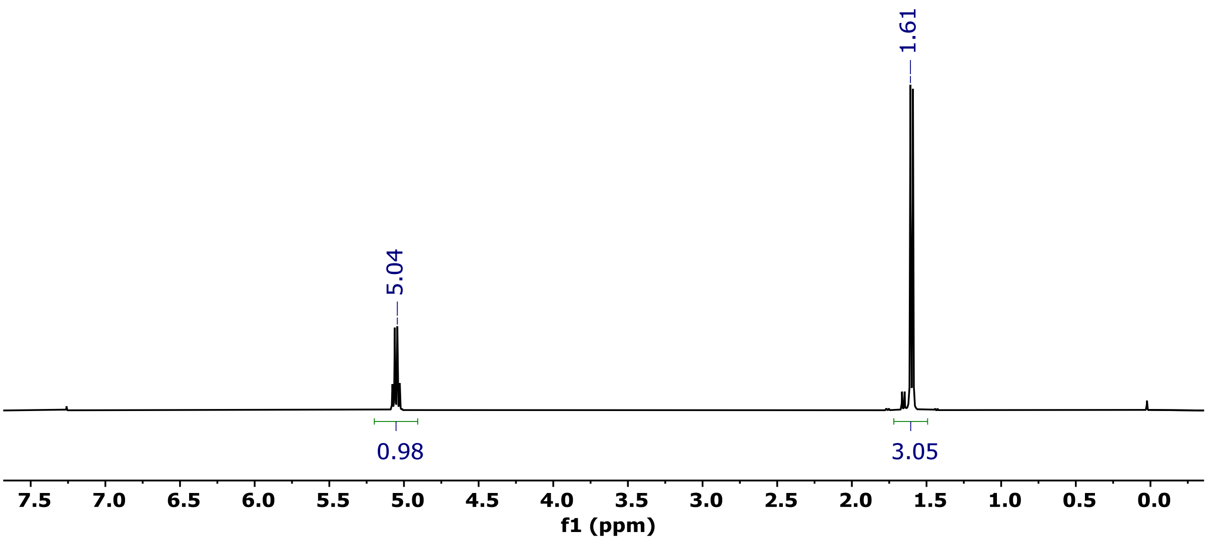

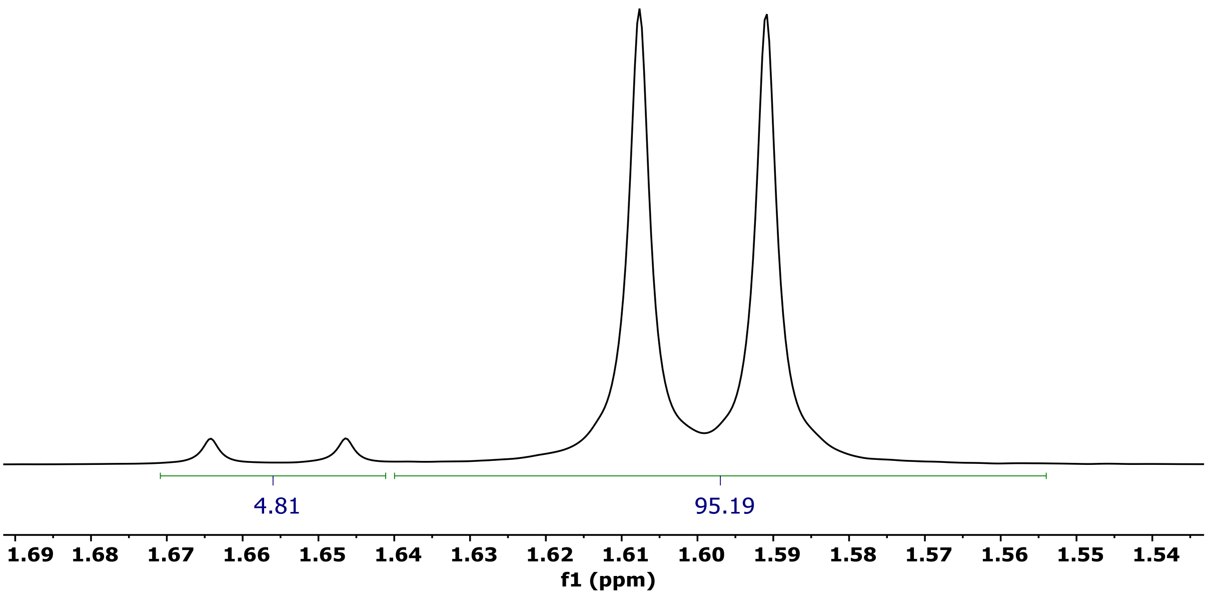

Figure S41. ^{1}H NMR (400 MHz, $CDCl_3$, 298K) spectrum of LA isolated from depolymerization of PLLA ($[Sn(Oct)_2]_0$:$[PLLA]_0$ 0.25: 1000, 160 °C, Table 1, entry 8) δ 5.05 (q, *J* = 6.7 Hz, 2H, L,L + D,L **H**CMe), 1.66 (d, *J* = 7.1 Hz, *trace (5% meso)*, D,L HC**Me**)δ 1.60 (d, *J* = 6.7 Hz, 6H, L,L HC**Me**).

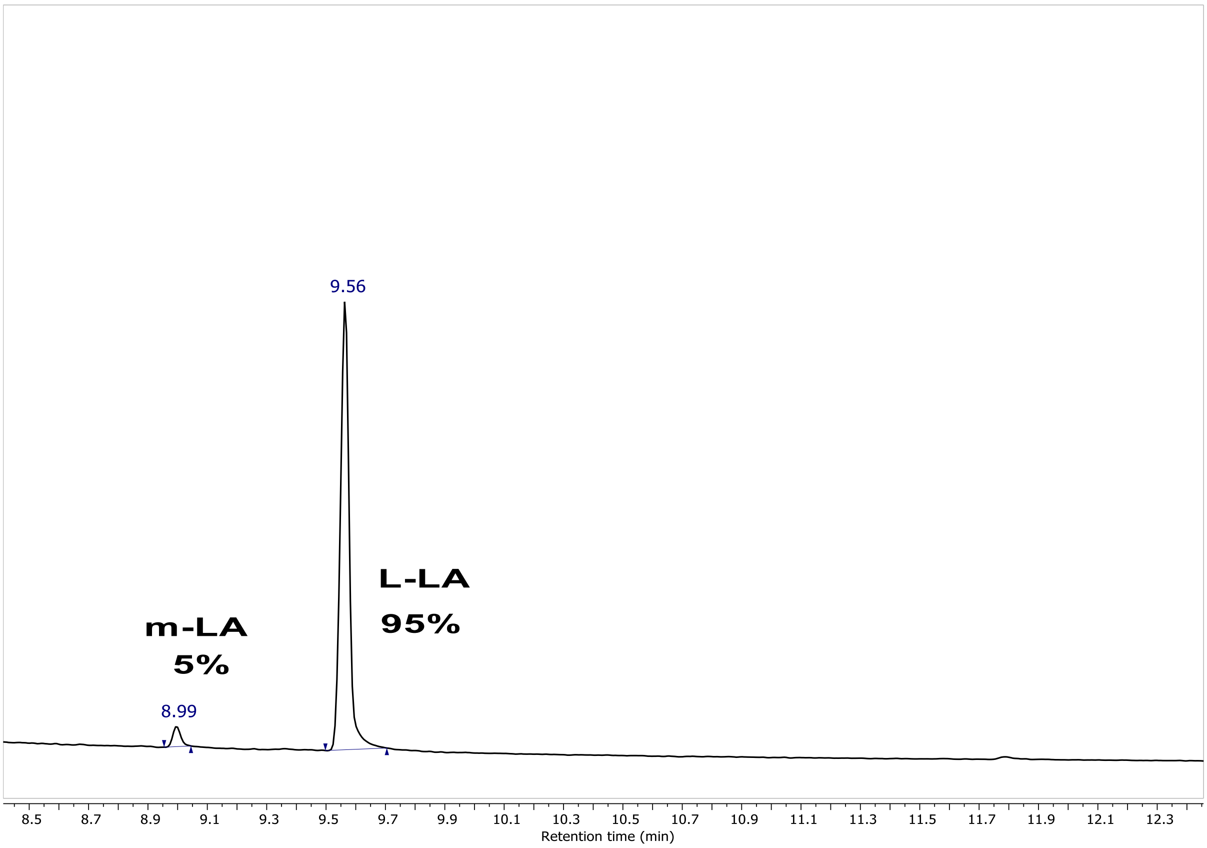

Figure S42. GC of LA isolated from depolymerization of PLLA ($[Sn(Oct)_2]_0$:$[PLLA]_0$ 0.25: 1000, 160 °C, Table 1, entry 8, *m*-LA peak 5%, *L*-LA peak 95%)

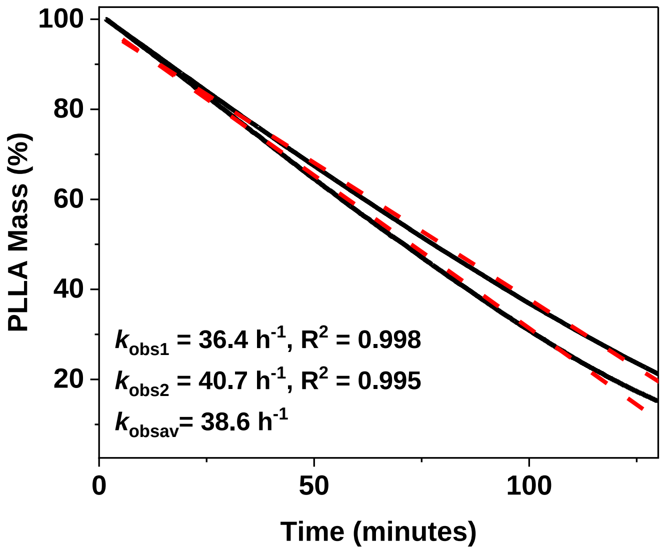

Figure S43. TGA thermograms showing repeat, independent experiments monitoring PLLA mass loss (black lines) and linear-fit of the mass loss trace (red-dashed lines) against time in $Sn(O^nBu)_2$:PLLA mixtures (1:1000, Table 1, entry 10). The TGAs were run with an isotherm at 160 °C until >95% mass loss

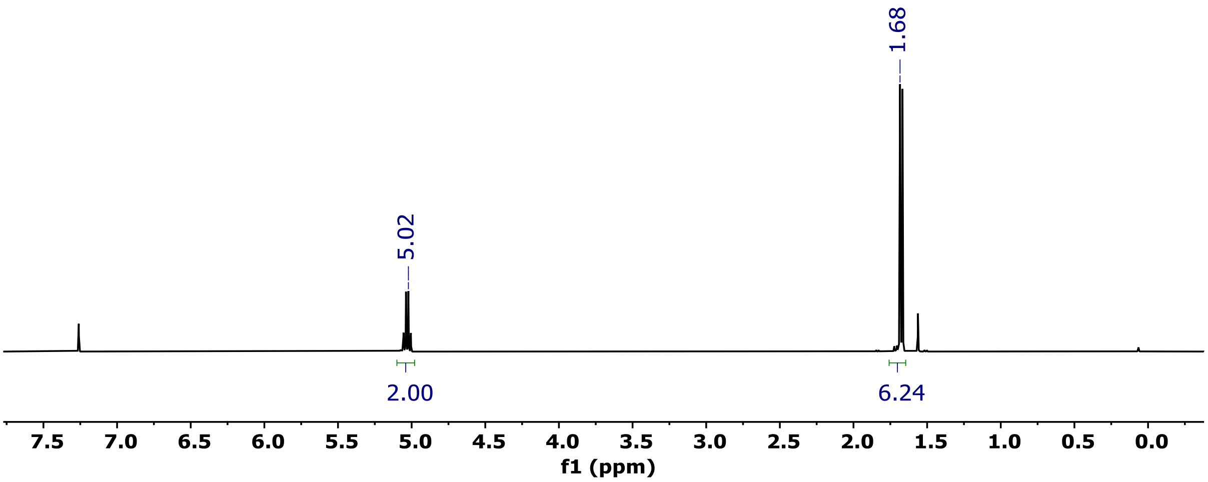

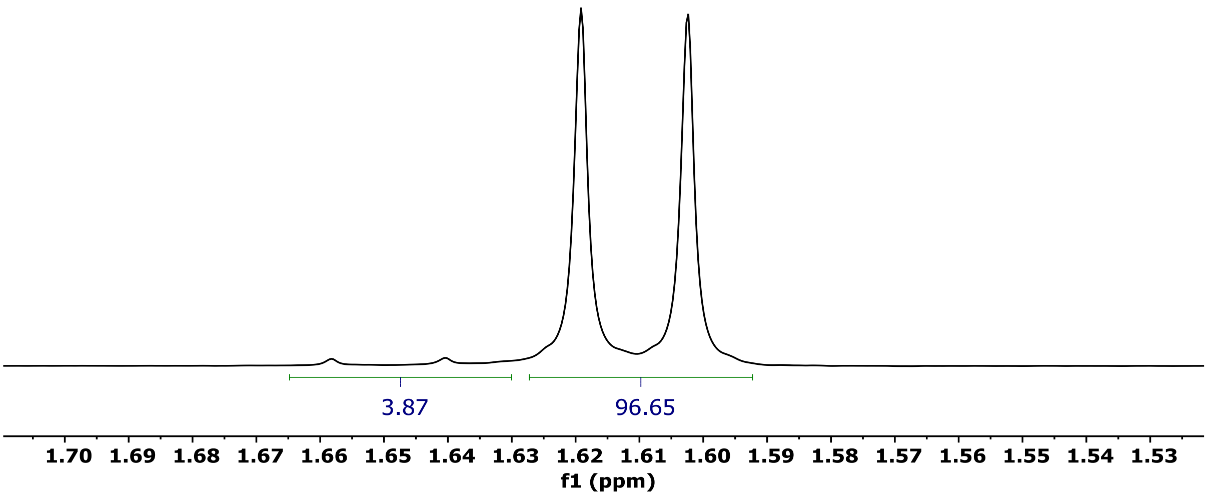

Figure S44. ^1H NMR (400 MHz, $CDCl_3$, 298K) spectrum of LA isolated from depolymerization of PLLA ($[Sn(O^nBu)_2]_0$:$[PLLA]_0$ 1:1000, 160 °C, Table 1, entry 10) δ 5.05 (q, *J* = 6.7 Hz, 2H, L,L + D,L **H**CMe), 1.66 (d, *J* = 7.1 Hz, *trace (4% meso)*, D,L HC**Me**) δ 1.60 (d, *J* = 6.7 Hz, 6H, L,L HC**Me**).

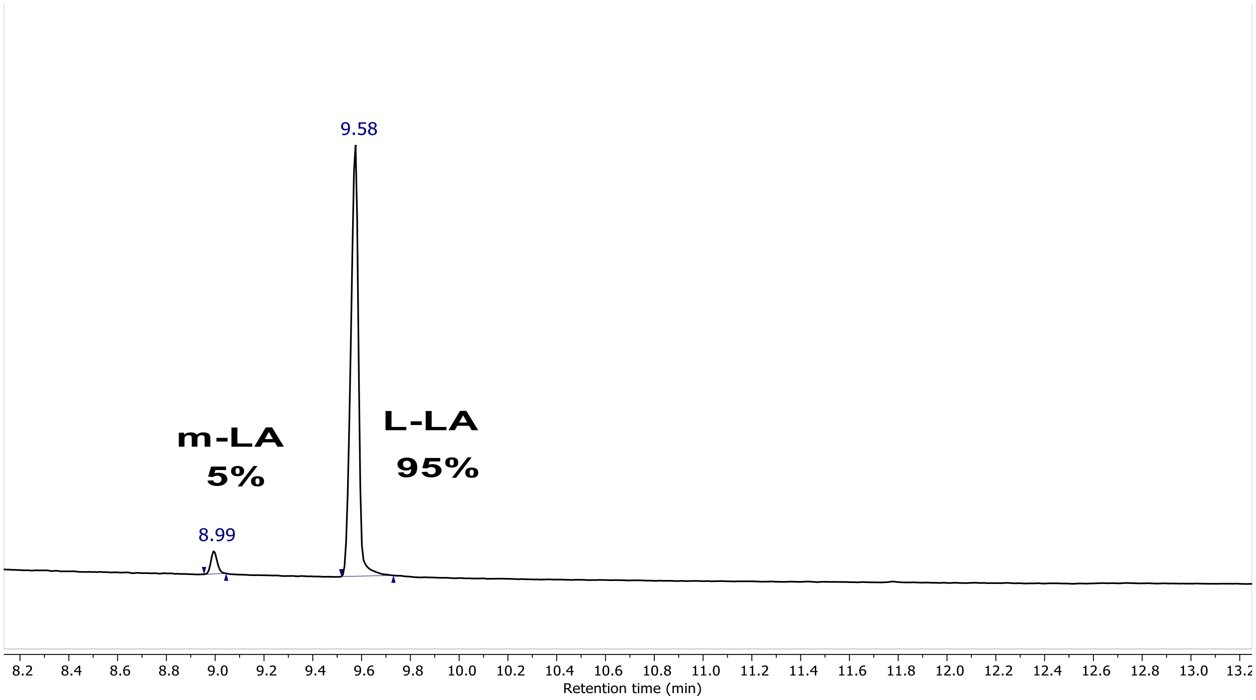

Figure S45. GC of LA isolated from depolymerization of PLLA ($[Sn(Oct)_2]_0$:$[PLLA]_0$ 1:1000, 160 °C, Table 1, entry 10, *m*-LA = 5%, *L*-LA = 95%)

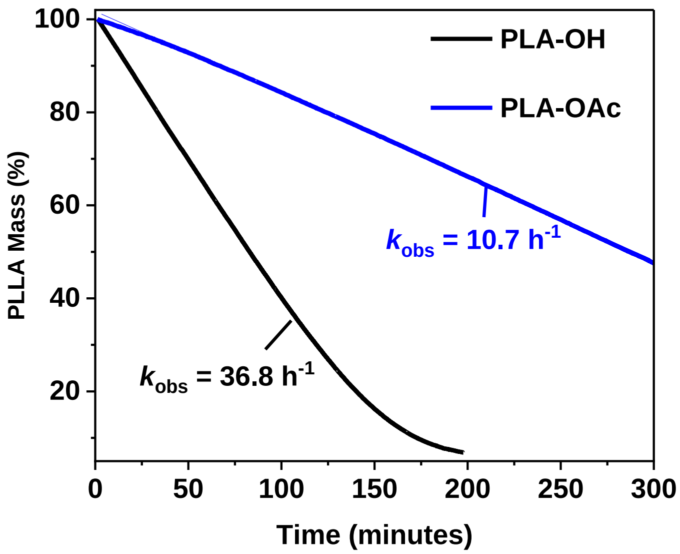

Figure S46. TGA thermograms showing mass loss against time in $Sn(Oct)_2$:PLLA-OH (black) and $Sn(Oct)_2$:PLLA-OAc mixtures (blue) (1:1000) at 160 °C

N.B. the mass loss observed with PLLA-OAc is attributed to adventitious water forming PLLA-OH chain-ends *in-situ*

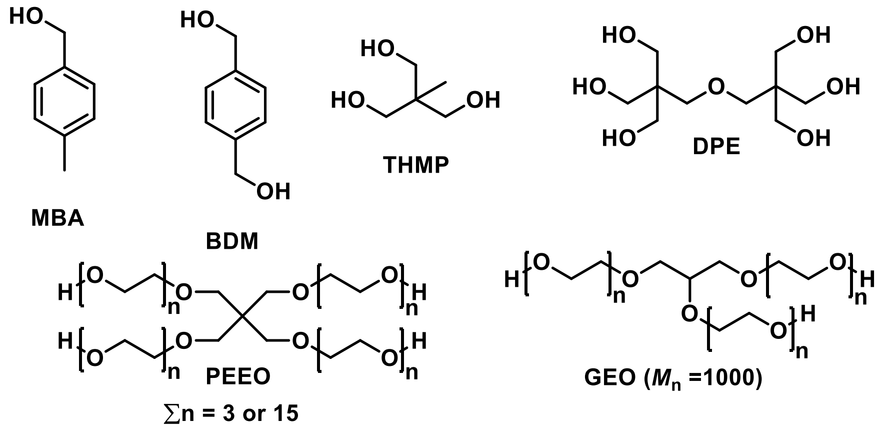

Figure S47. Structures of CTAs used in this study.

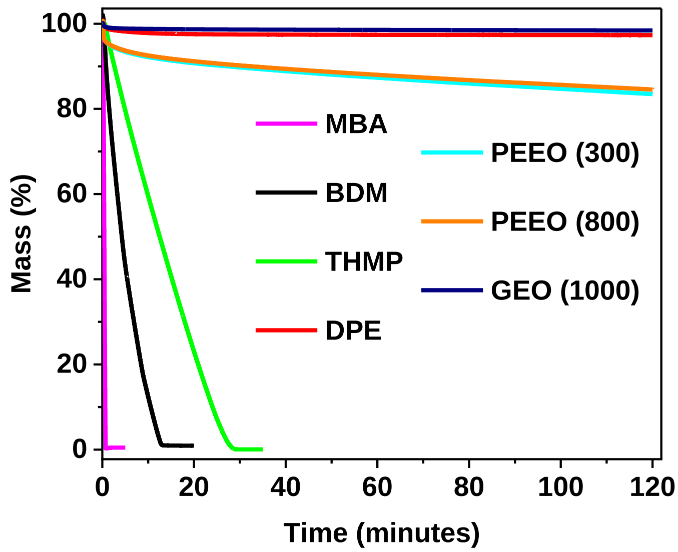

Figure S48. TGA thermograms showing mass loss of MBA (purple), BDM(black), THMP (green), DPE (red), PEEO 300 (cyan), PEEO 800 (orange) and GEO 1000 (navy) at 160 °C under N_2 flow

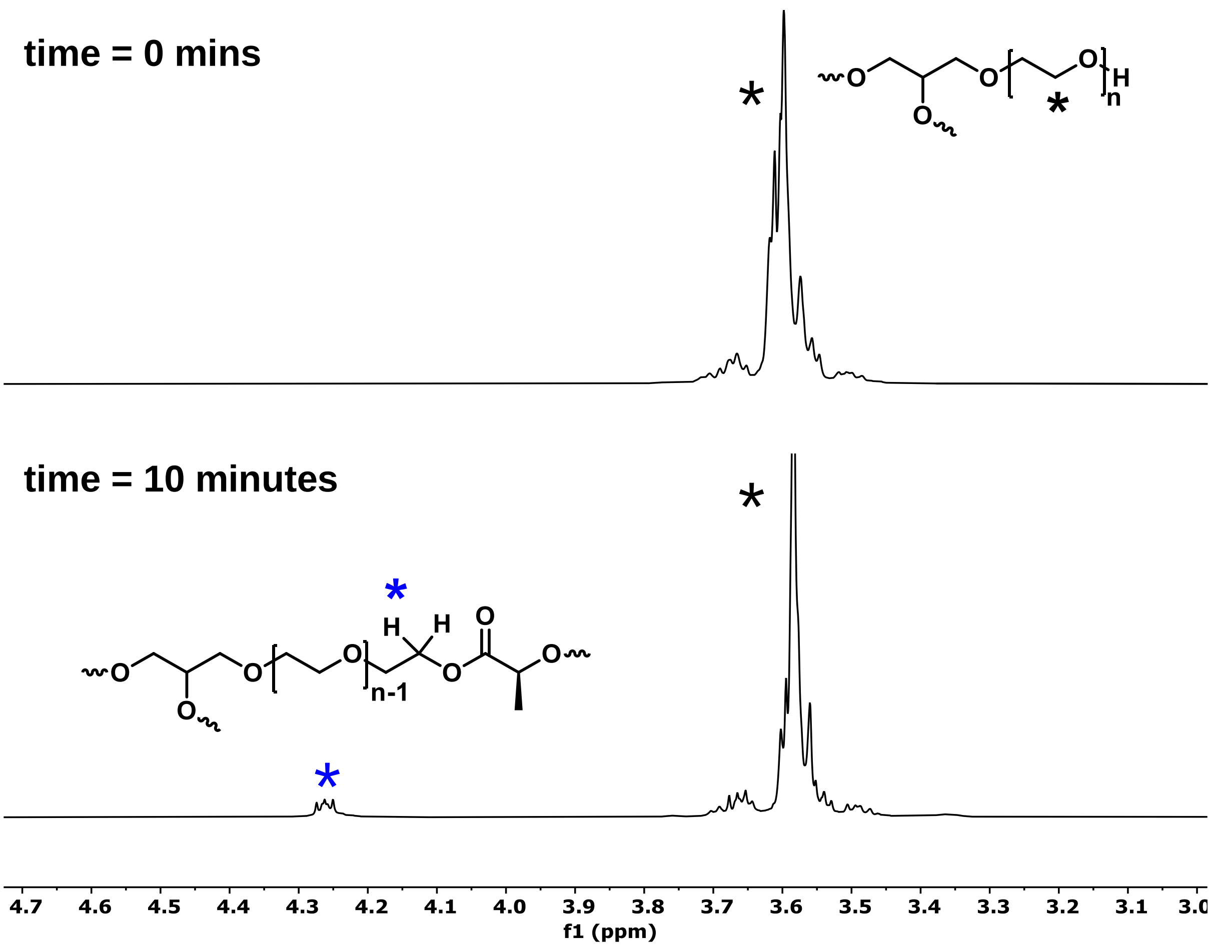

Figure S49. ^{1}H NMR spectra of GEO-PLLA transesterification reactions ($[Sn(Oct)_2]_0$:$[OH_{GEO}]_0$:$[PLLA]_0$ = 1: 20: 1000, 160 °C, neat) at time = 0 and time = 10 mins.

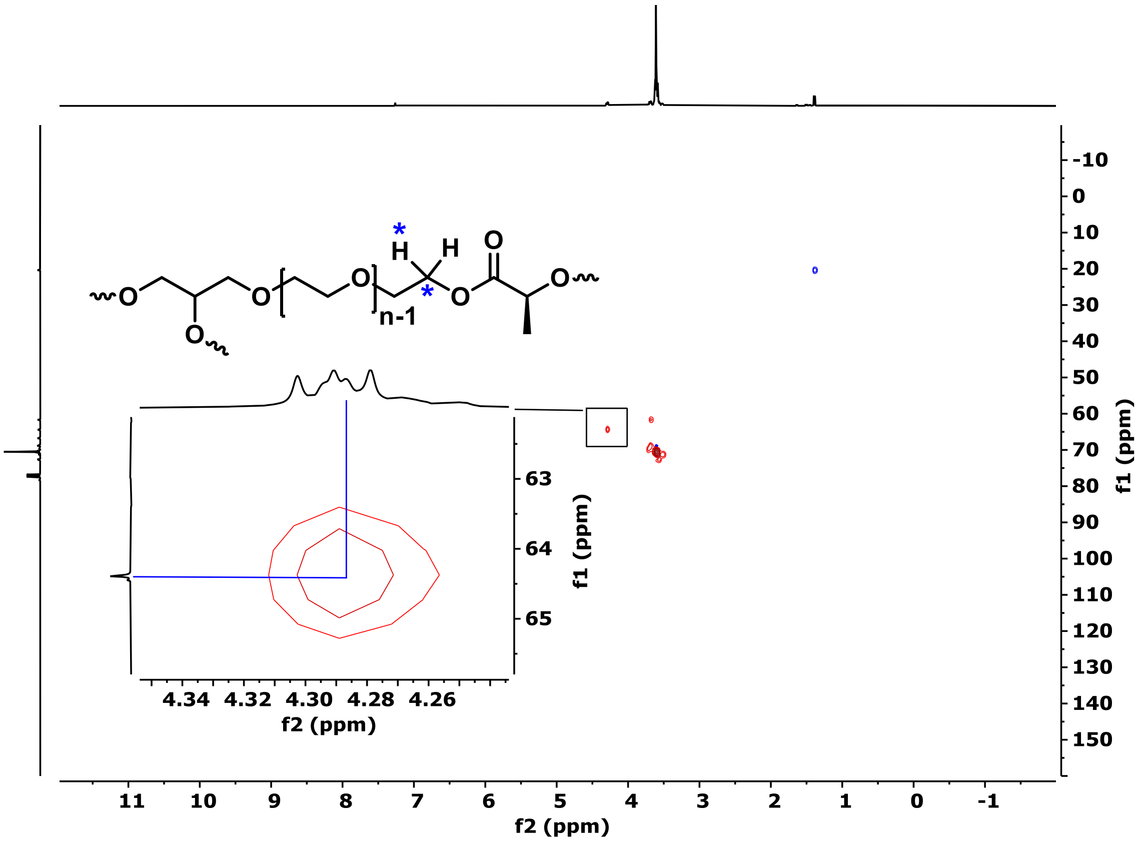

Figure S50. ^{1}H-^{13}C HSQC NMR spectrum ($CDCl_3$) of GEO-PLLA transesterification reactions ($[Sn(Oct)_2]_0$:$[OH_{GEO}]_0$:$[PLLA]_0$ = 1: 20: 1000, 160 °C, neat) at time =10 mins. Insert highlights the ^{1}H-^{13}C single-bond correlation of the GEO-junction unit

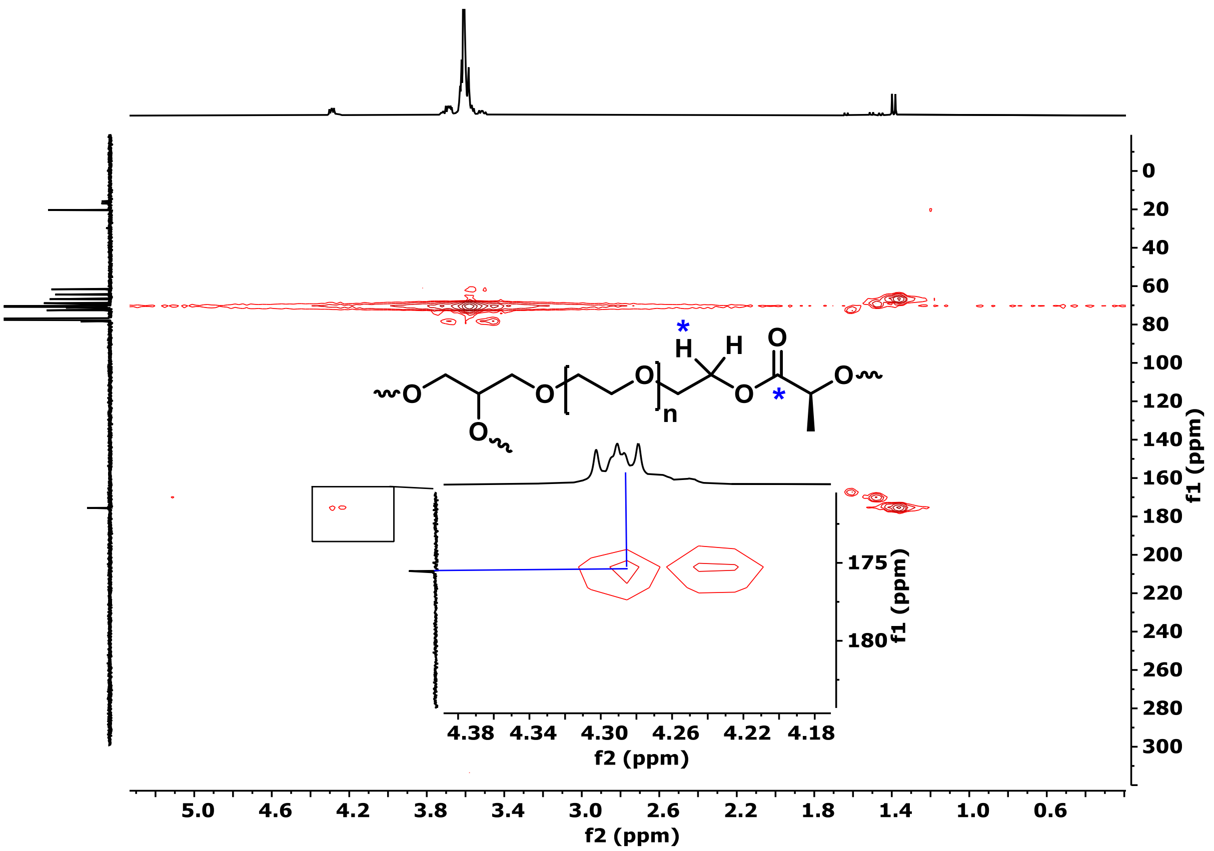

Figure S51. ^1H-^{13}C HMBC NMR spectrum ($CDCl_3$) of GEO-PLLA transesterification reactions ($[Sn(Oct)_2]_0$:$[OH_{GEO}]_0$:$[PLLA]_0$ = 1: 20: 1000, 160 °C, neat) at time =10 mins. Insert highlights the ^1H-^{13}C three-bond correlation between of the GEO-junction unit and the ester carbonyl of PLLA.

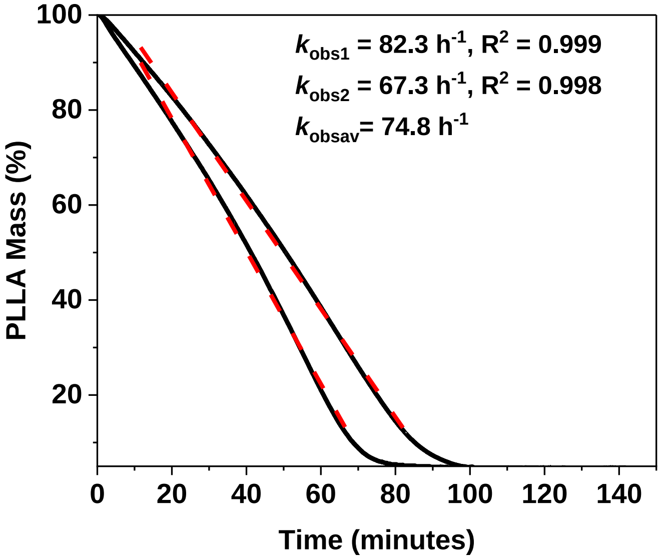

Figure S52. TGA thermograms showing repeat, independent experiments monitoring PLLA mass loss (black lines) and linear-fit of the mass loss trace (red-dashed lines) against time in $Sn(Oct)_2$:GEO:PLLA mixtures ($[Sn(Oct)_2]_0$:$[OH_{GEO}]_0$:$[PLLA]_0$ = 1: 20: 1000, Table 2, entry 1). The TGAs were run with an isotherm at 160 °C until >95% mass loss

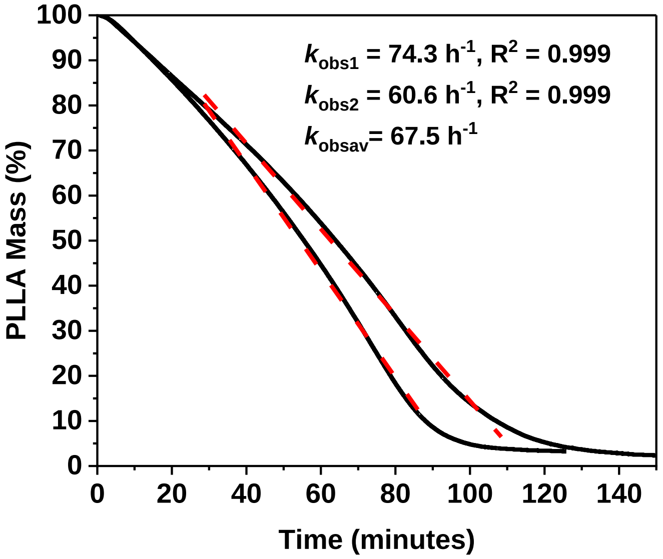

Figure S53. TGA thermograms showing repeat, independent experiments monitoring PLLA mass loss (black lines) and linear-fit of the mass loss trace (red-dashed lines) against time in $Sn(Oct)_2$:GEO:PLLA mixtures ($[Sn(Oct)_2]_0$:$[OH_{GEO}]_0$:$[PLLA]_0$ = 0.5 :20 :1000, Table 2, entry 2). The TGAs were run with an isotherm at 160 °C until >95% mass loss

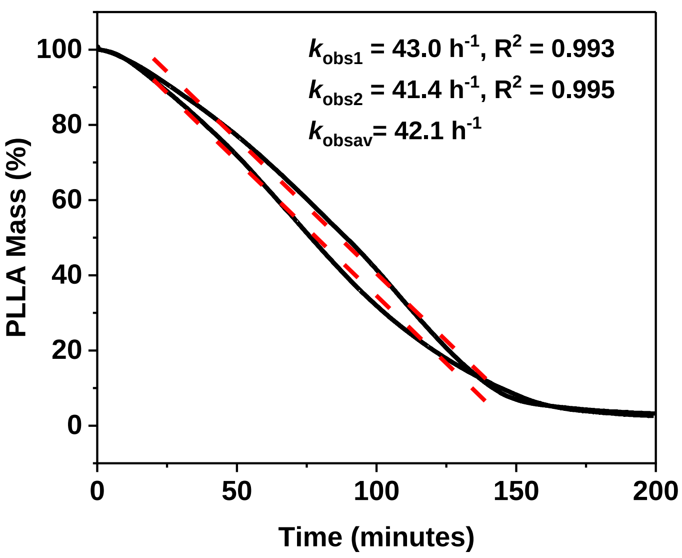

Figure S54. TGA thermograms showing repeat, independent experiments monitoring PLLA mass loss (black lines) and linear-fit of the mass loss trace (red-dashed lines) against time in $Sn(Oct)_2$:GEO:PLLA mixtures ($[Sn(Oct)_2]_0$:$[OH_{GEO}]_0$:$[PLLA]_0$ = 0.25 :20 :1000, Table 2, entry 3). The TGAs were run with an isotherm at 160 °C until >95% mass loss

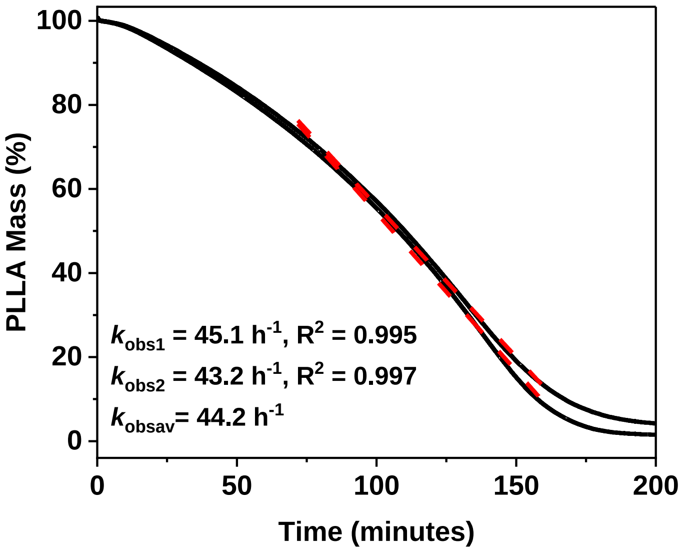

Figure S55. TGA thermograms showing repeat, independent experiments monitoring PLLA mass loss (black lines) and linear-fit of the mass loss trace (red-dashed lines) against time in $Sn(Oct)_2$:GEO:PLLA mixtures (($[Sn(Oct)_2]_0$:$[OH_{GEO}]_0$:$[PLLA]_0$ = 0.2 :20 :1000, Table 2, entry 4). The TGAs were run with an isotherm at 160 °C until >95% mass loss

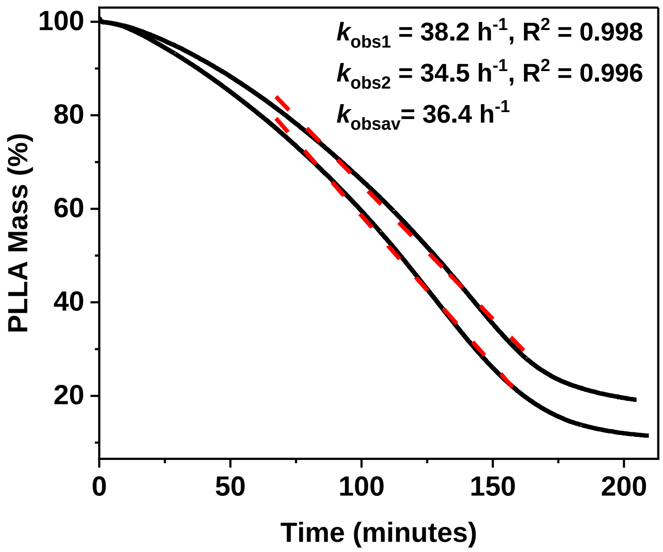

.

Figure S56. TGA thermograms showing repeat, independent experiments monitoring PLLA mass loss (black lines) and linear-fit of the mass loss trace (red-dashed lines) against time in $Sn(Oct)_2$:GEO:PLLA mixtures ($[Sn(Oct)_2]_0$:$[OH_{GEO}]_0$:$[PLLA]_0$= 0.125 :20 :1000, Table 2, entry 5). The TGAs were run with an isotherm at 160 °C until >95% mass loss

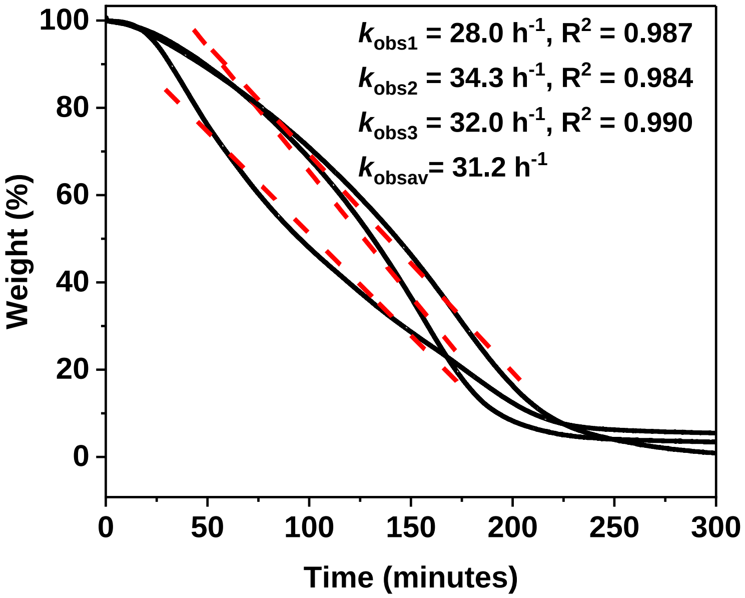

Figure S57. TGA thermograms showing repeat, independent experiments monitoring PLLA mass loss (black lines) and linear-fit of the mass loss trace (red-dashed lines) against time in $Sn(Oct)_2$:GEO:PLLA mixtures ($[Sn(Oct)_2]_0$:$[OH_{GEO}]_0$:$[PLLA]_0$ = 0.1: 20: 1000, Table 2, entry 6). The TGAs were run with an isotherm at 160 °C until >95% mass loss

Table S5. Data for $Sn(Oct)_2$-catalyzed PLLA depolymerization in the presence of GEO at fixed $[Sn(Oct)_2]_0$

Entry	$[cat]:[OH_{CTA}]_0:[PLA]_0$[a]	Rate constant k_{obs}^{GEO} (h^{-1})[b]	Activity, TOF (h^{-1})[c]	Mass Loss rate ($g\ g^{-1}\ h^{-1}$)[d]	Selectivity % *L*-LA[e]	Rate Enhancement k_{obs}^{GEO}/k_{obs} [f]
1	0.25: 10: 1000	35.1(±3.2)	1350(±100)	220(±17)	99	3.0(±0.1)
2	0.25: 20: 1000	42.1(±2.4)	1590(±117)	285(±21)	99	4.3(±0.1)
3	0.25: 30: 1000	90.1(±5.1)	3000(±220)	534(±40)	99	9.2(±0.1)
4	0.25: 40: 1000	74.9(±4.3)	2700(±200)	474(±36)	99	7.6(±0.2)
5	0.25: 50: 1000	84.8 (±15.1)	3000 (±380)	540 (±67)	99	8.0 (±0.1)
6	0.25: 80: 1000	86.5(±10.0)	2800(±200)	491(±38)	99	8.7(±0.2)

Depolymerization experiments conducted using thin films, analysed using TGA over 5 h or until >95% mass loss (see SI for details of experimental setup). [a] Catalyst loadings determined per lactic acid repeat unit (M_r = 72.06 g mol^{-1}) and $[OH_{GEO}]_0 = 3[GEO]_0$. [b] Rate constant determined as the gradient of linear fits to plots of mass loss vs time. Error ranges are determined from multiple repeat runs. [c] TOF = Activity as TOF defined as moles of lactic acid repeat units consumed from 0-80% mass loss/moles of catalyst/time taken for 0-80% mass loss. Average errors were taken from repeat runs. [d] Mass loss rate = TOF × M_r of PLLA repeat unit (72.06)/M_r of catalyst. [e] Selectivity for monomer, %*L*-LA, was determined by ^1H NMR spectroscopy and GCMS using monomer isolated from sublimation depolymerization experiments (see SI for details). 5% meso-lactide formed from 5% D-lactic acid repeat units in PLLA sample.[f] Rate enhancement when using intermolecular transesterification strategy determined as k_{obs}^{GEO}/k_{obs} where values are reported at constant catalyst loading and reaction conditions.

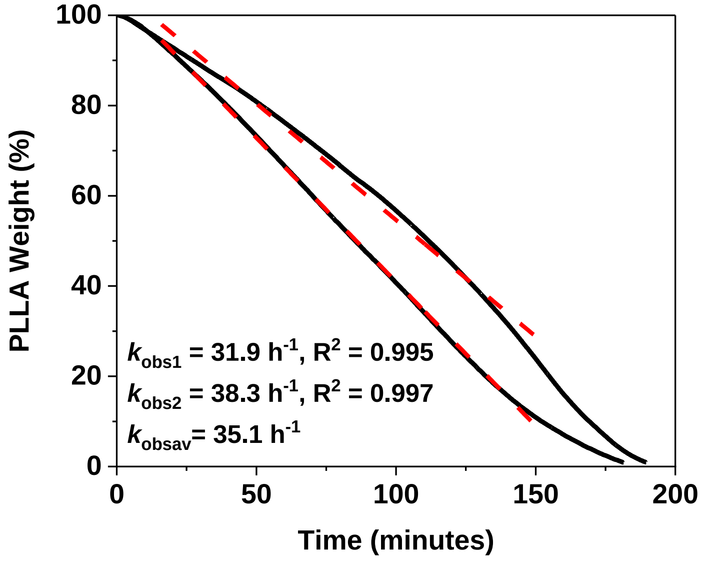

Figure S58. TGA thermograms showing repeat, independent experiments monitoring PLLA mass loss (black lines) and linear-fit of the mass loss trace (red-dashed lines) against time in $Sn(Oct)_2$:GEO:PLLA mixtures (($[Sn(Oct)_2]_0$:$[OH_{GEO}]_0$:$[PLLA]_0$ = 0.25: 10: 1000, Table 2, entry 7 and Table S5, entry 1). The TGAs were run with an isotherm at 160 °C until >95% mass loss

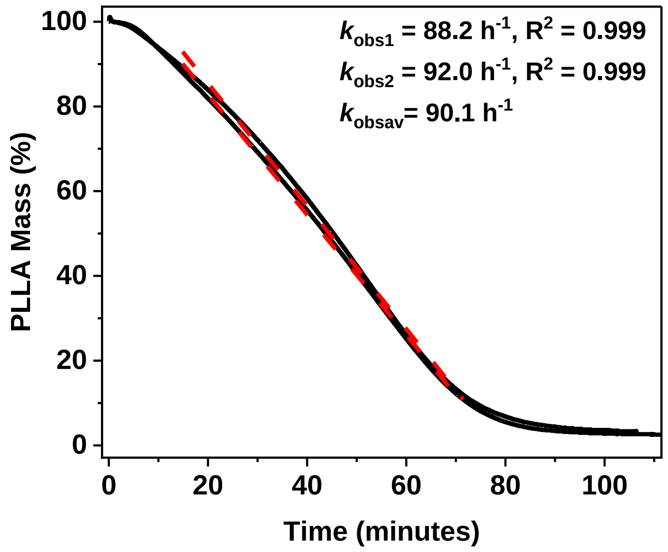

Figure S59. TGA thermograms showing repeat, independent experiments monitoring PLLA mass loss (black lines) and linear-fit of the mass loss trace (red-dashed lines) against time in $Sn(Oct)_2$:GEO:PLLA mixtures ($[Sn(Oct)_2]_0$:$[OH_{GEO}]_0$:$[PLLA]_0$ = 0.25 :30 :1000, Table 2, entry 8 and Table S5, entry 3). The TGAs were run with an isotherm at 160 °C until >95% mass loss

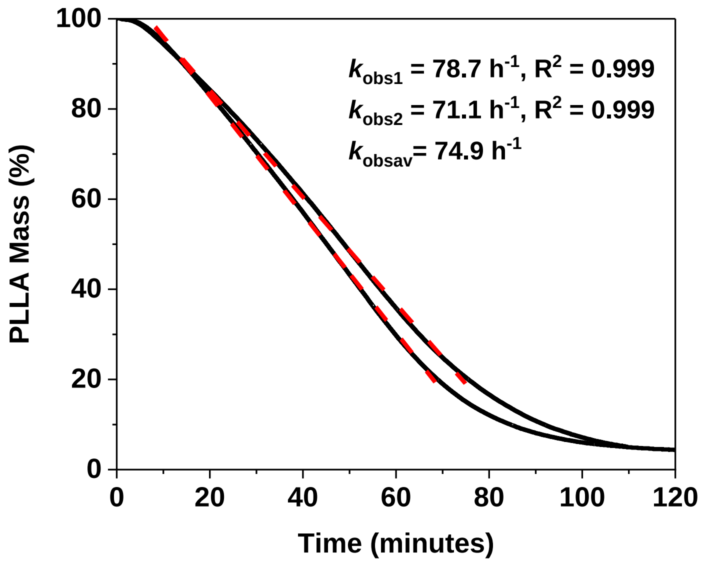

Figure S60. TGA thermograms showing repeat, independent experiments monitoring PLLA mass loss (black lines) and linear-fit of the mass loss trace (red-dashed lines) against time in $Sn(Oct)_2$:GEO:PLLA mixtures ($[Sn(Oct)_2]_0$:$[OH_{GEO}]_0$:$[PLLA]_0$ = 0.25: 40: 1000, Table S5, entry 4). The TGAs were run with an isotherm at 160 °C until >95% mass loss

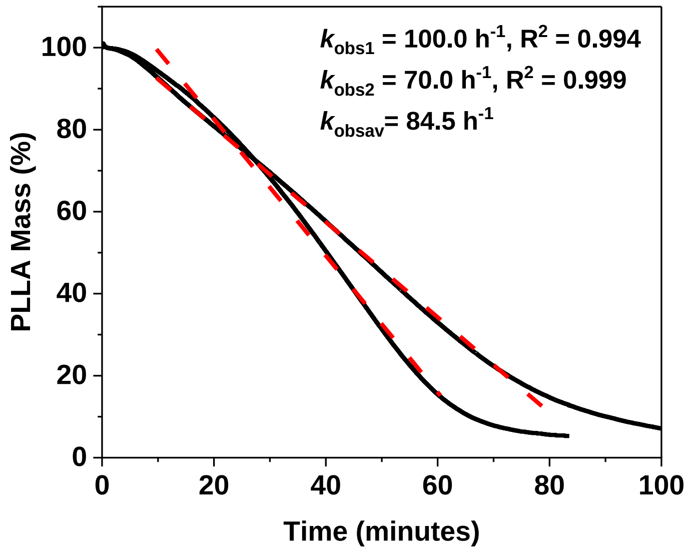

Figure S61. TGA thermograms showing repeat, independent experiments monitoring PLLA mass loss (black lines) and linear-fit of the mass loss trace (red-dashed lines) against time in $Sn(Oct)_2$:GEO:PLLA mixtures ($[Sn(Oct)_2]_0$:$[OH_{GEO}]_0$:$[PLLA]_0$ = 0.25 :50 :1000, Table S5, entry 5). The TGAs were run with an isotherm at 160 °C until >95% mass loss

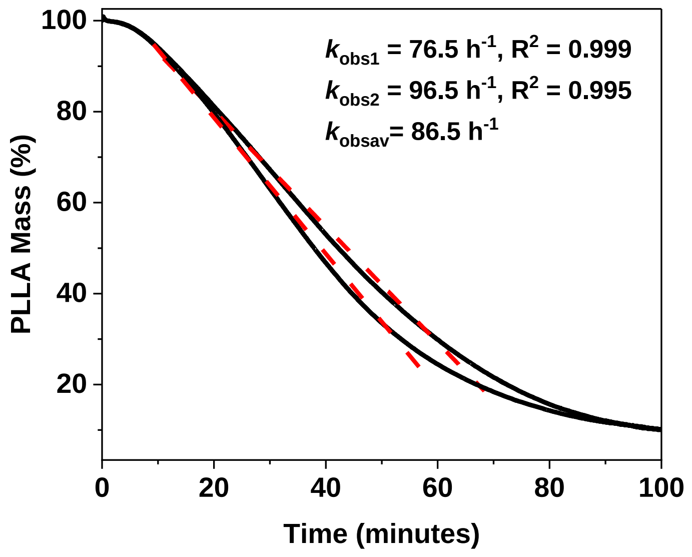

Figure S62. TGA thermograms showing repeat, independent experiments monitoring PLLA mass loss (black lines) and linear-fit of the mass loss trace (red-dashed lines) against time in $Sn(Oct)_2$:GEO:PLLA mixtures ($[Sn(Oct)_2]_0$:$[OH_{GEO}]_0$:$[PLLA]_0$ = 0.25: 80:1000, Table 2, entry 9 and Table S5, entry 6). The TGAs were run with an isotherm at 160 °C until >95% mass loss

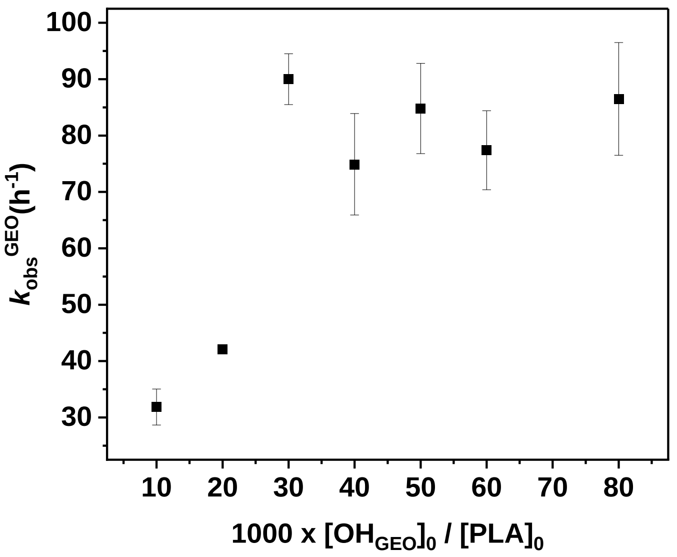

Figure S63. k_{obs}^{GEO} *vs* $[OH_{GEO}]_0$/$[PLLA]_0$. Depolymerizations conducted at 160 °C with varied $[OH_{GEO}]_0$ and $[Sn(Oct)_2]_0$:$[PLLA]_0$ = 0.25:1000

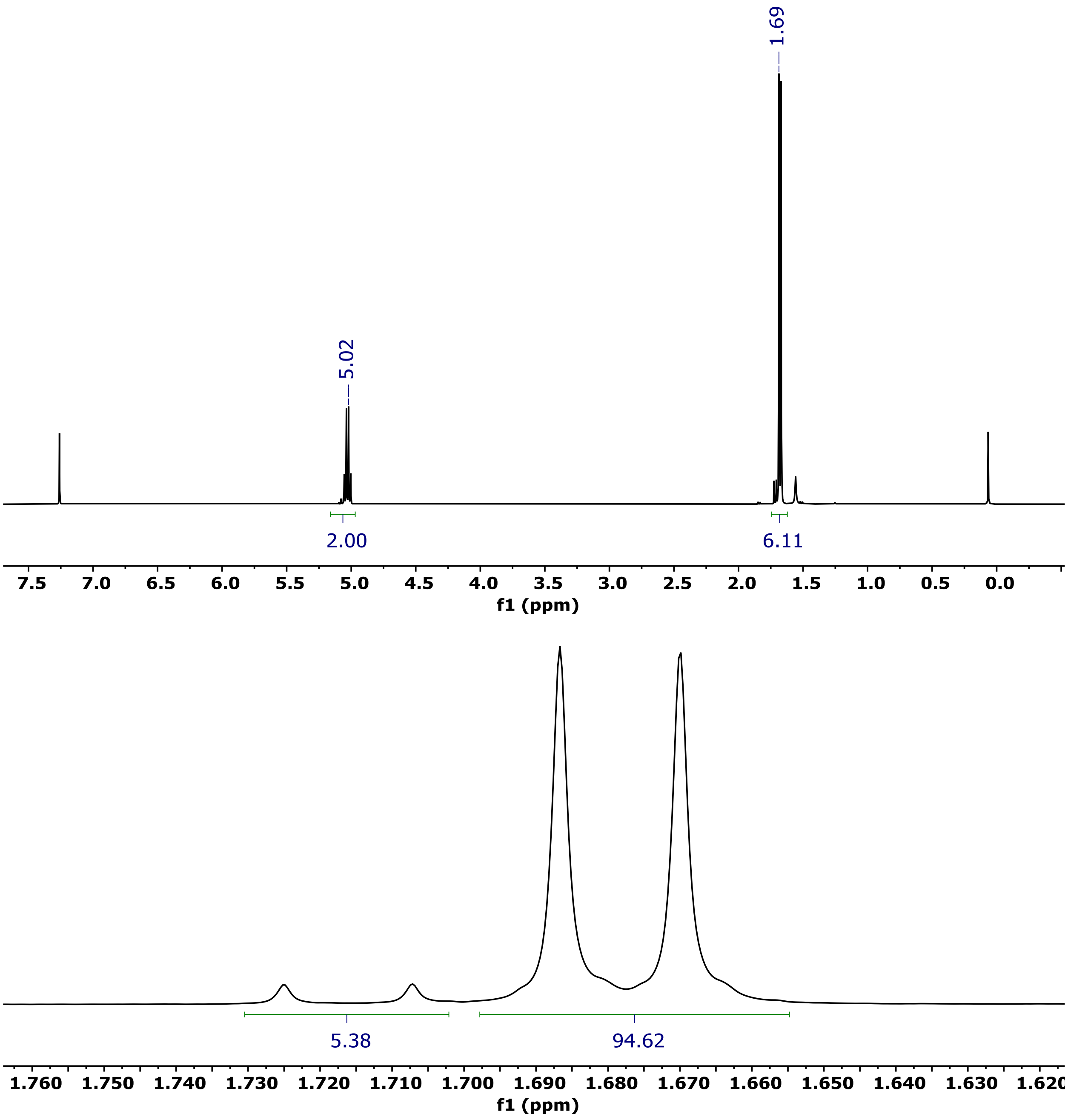

Figure S64. ^{1}H NMR ($CDCl_3$, 298K) spectrum of LA isolated from depolymerization of PLLA from mixtures of $Sn(Oct)_2$, GEO and PLLA ($[Sn(Oct)_2]_0$:$[OH_{GEO}]_0$:$[PLLA]_0$ = 0.1: 20:1000,160 °C, Table 2, entry 6)

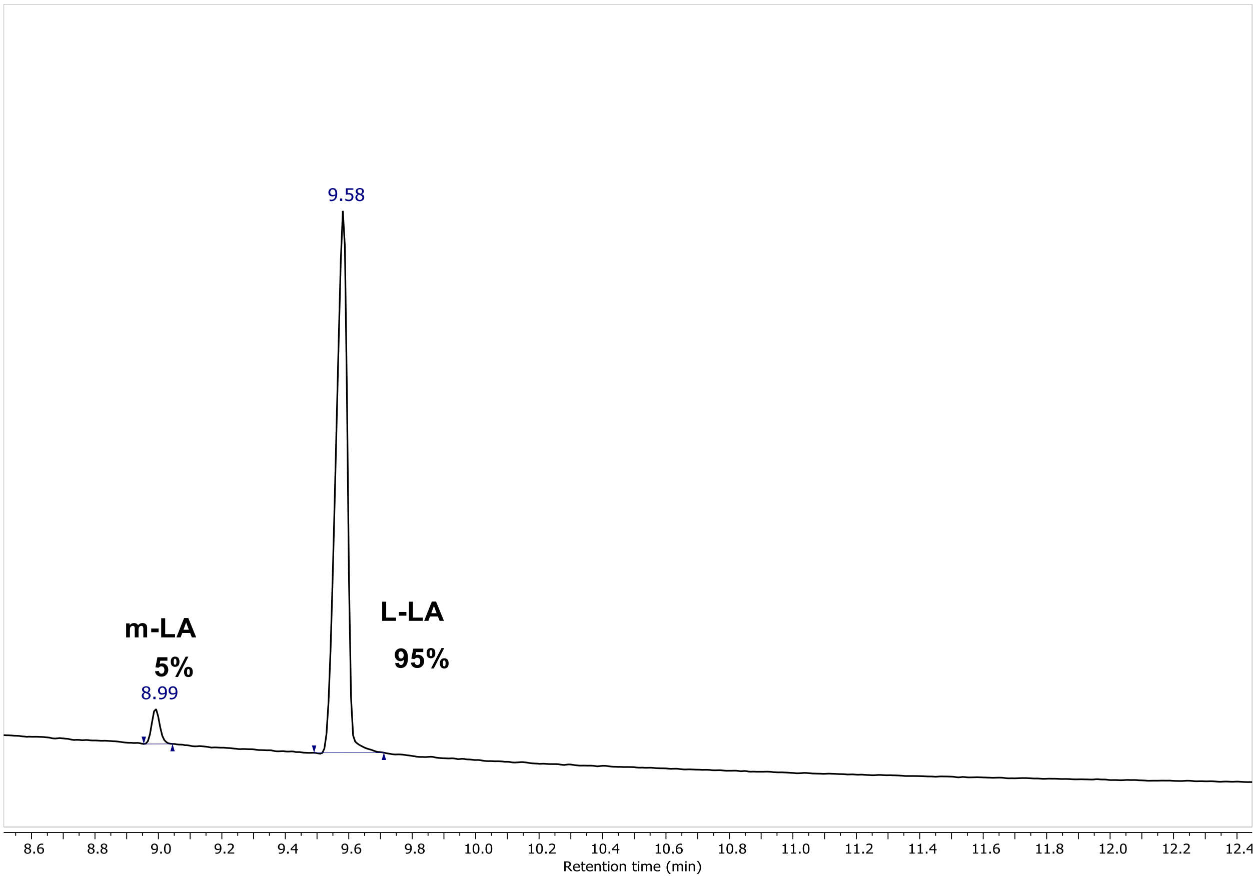

Figure S65. GC of LA isolated from depolymerization of PLLA from mixtures of $Sn(Oct)_2$, GEO and PLLA ($[Sn(Oct)_2]_0$:$[OH_{GEO}]_0$:$[PLLA]_0$ = 0.1: 20:1000, Table 2, entry 6, 160 °C, *m*-LA = 5%, *L*-LA = 95%)

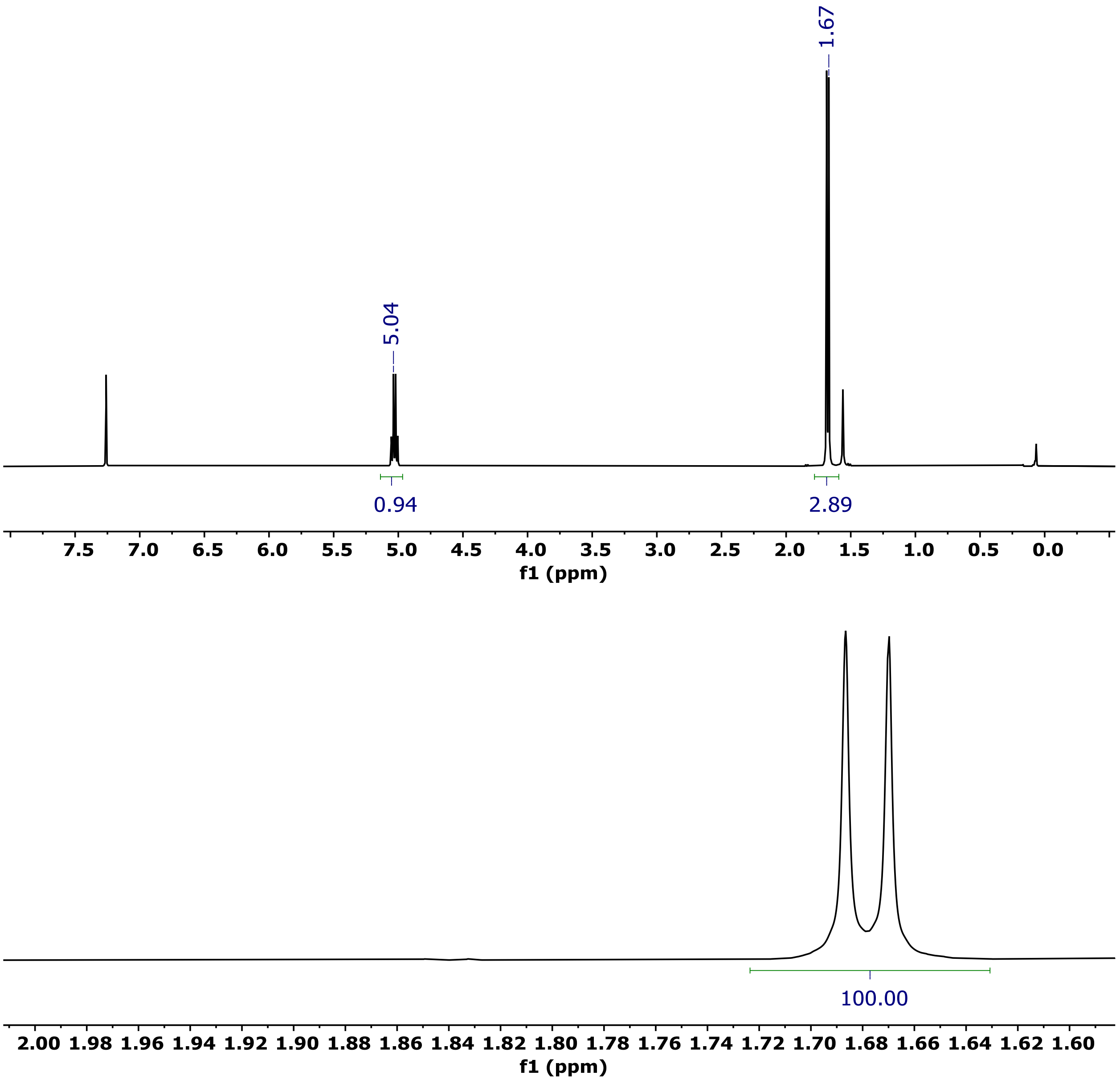

Figure S66. ^{1}H NMR ($CDCl_3$, 298K) spectrum of LA isolated from depolymerization of PLLA from mixtures of $Sn(Oct)_2$, GEO and 100% PLLA ($[Sn(Oct)_2]_0$:$[OH_{GEO}]_0$:$[PLLA]_0$ = 0.1: 20:1000,160 °C)

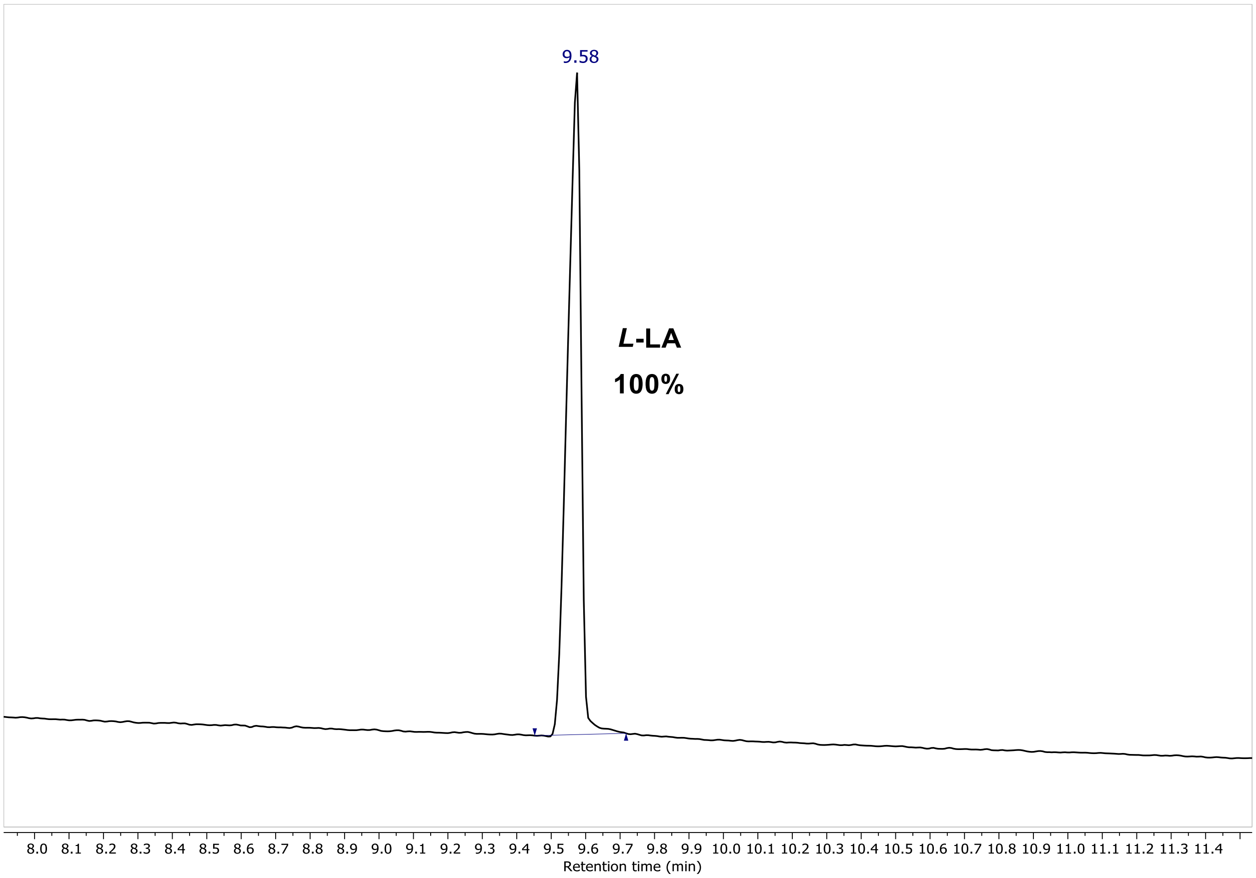

Figure S67. GC of LA isolated from depolymerization of PLLA from mixtures of $Sn(Oct)_2$, GEO and 100%-PLLA ($[Sn(Oct)_2]_0$:$[OH_{GEO}]_0$:$[PLLA]_0$ = 0.1: 20:1000, 160 °C, *L*-LA = 100%)

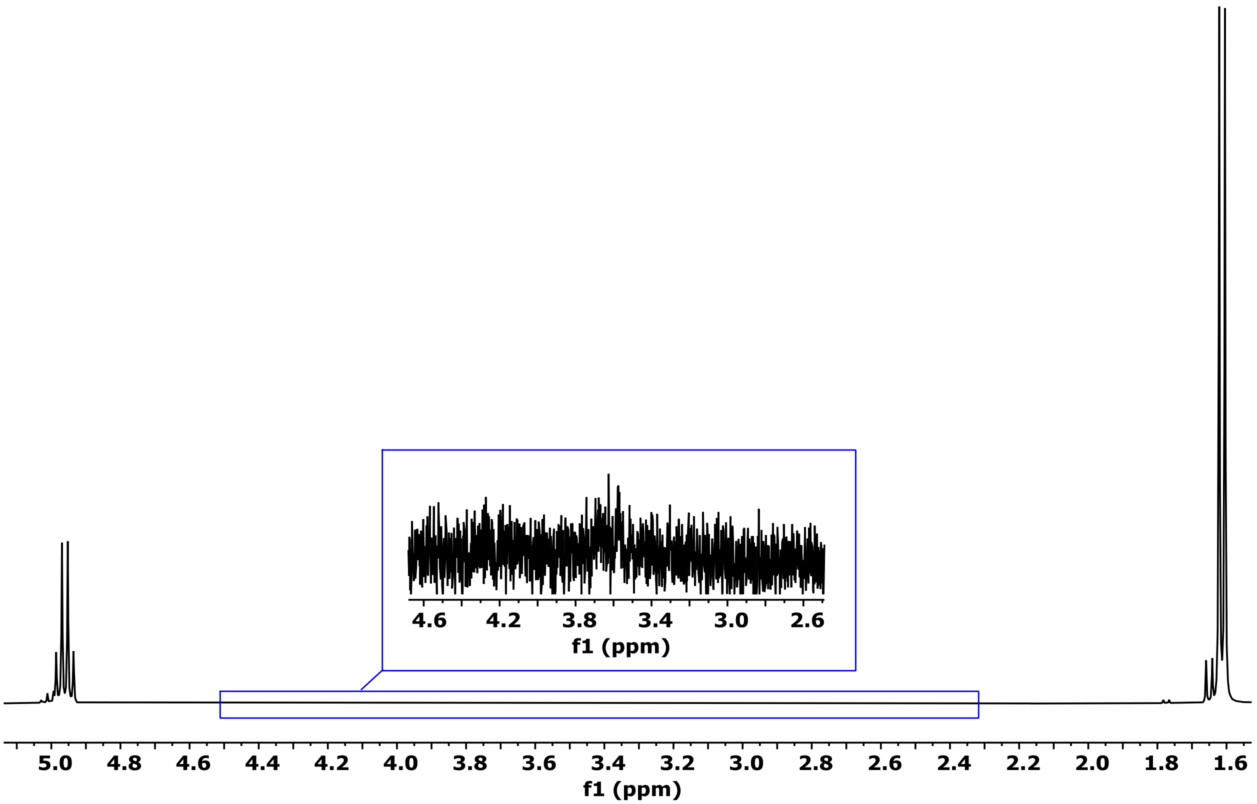

Figure S68. ^{1}H NMR (400 MHz, $CDCl_3$, 298K) spectrum of LA isolated from depolymerization of PLLA from mixtures of $Sn(Oct)_2$, GEO and PLLA ($[Sn(Oct)_2]_0$:$[OH_{GEO}]_0$: $[PLLA]_0$, 0.1: 20: 1000, 160 °C). GEO or GEO-like resonance not detected

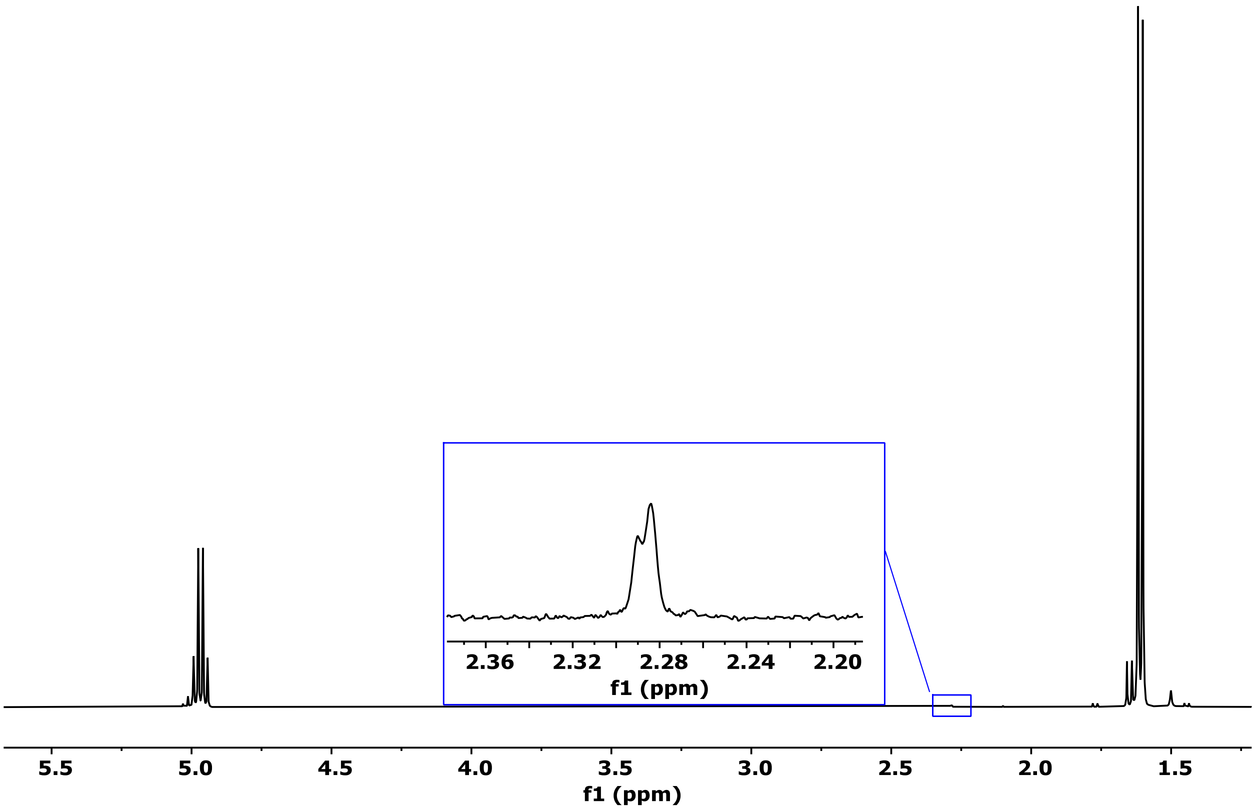

Figure S69. ^{1}H NMR (400 MHz, $CDCl_3$, 298K) spectrum of LA isolated from depolymerization of PLLA from mixtures of $Sn(Oct)_2$, MBA and PLLA ($[Sn(Oct)_2]_0$:$[OH_{MBA}]_0$: $[PLLA]_0$ 1: 20: 1000, 160 °C). MBA-like contaminant highlighted in insert

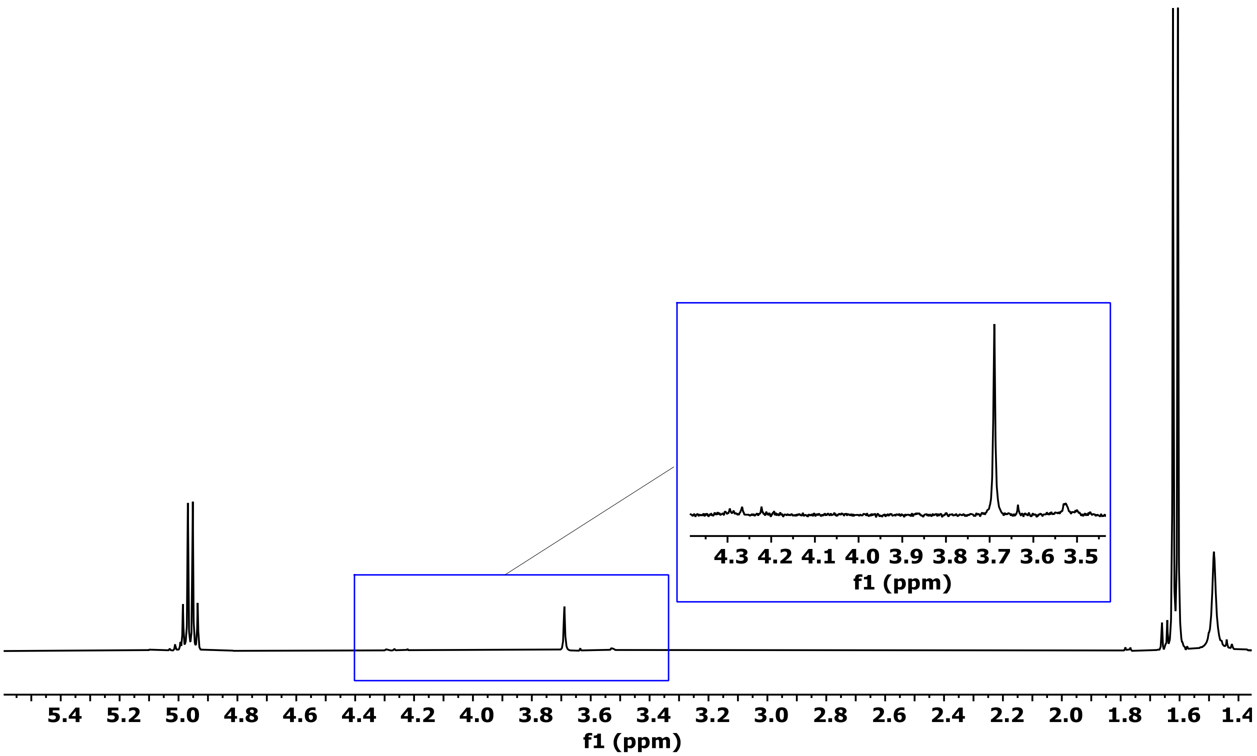

Figure S70. ^1H NMR (400 MHz, $CDCl_3$, 298K) spectrum of LA isolated from depolymerization of PLLA from mixtures of $Sn(Oct)_2$, THMP and PLLA mixtures ($[Sn(Oct)_2]_0$:$[OH_{THMP}]_0$: $[PLLA]_0$ 1: 20: 1000, 160 °C). THMP-like contaminant highlighted in insert

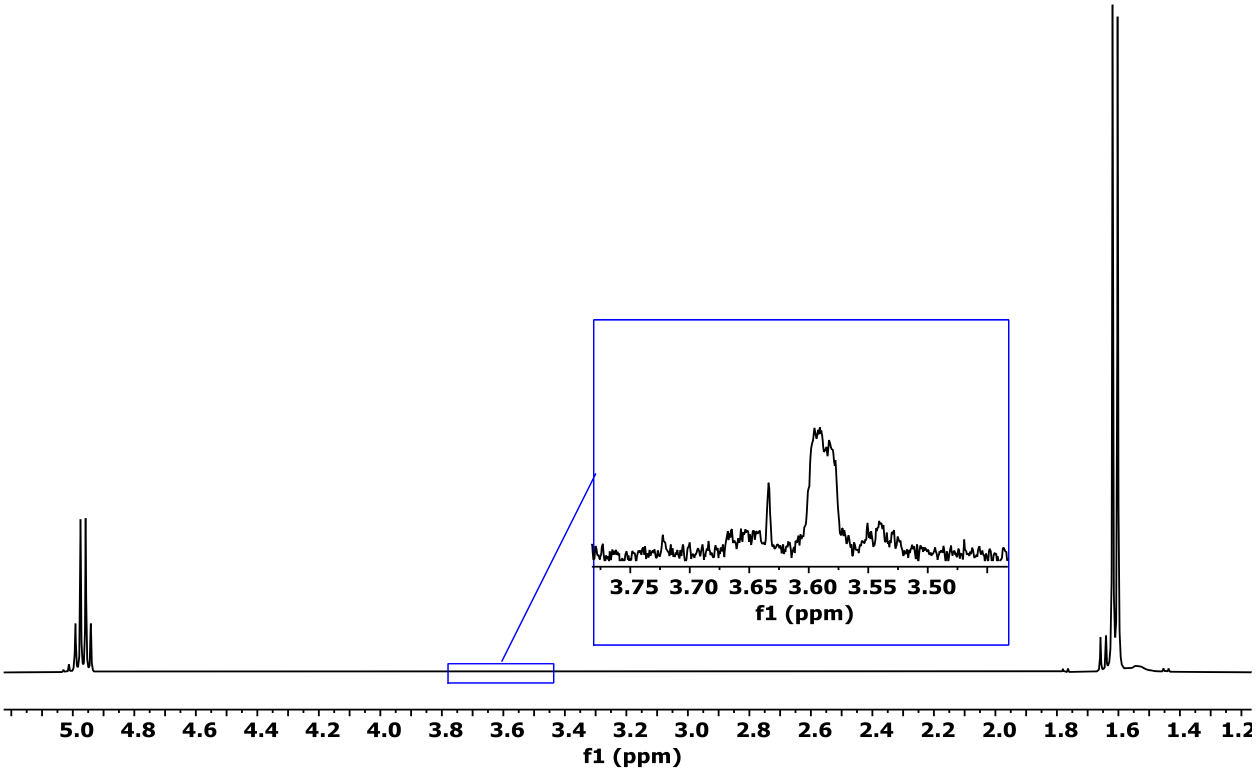

Figure S71. ^{1}H NMR (400 MHz, $CDCl_3$, 298K) spectrum of LA isolated from depolymerization of PLLA from mixtures of $Sn(Oct)_2$, PEEO and PLLA ($[Sn(Oct)_2]_0$:$[OH_{PEEO}]_0$: $[PLLA]_0$ 1: 20: 1000, 160 °C) PEEO-like contaminant highlighted in insert.

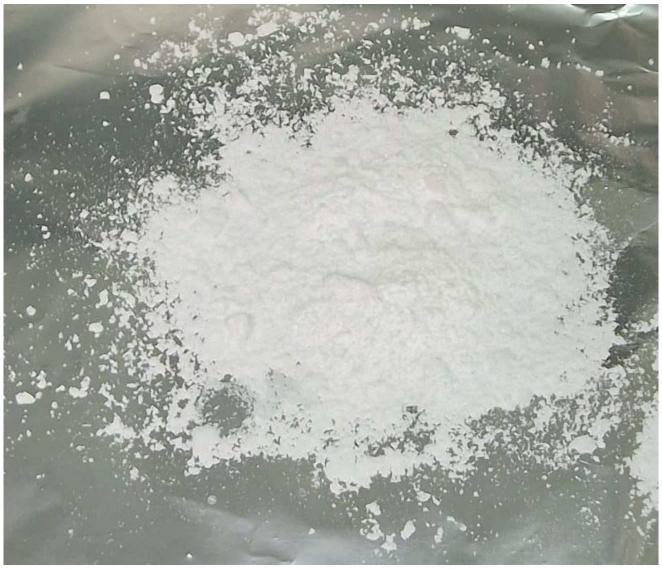

Figure S72. Representative *L*-LA isolated from depolymerization of PLLA using $Sn(Oct)_2$ and GEO catalyst

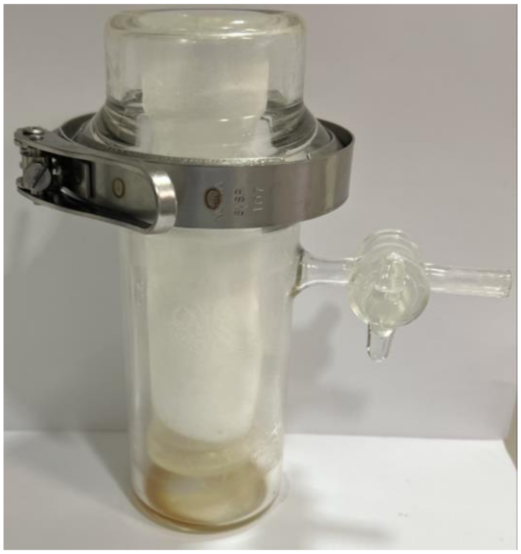

Figure S73. Sublimation kit for depolymerization of 10.0 g of PLLA using $Sn(Oct)_2$ and GEO ($[Sn(Oct)_2]_0$:$[OH_{GEO}]_0$: $[PLLA]_0$ 0.0625: 20: 10000, 160 °C)

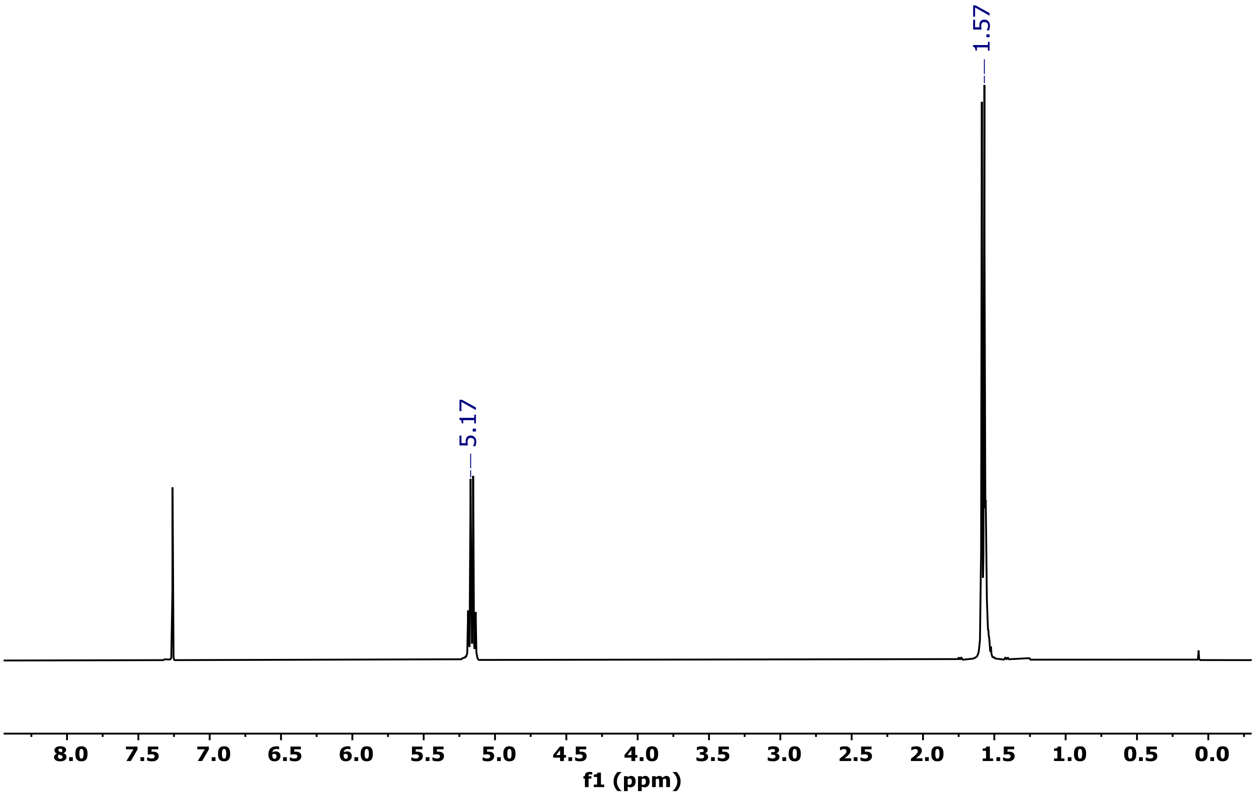

Figure S74. ^{1}H NMR spectrum (400 MHz, $CDCl_3$, 298 K) of commercial PLLA (5%-meso, see Fig. S2) used for study, δ 5.09 (q, J = 7.1 Hz, 2H, <u>H</u>CMe) and δ 1.51 (d, J = 7.2 Hz, 6H, HC<u>Me</u>).

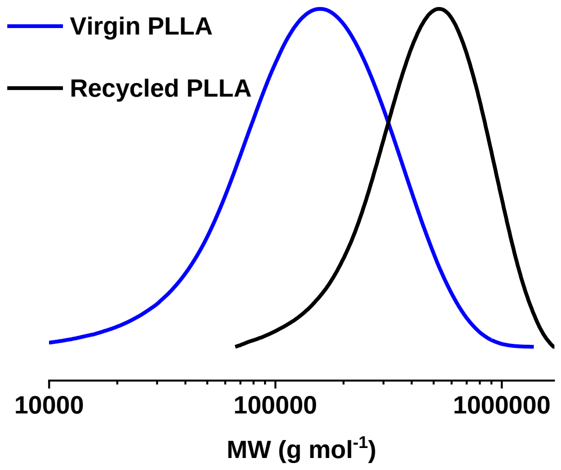

Figure S75. SEC traces for virgin (blue) and recycled PLLA (black). Recycled PLLA molar mass 230,000 g mol^{-1} (correction factor of 0.58 applied) and $Đ_M$ =1.34.

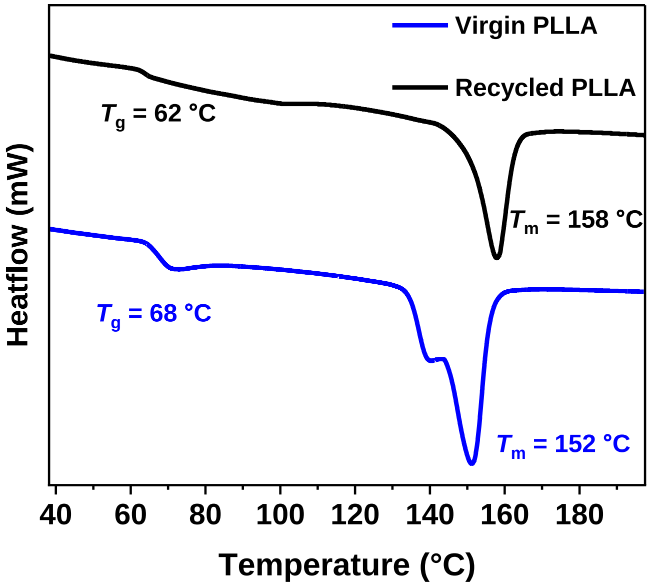

Figure S76. DSC thermogram of virgin PLLA and recycled PLLA.

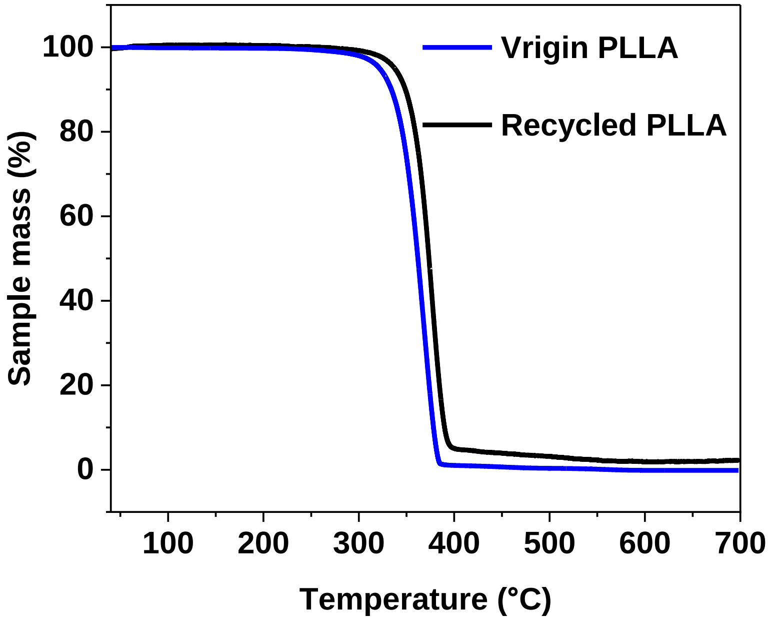

Figure S77. TGA thermogram of of virgin PLLA and recycled PLLA

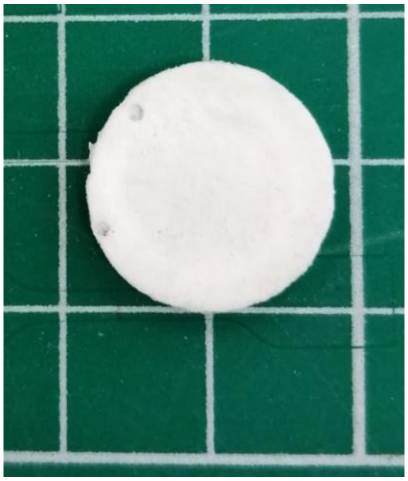

Figure S78. Photograph of recycled PLLA

4.0 References

[1] R. Gsell, M. Zeldin, *J. Inorg. Nucl. Chem.* **1975**, *37*, 1133-1137.
[2] A. Kowalski, A. Duda, S. Penczek, *Macromolecules* **1998**, *31*, 2114-2122.
[3] A. Spyros, D. S. Argyropoulos, R. H. Marchessault, *Macromolecules* **1997**, 30, 2, 327–329
